# Supplementary material for: Vibroscape analysis reveals acoustic niche overlap and plastic alteration of vibratory courtship signals in ground-dwelling wolf spiders
Source: Commun Biol. 2024 Jan 5;7:23. doi: 10.1038/s42003-023-05700-6 (PMC10770364; doi:10.1038/s42003-023-05700-6)
Supplement: Supplementary file 2 — Supplementary Material S1 [file 42003_2023_5700_MOESM2_ESM.pdf]

**Supplementary Materials**

***S1. Verification of automated background noise filtering and detection algorithms***

Given the large spatial and temporal variation in background noise, our adaptive noise filtering and detection methods may induce arbitrary variation in the quantification of acoustic properties (e.g. dominant frequency, peak rate) or the performance to detect signals. Thus, we verified the possibility of the distortion of acoustic properties by our audio processing through the simulation of the filtering and detection process with different background noise profiles using *Schizocosa* wolf spider's signals detected from our field recordings (n=7422; before bout grouping by 1-minute criteria).

*Verification of adaptive noise filtering* - For the verification of adaptive noise filtering, we compared the acoustic properties of *Schizocosa* wolf spider's signals (i.e. dominant frequency, zero-crossing rates, spectral centroids, and spectral bandwidth) after filtering simulated 'noisy' audio by the addition of generated background noise that varies in (i) the frequency range and (ii) the signal-to-noise ratio (SNR).

To test the effects of the frequency range of background noise, we generated 10 different background noise profiles by the addition of 100 Hz band-limited noise ranging from 0 – 1000 Hz to white noise with a 5dB signal-to-noise ratio (**Figure S1a**). We chose the frequency range based on the range of dominant frequency of detected signals of *Shcizocosa* wolf spiders (480.75 ± 284.09 Hz). Also, to test the effects of the signal-to-noise ratio, we added white noise with SNRs including 1 dB, 5 dB, 10 dB, 20 dB, 30 dB, and 40 dB (**Figure S1b**). The generated noise is similar to the background noise that is typically found in our field recording files (**Figure S1a**). After adding generated background noise to the original signal, we filtered the noisy audio

files using *Noisereduce* Python package (Sainburg et al., 2020; **Figure S2**) and measured the acoustic properties using *Librosa* Python package (McFee et al., 2015). Then, to investigate the effects of background noise profiles on the acoustic properties, we conducted Friedman tests for each acoustic property with (i) frequency range and (ii) SNR of background noise as independent variables and file name as a group variable.

The simulated noise filtering suggested that adaptive noise filtering influenced the measurement of acoustic properties due to the variation in background noise profiles, but the dominant frequency is more robust to the variation in both (i) frequency range and (ii) SNR of background noise (**Table S2**). The pairwise posthoc tests showed that the effects of the frequency range of the background noise mostly came from the difference between the original audios (i.e. before adding background noise) and the filtered audios. The acoustic characters of filter audio were significantly different across the pairwise comparisons regardless of the acoustic characters, but the dominant frequency was more robust to the variation in SNR as compared to other characters.

*Verification of adaptive sound detection* -Next, we tested how robust our detection algorithm were using the adaptive amplitude threshold to different signal-to-noise ratios. For the verification of the signal detection method, we added a *Schizocosa* wolf spider signal to a random location in a 10-minute white noise with different SNRs ranging from 10 dB to 40 dB with 10 dB intervals (**Figure S3**). Then, we attempted to detect the signal again using our method. To investigate the effects of SNR on detection rate (i.e. detected signal duration/original signal duration), we conducted a linear-mixed effect regression with SNR as a predictor variable, detection rate as the response variable, and file IDs as a random effect. We used *lme4* and *emmeans* R package for the statistical analysis.

The simulated signal detection verified that the detection method with the adaptive amplitude threshold is generally robust to the variation in SNR. Across all the SNRs, our automated detection method distinguished more than 90 % of original files from the background noise (10 dB – 0.98 ± 0.07; 20 dB – 0.98 ± 0.07; 30 dB – 0.98 ± 0.07; 40 dB – 0.99 ± 0.05). The performance of the detection method was significantly varied in different SNRs (Wald $\chi^2_1$= 173.41, $P < 0.001$) mainly due to the better performance in the 40dB SNR as compared to in other SNRs (**Figure S4**).

**Table S1.** The number of collected *Schizocosa* wolf spiders from pitfall traps and detected sounds in each recording plot.

| | ***S. duplex*** | | ***S. stridulans*** | | ***S. uetzi*** | |
|---|---|---|---|---|---|---|
| | **Pitfall** | **Sounds** | **Pitfall** | **Sounds** | **Pitfall** | **Sounds** |
| **A** | - | - | 6 | 124 | 2 | 11 |
| **B** | 8 | 195 | - | - | - | - |
| **C** | - | 2 | 4 | 175 | 5 | 370 |
| **D** | 8 | 52 | - | 2 | 1 | - |
| **E** | - | - | 8 | 219 | 9 | 474 |
| **Total** | 16 | 249 | 18 | 520 | 17 | 855 |

**Table S2.** The results of (a) the Friedman test about the effects of variation in background noise profiles (frequency range & signal-to-noise ratio (SNR)) on the measurement of acoustic properties of *Schizococsa* wolf spiders' signals after noise filtering using our adaptive filtering methods and (b) posthoc comparison with Bonferonni correction. Significant differences were denoted by bold and red shades.

(a) Results of Friedman tests

| | *Dominant Frequency* | *Zero crossing rates* | *Spectral centroid* | *Spectral bandwidth* |
|---|---|---|---|---|
| **Frequency range** | ***Friedman $\chi^2_{10}$=380.35, P<0.001*** | ***Friedman $\chi^2_{10}$=18233, P<0.001*** | ***Friedman $\chi^2_{10}$=18967, P<0.001*** | ***Friedman $\chi^2_{10}$=18662, P<0.001*** |
| **SNR** | ***Friedman $\chi^2_{6}$=410.59, P<0.001*** | ***Friedman $\chi^2_{6}$=41632, P<0.001*** | ***Friedman $\chi^2_{6}$=43289, P<0.001*** | ***Friedman $\chi^2_{6}$=38300, P<0.001*** |

(b) Posthoc comparisons using Wilcoxon rank sum tests

*Dominant frequency*

| **Frequency range (Hz)** | **0-100** | **100-200** | **200-300** | **300-400** | **400-500** | **500-600** | **600-700** | **700-800** | **800-900** | **900-1000** |
|---|---|---|---|---|---|---|---|---|---|---|
| **0-100** | | | | | | | | | | |
| **100-200** | *P*=1.000 | | | | | | | | | |
| **200-300** | *P*=1.000 | *P*=1.000 | | | | | | | | |
| **300-400** | *P*=0.406 | *P*=1.000 | *P*=1.000 | | | | | | | |
| **400-500** | *P*=1.000 | *P*=1.000 | *P*=1.000 | *P*=1.000 | | | | | | |
| **500-600** | *P*=1.000 | *P*=1.000 | *P*=1.000 | *P*=1.000 | *P*=1.000 | | | | | |
| **600-700** | *P*=1.000 | *P*=1.000 | *P*=1.000 | *P*=1.000 | *P*=1.000 | *P*=1.000 | | | | |
| **700-800** | *P*=1.000 | *P*=1.000 | *P*=1.000 | *P*=1.000 | *P*=1.000 | *P*=1.000 | *P*=1.000 | | | |
| **800-900** | *P*=1.000 | *P*=1.000 | *P*=1.000 | *P*=1.000 | *P*=1.000 | *P*=1.000 | *P*=1.000 | *P*=1.000 | | |
| **900-1000** | *P*=1.000 | *P*=1.000 | *P*=1.000 | *P*=1.000 | *P*=1.000 | *P*=1.000 | *P*=1.000 | *P*=1.000 | *P*=1.000 | |
| **Original** | ***P* < 0.001** | ***P*=0.014** | ***P* < 0.001** | *P*=0.434 | ***P*=0.009** | ***P* < 0.001** | ***P* < 0.001** | ***P*=0.002** | ***P* < 0.001** | ***P*=0.002** |

| **SNR (dB)** | **1** | **5** | **10** | **20** | **30** | **40** |
|---|---|---|---|---|---|---|
| **1** | | | | | | |
| **5** | ***P*<0.001** | | | | | |
| **10** | ***P*<0.001** | *P*=1.000 | | | | |
| **20** | ***P*<0.001** | *P*=1.000 | *P*=0.157 | | | |
| **30** | ***P*=0.015** | *P*=0.069 | ***P*<0.001** | *P*=0.450 | | |
| **40** | *P*=1.000 | ***P*<0.001** | ***P*<0.001** | ***P*<0.001** | *P*=0.078 | |
| **Original** | *P*=1.000 | ***P*<0.001** | ***P*<0.001** | ***P*<0.001** | ***P*<0.001** | *P*=1.000 |

*Zero-crossing rate*

| **Frequency range (Hz)** | **0-100** | **100-200** | **200-300** | **300-400** | **400-500** | **500-600** | **600-700** | **700-800** | **800-900** | **900-1000** |
|---|---|---|---|---|---|---|---|---|---|---|
| **0-100** | | | | | | | | | | |
| **100-200** | *P*=1.000 | | | | | | | | | |
| **200-300** | *P*=1.000 | *P*=1.000 | | | | | | | | |

| | | | | | | | | | | |
|---|---|---|---|---|---|---|---|---|---|---|
| **300-400** | *P*=1.000 | *P*=1.000 | *P*=1.000 | | | | | | | |
| **400-500** | *P*=0.848 | *P*=1.000 | *P*=1.000 | *P*=1.000 | | | | | | |
| **500-600** | *P*=0.134 | *P*=1.000 | *P*=1.000 | *P*=1.000 | *P*=1.000 | | | | | |
| **600-700** | *P*=0.237 | *P*=1.000 | *P*=1.000 | *P*=1.000 | *P*=1.000 | *P*=1.000 | | | | |
| **700-800** | *P*=0.206 | *P*=1.000 | *P*=1.000 | *P*=1.000 | *P*=1.000 | *P*=1.000 | *P*=1.000 | | | |
| **800-900** | *P*=0.131 | *P*=1.000 | *P*=1.000 | *P*=1.000 | *P*=1.000 | *P*=1.000 | *P*=1.000 | *P*=1.000 | | |
| **900-1000** | *P*=0.231 | *P*=1.000 | *P*=1.000 | *P*=1.000 | *P*=1.000 | *P*=1.000 | *P*=1.000 | *P*=1.000 | *P*=1.000 | |
| **Original** | ***P* < 0.001** | ***P* < 0.001** | ***P* < 0.001** | ***P* < 0.001** | ***P* < 0.001** | ***P* < 0.001** | ***P* < 0.001** | ***P* < 0.001** | ***P* < 0.001** | ***P* < 0.001** |

| SNR (dB) | 1 | 5 | 10 | 20 | 30 | 40 |
|---|---|---|---|---|---|---|
| **1** | | | | | | |
| **5** | ***P*<0.001** | | | | | |
| **10** | ***P*<0.001** | ***P*<0.001** | | | | |
| **20** | ***P*<0.001** | ***P*<0.001** | ***P*<0.001** | | | |
| **30** | ***P*<0.001** | ***P*<0.001** | ***P*<0.001** | ***P*<0.001** | | |
| **40** | ***P*<0.001** | ***P*<0.001** | ***P*<0.001** | ***P*<0.001** | ***P*=0.012** | |
| **Original** | ***P*<0.001** | ***P*<0.001** | ***P*<0.001** | ***P*<0.001** | ***P*<0.001** | ***P*<0.001** |

*Spectral centroid*

| Frequency range (Hz) | 0-100 | 100-200 | 200-300 | 300-400 | 400-500 | 500-600 | 600-700 | 700-800 | 800-900 | 900-1000 |
|---|---|---|---|---|---|---|---|---|---|---|
| **0-100** | | | | | | | | | | |
| **100-200** | *P*=1.000 | | | | | | | | | |
| **200-300** | *P*=1.000 | *P*=1.000 | | | | | | | | |
| **300-400** | *P*=1.000 | *P*=1.000 | *P*=1.000 | | | | | | | |
| **400-500** | *P*=0.848 | *P*=1.000 | *P*=1.000 | *P*=1.000 | | | | | | |
| **500-600** | *P*=0.134 | *P*=1.000 | *P*=1.000 | *P*=1.000 | *P*=1.000 | | | | | |
| **600-700** | *P*=0.237 | *P*=1.000 | *P*=1.000 | *P*=1.000 | *P*=1.000 | *P*=1.000 | | | | |
| **700-800** | *P*=0.206 | *P*=1.000 | *P*=1.000 | *P*=1.000 | *P*=1.000 | *P*=1.000 | *P*=1.000 | | | |
| **800-900** | *P*=0.131 | *P*=1.000 | *P*=1.000 | *P*=1.000 | *P*=1.000 | *P*=1.000 | *P*=1.000 | *P*=1.000 | | |
| **900-1000** | *P*=0.231 | *P*=1.000 | *P*=1.000 | *P*=1.000 | *P*=1.000 | *P*=1.000 | *P*=1.000 | *P*=1.000 | *P*=1.000 | |
| **Original** | ***P* < 0.001** | ***P* < 0.001** | ***P* < 0.001** | ***P* < 0.001** | ***P* < 0.001** | ***P* < 0.001** | ***P* < 0.001** | ***P* < 0.001** | ***P* < 0.001** | ***P* < 0.001** |

| SNR (dB) | 1 | 5 | 10 | 20 | 30 | 40 |
|---|---|---|---|---|---|---|
| **1** | | | | | | |
| **5** | ***P*<0.001** | | | | | |
| **10** | ***P*<0.001** | ***P*<0.001** | | | | |
| **20** | ***P*<0.001** | ***P*<0.001** | ***P*<0.001** | | | |
| **30** | ***P*<0.001** | ***P*<0.001** | ***P*<0.001** | ***P*<0.001** | | |
| **40** | ***P*<0.001** | ***P*<0.001** | ***P*<0.001** | ***P*<0.001** | ***P*<0.001** | |
| **Original** | ***P*<0.001** | ***P*<0.001** | ***P*<0.001** | ***P*<0.001** | ***P*<0.001** | *P*=0.35 |

*Spectral bandwidth*

| Frequency range (Hz) | 0-100 | 100-200 | 200-300 | 300-400 | 400-500 | 500-600 | 600-700 | 700-800 | 800-900 | 900-1000 |
|---|---|---|---|---|---|---|---|---|---|---|
| **0-100** | | | | | | | | | | |
| **100-200** | *P*=1.000 | | | | | | | | | |
| **200-300** | *P*=1.000 | *P*=1.000 | | | | | | | | |
| **300-400** | *P*=1.000 | *P*=1.000 | *P*=1.000 | | | | | | | |
| **400-500** | *P*=0.848 | *P*=1.000 | *P*=1.000 | *P*=1.000 | | | | | | |
| **500-600** | *P*=0.134 | *P*=1.000 | *P*=1.000 | *P*=1.000 | *P*=1.000 | | | | | |
| **600-700** | *P*=0.237 | *P*=1.000 | *P*=1.000 | *P*=1.000 | *P*=1.000 | *P*=1.000 | | | | |
| **700-800** | *P*=0.206 | *P*=1.000 | *P*=1.000 | *P*=1.000 | *P*=1.000 | *P*=1.000 | *P*=1.000 | | | |

| | | | | | | | | | | |
|---|---|---|---|---|---|---|---|---|---|---|
| **800-900** | *P*=0.131 | *P*=1.000 | *P*=1.000 | *P*=1.000 | *P*=1.000 | *P*=1.000 | *P*=1.000 | *P*=1.000 | | |
| **900-1000** | *P*=0.231 | *P*=1.000 | *P*=1.000 | *P*=1.000 | *P*=1.000 | *P*=1.000 | *P*=1.000 | *P*=1.000 | *P*=1.000 | |
| **Original** | ***P* < 0.001** | ***P* < 0.001** | ***P* < 0.001** | ***P* < 0.001** | ***P* < 0.001** | ***P* < 0.001** | ***P* < 0.001** | ***P* < 0.001** | ***P* < 0.001** | ***P* < 0.001** |

| **SNR (dB)** | **1** | **5** | **10** | **20** | **30** | **40** |
|---|---|---|---|---|---|---|
| **1** | | | | | | |
| **5** | *P*=1.000 | | | | | |
| **10** | ***P*<0.001** | ***P*<0.001** | | | | |
| **20** | ***P*<0.001** | ***P*<0.001** | ***P*<0.001** | | | |
| **30** | ***P*<0.001** | ***P*<0.001** | ***P*<0.001** | ***P*<0.001** | | |
| **40** | ***P*<0.001** | ***P*<0.001** | ***P*<0.001** | ***P*<0.001** | ***P*<0.001** | |
| **Original** | ***P*<0.001** | ***P*<0.001** | ***P*<0.001** | ***P*<0.001** | ***P*<0.001** | ***P*<0.001** |

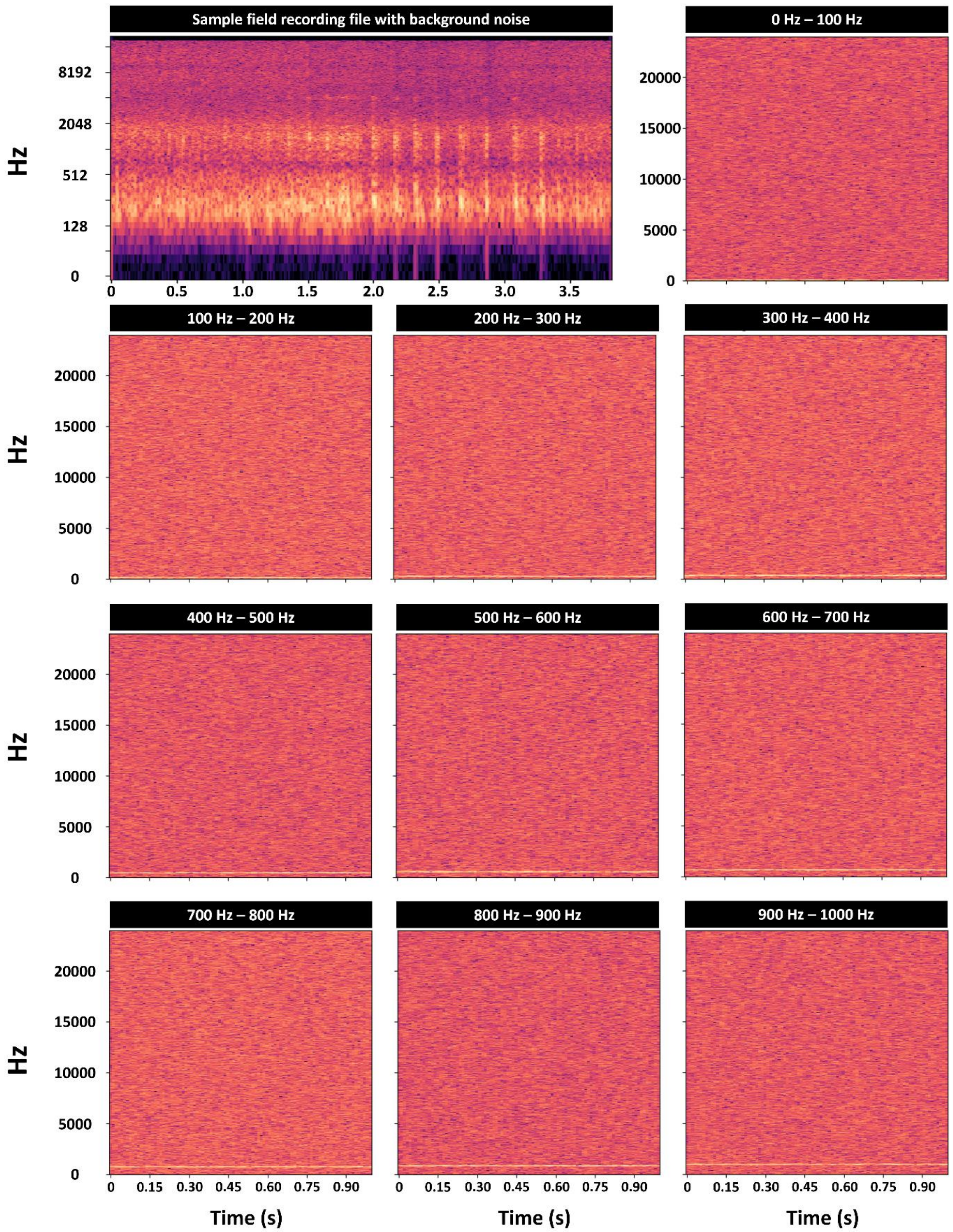


**Figure S1.** An example of background noise in the field recording and generated noise for the verification of the filtering method.

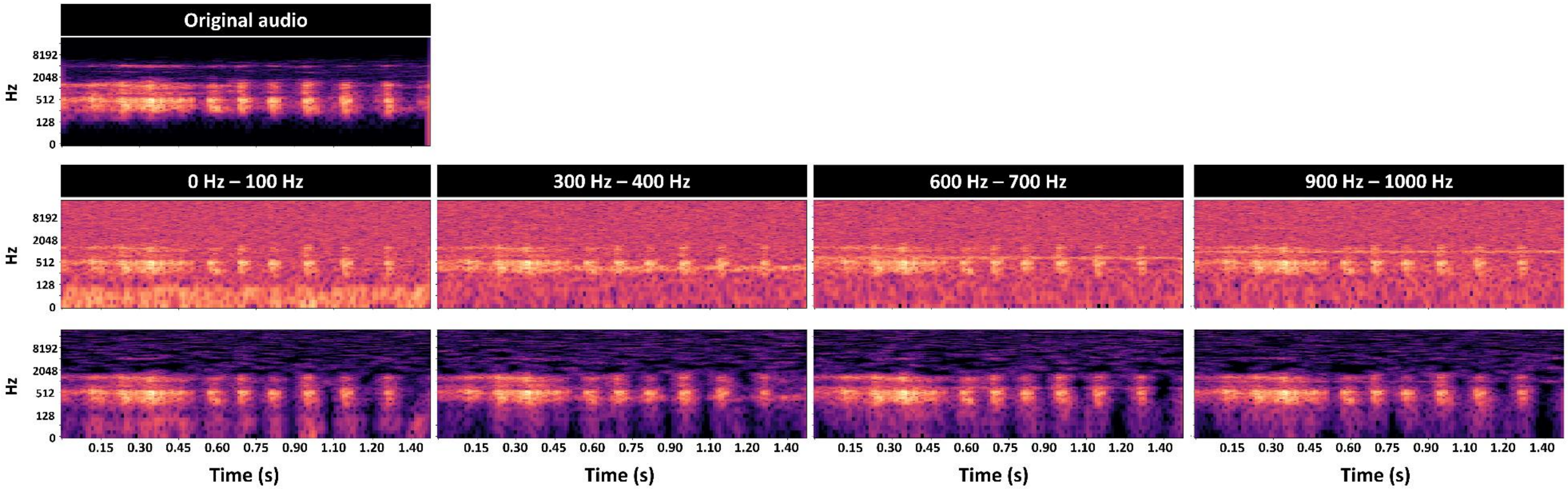


**Figure S2.** An example of before (2nd row) and after (3rd row) the adaptive noise filtering of the derivatives of the original audio file with different background noise profiles. We only presented the subset of derivatives for visualization purposes.

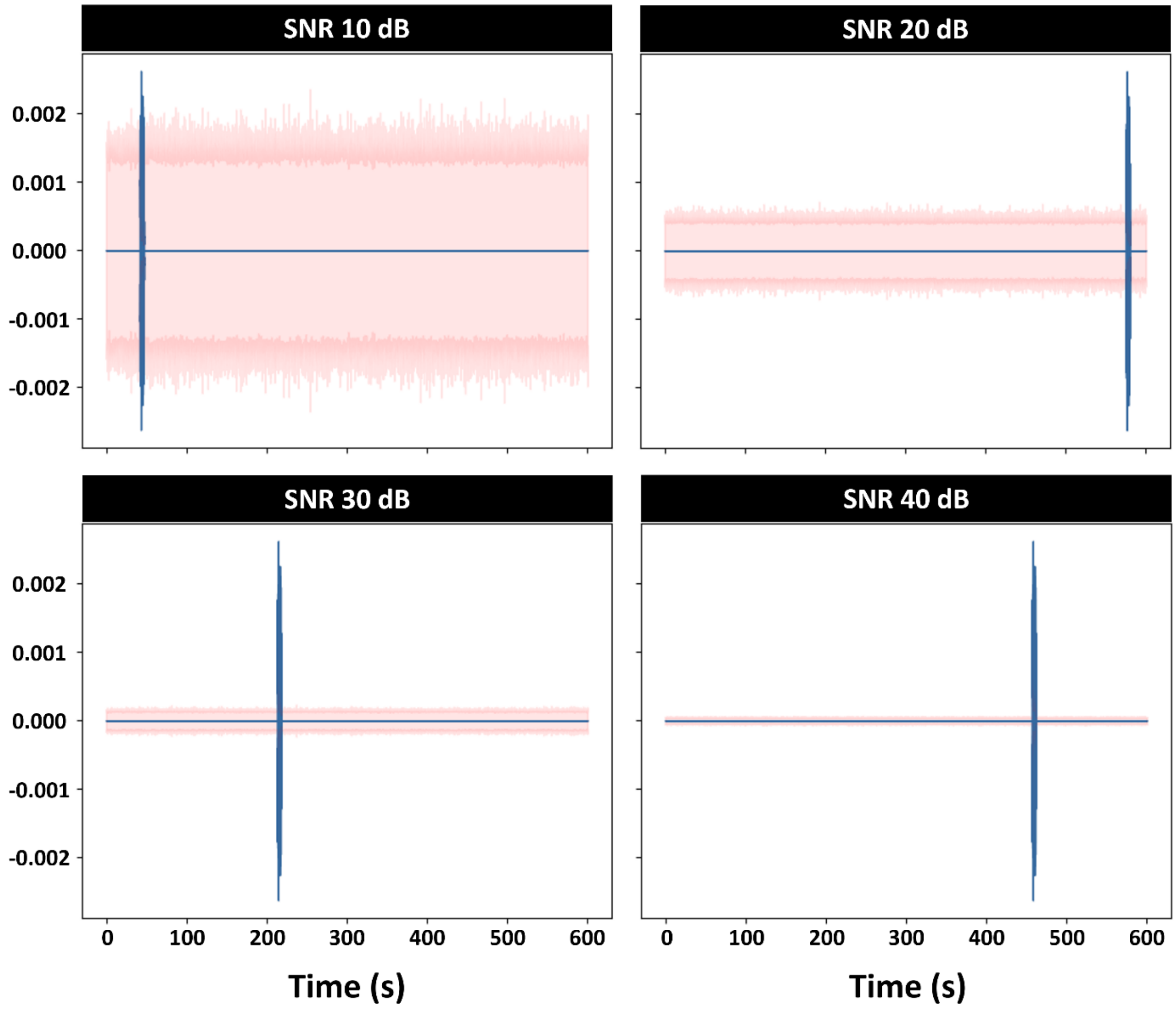


**Figure S3.** Examples of audio with different signal-to-noise ratios for the verification of methods.

(a)

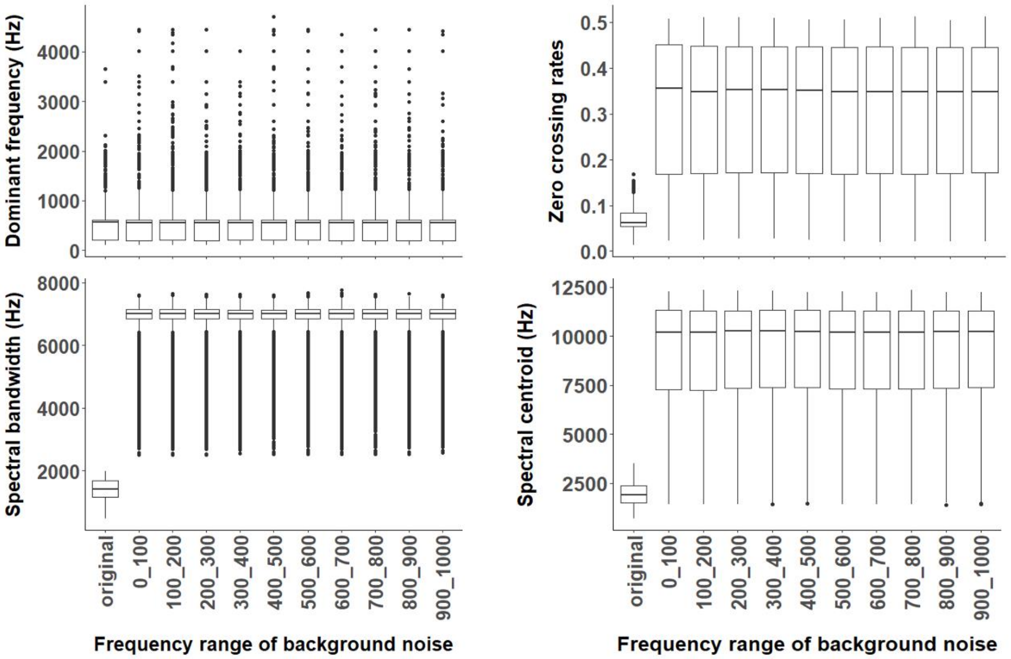


(b)

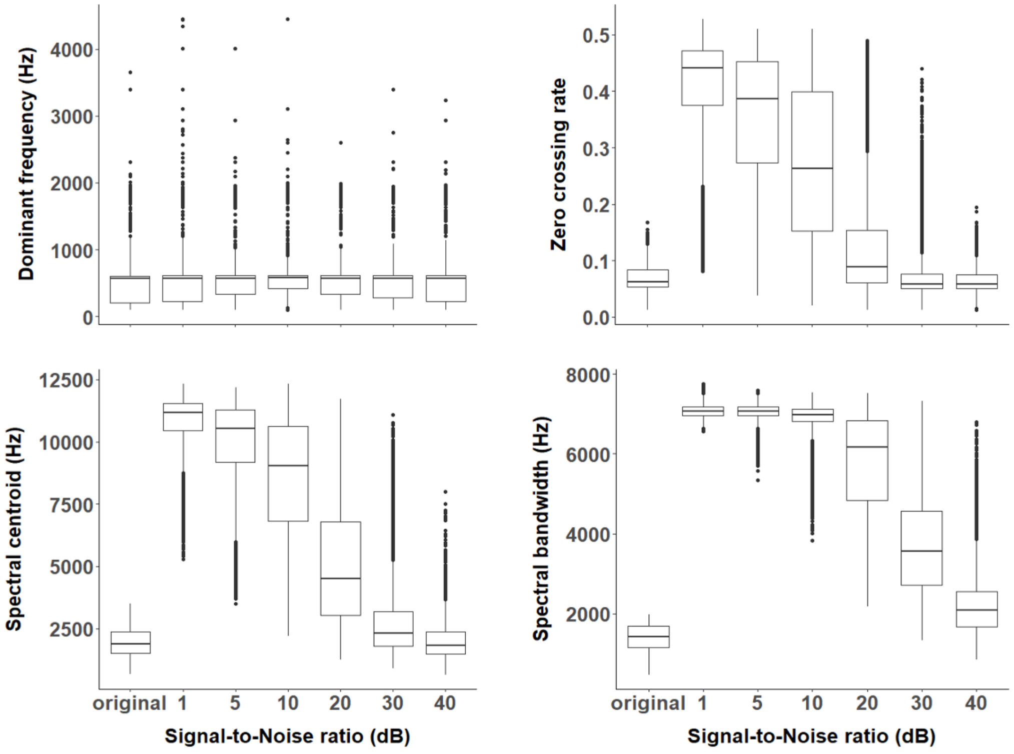


**Figure S4.** The box plots of acoustic properties after (a) adaptive filtering of different background noise profiles and (b) signal-to-noise ratios.

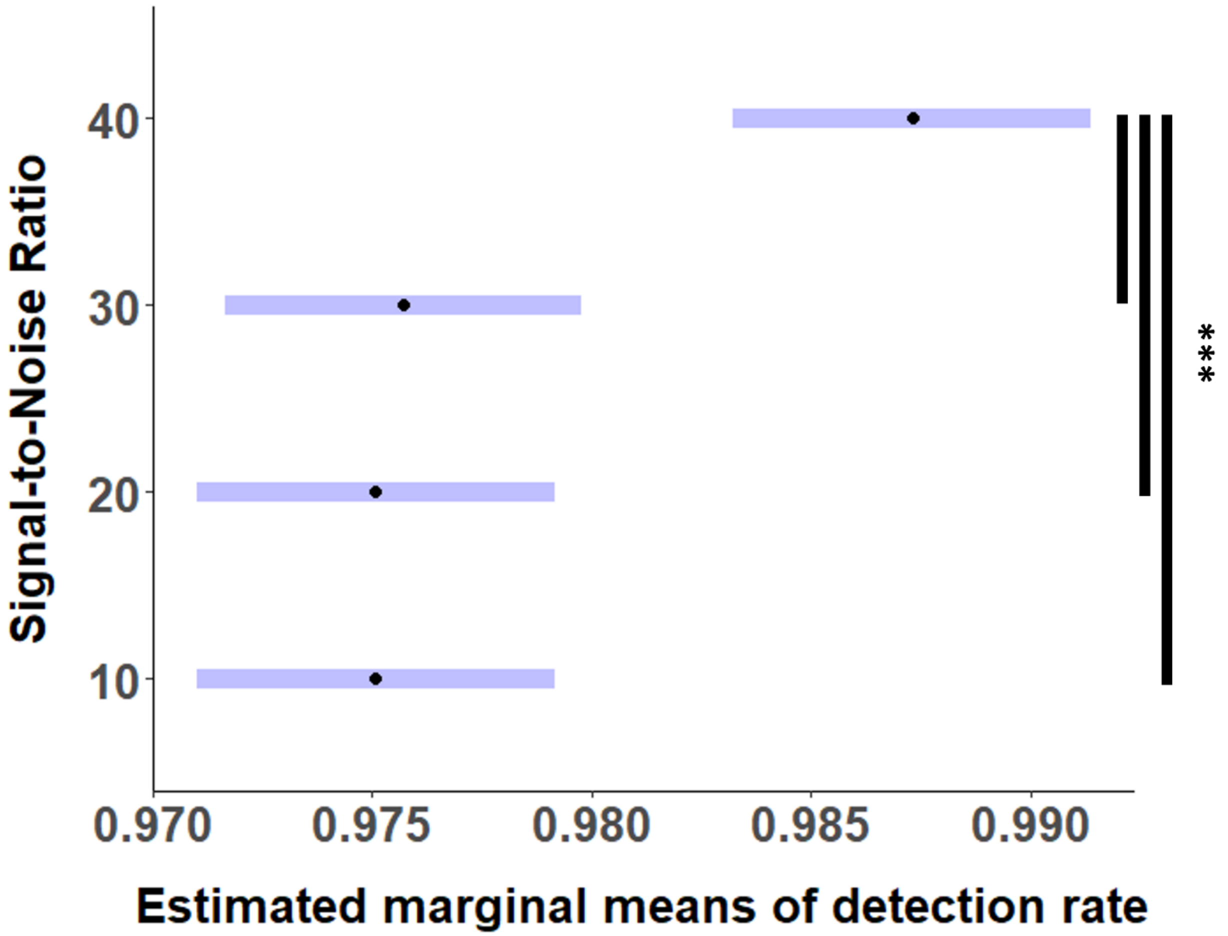


**Figure S5.** Estimated marginal means of detection rate across different signal-to-noise ratios (SNR). The estimated marginal means were displayed by dots with bars presenting a 95% confidence interval. The significant pairwise difference was denoted by asterisks (* <0.05, ** < 0.005, *** < 0.001). The P-value of pairwise comparison was adjusted by Bonferroni correction.

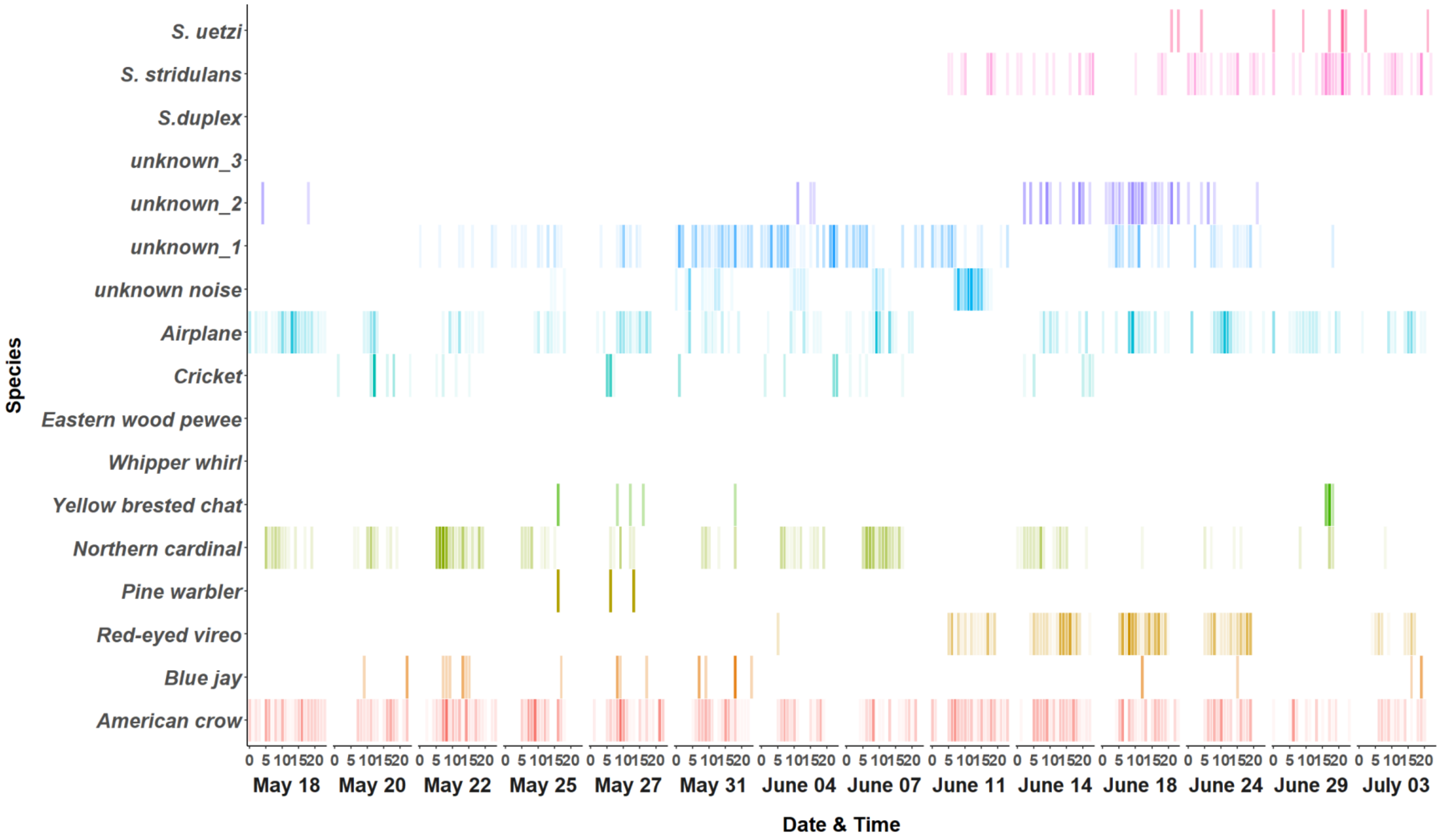


**Figure S6.** The temporal variation in sound/vibration detection of different species in the recording plot A

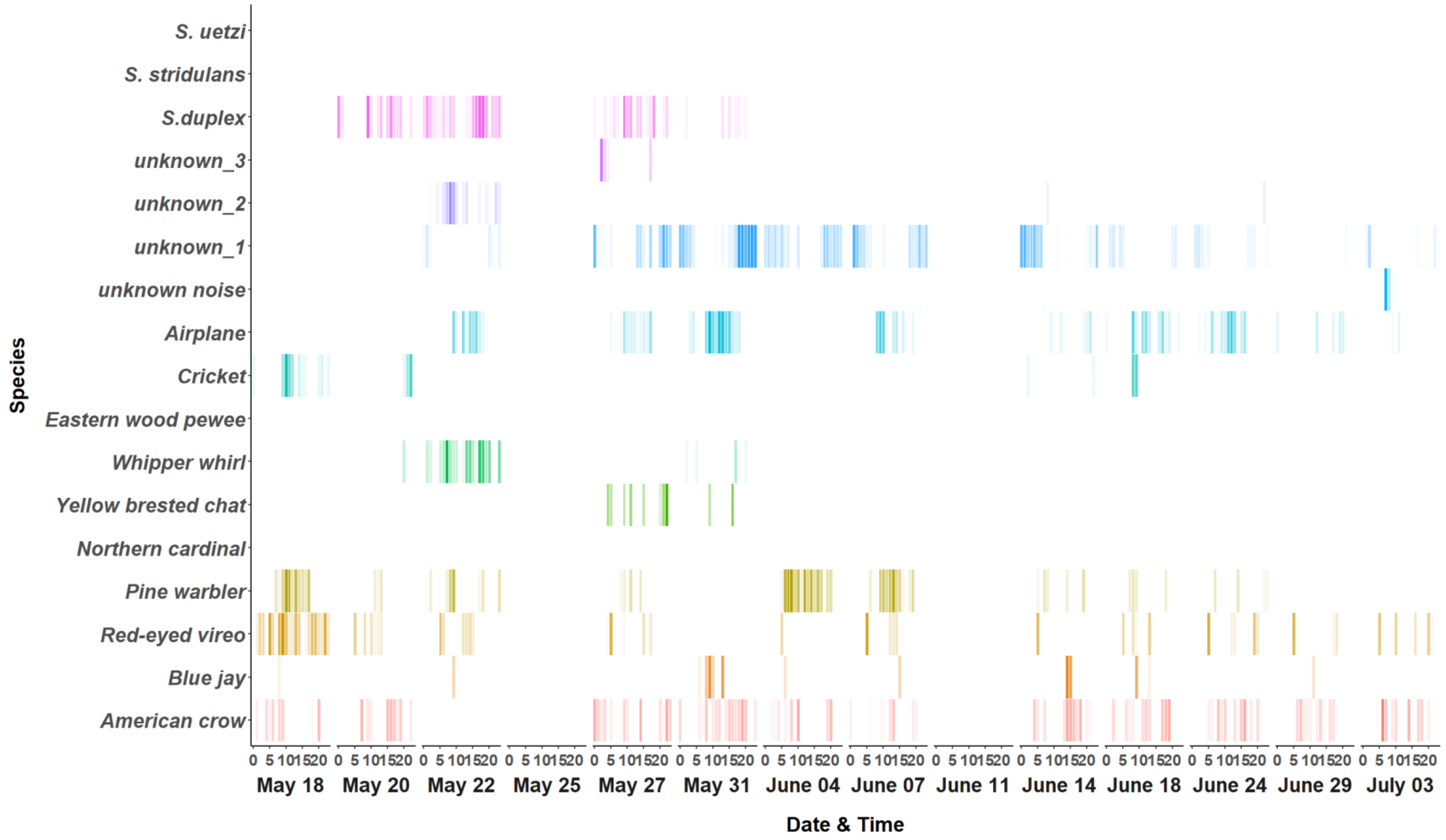


**Figure S7.** The temporal variation in sound/vibration detection of different species in the recording plot B

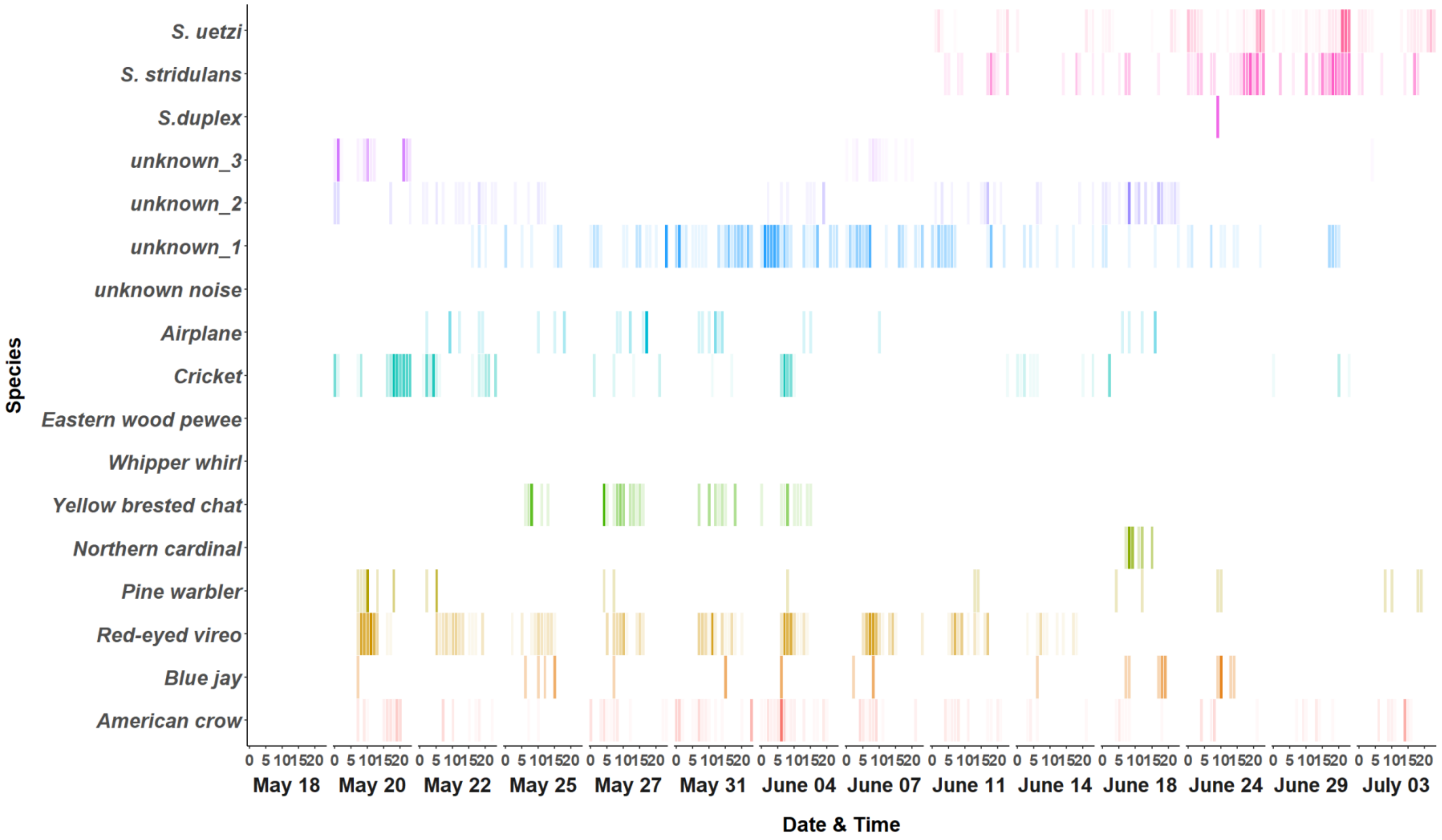


**Figure S8.** The temporal variation in sound/vibration detection of different species in the recording plot C.

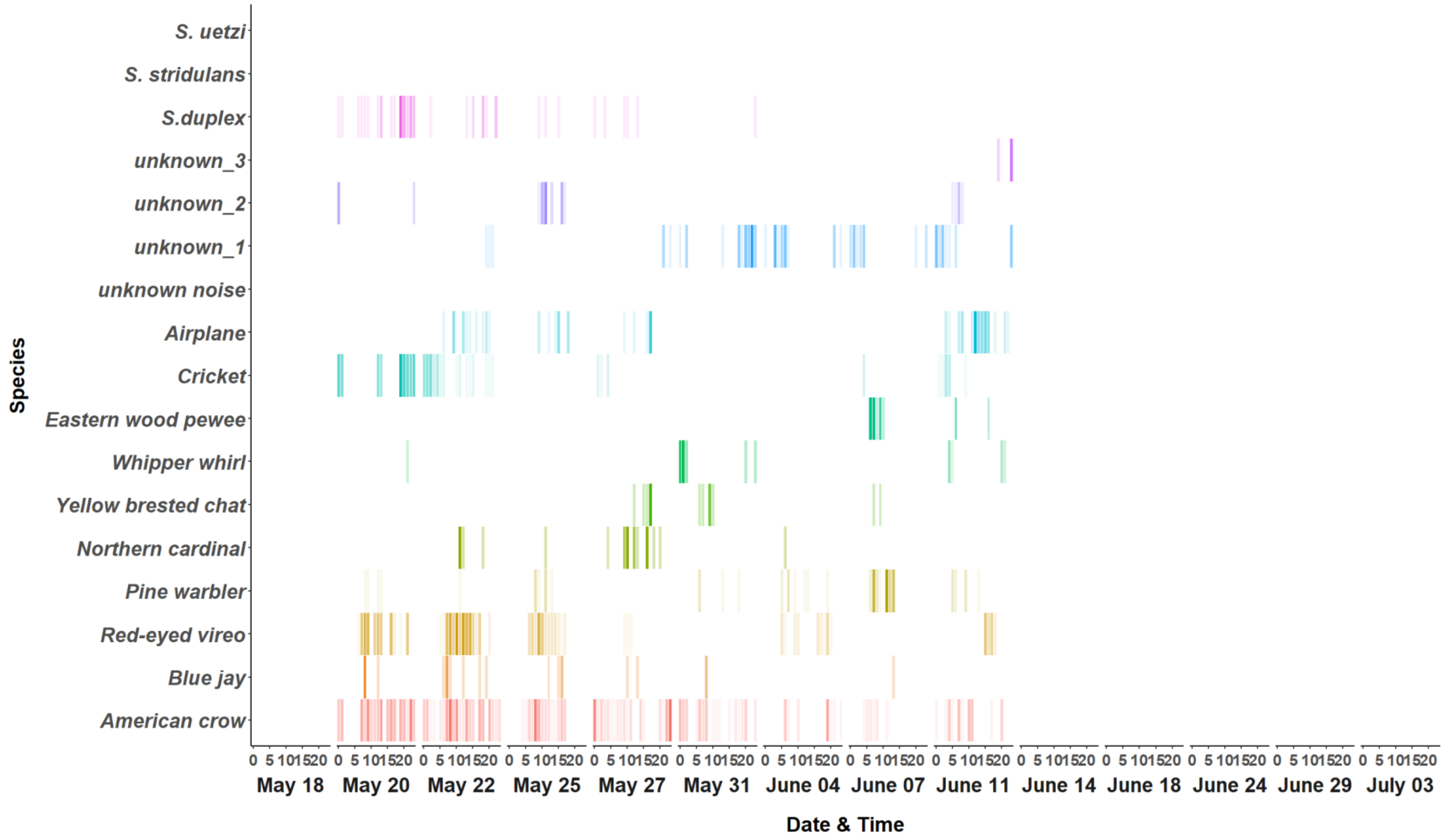


**Figure S9.** The temporal variation in sound/vibration detection of different species in recording plot D. The recording in plot D was stopped on June 11 because of the damage on the contact microphone array by wild animals.

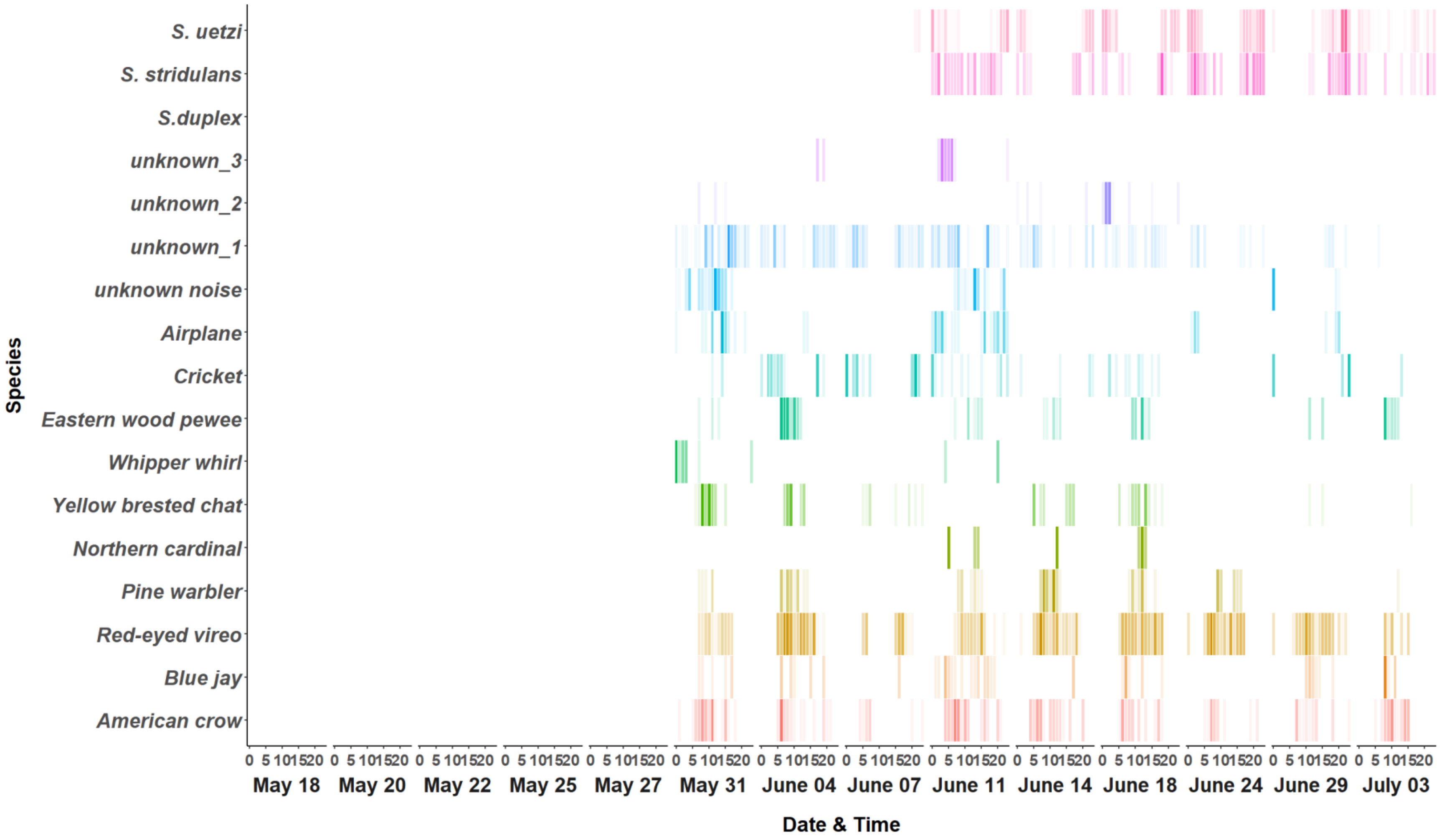


**Figure S10.** The temporal variation in sound/vibration detection of different species in the recording plot E. The recording in plot E was started on May 31st due to the delayed setting of the contact microphone array.
