# Supplementary material for: Vibroscape analysis reveals acoustic niche overlap and plastic alteration of vibratory courtship signals in ground-dwelling wolf spiders
Source: Commun Biol. 2024 Jan 5;7:23. doi: 10.1038/s42003-023-05700-6 (PMC10770364; doi:10.1038/s42003-023-05700-6)
Supplement: Supplementary file 3 — Description of Supplementary Materials [file 42003_2023_5700_MOESM3_ESM.docx]

**Description of Additional Supplementary Files**

**File name:** Supplementary Data 1

**Description:** Detailed explanation of noise filtering/sound detection methods with sample audio which cannot be included in the pdf format of the Supplementary Material S1

**File name:** Supplementary Software

**Description:** Python codes of noise filtering/sound detection programs

**File name:** Supplementary Audio

**Description:** Audio files of unclassified substrate-borne vibrations
