# Supplementary material for: Vibroscape analysis reveals acoustic niche overlap and plastic alteration of vibratory courtship signals in ground-dwelling wolf spiders
Source: Commun Biol. 2024 Jan 5;7:23. doi: 10.1038/s42003-023-05700-6 (PMC10770364; doi:10.1038/s42003-023-05700-6)
Supplement: Supplementary file 4 — Supplementary Data 1 [file 42003_2023_5700_MOESM4_ESM.pptx]

## Slide 1
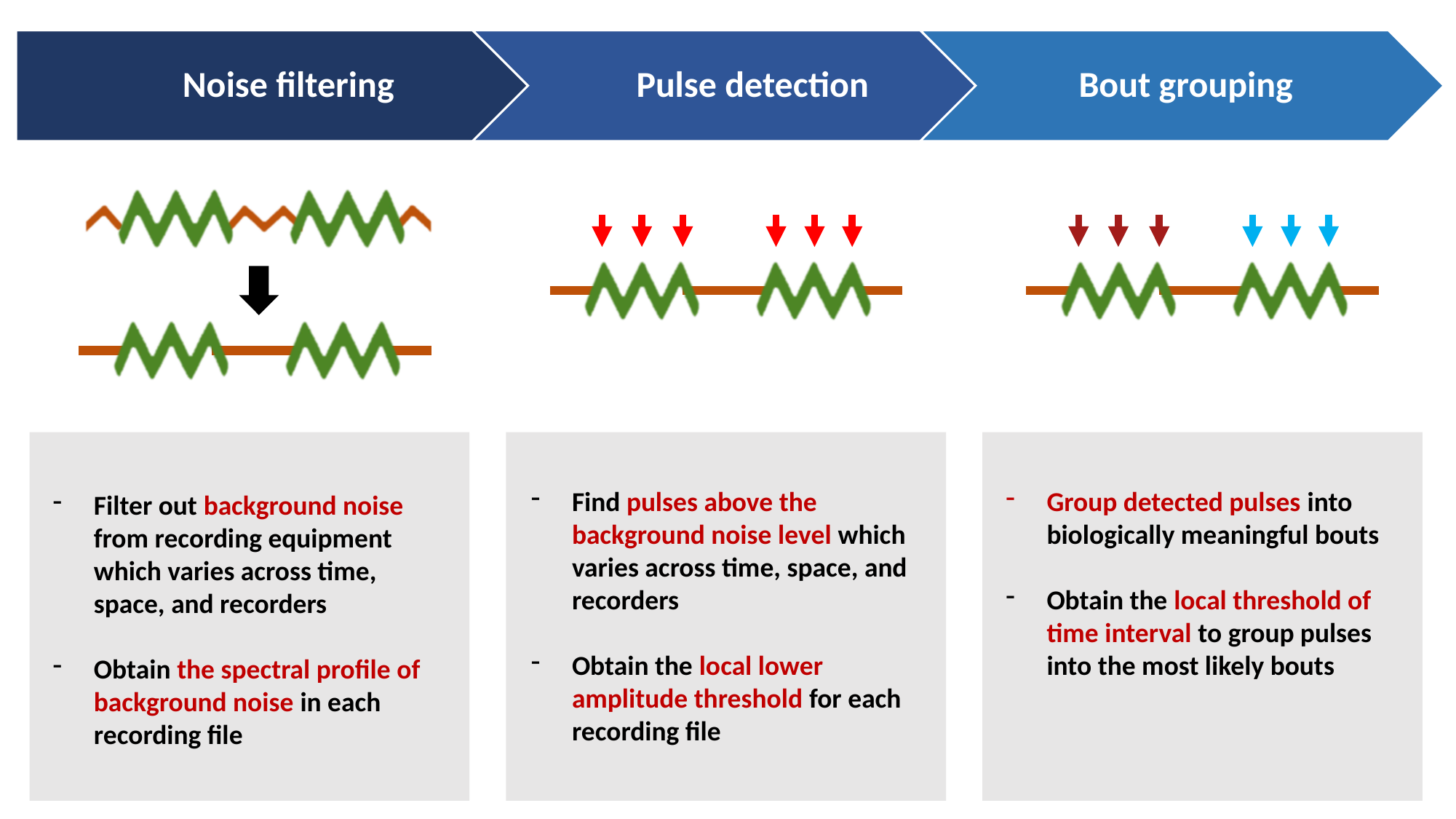

Find pulses above the background noise level which varies across time, space, and recorders
Obtain the local lower amplitude threshold for each recording file
Group detected pulses into biologically meaningful bouts
Obtain the local threshold of time interval to group pulses into the most likely bouts
Filter out background noise from recording equipment which varies across time, space, and recorders
Obtain the spectral profile of background noise in each recording file

## Slide 2
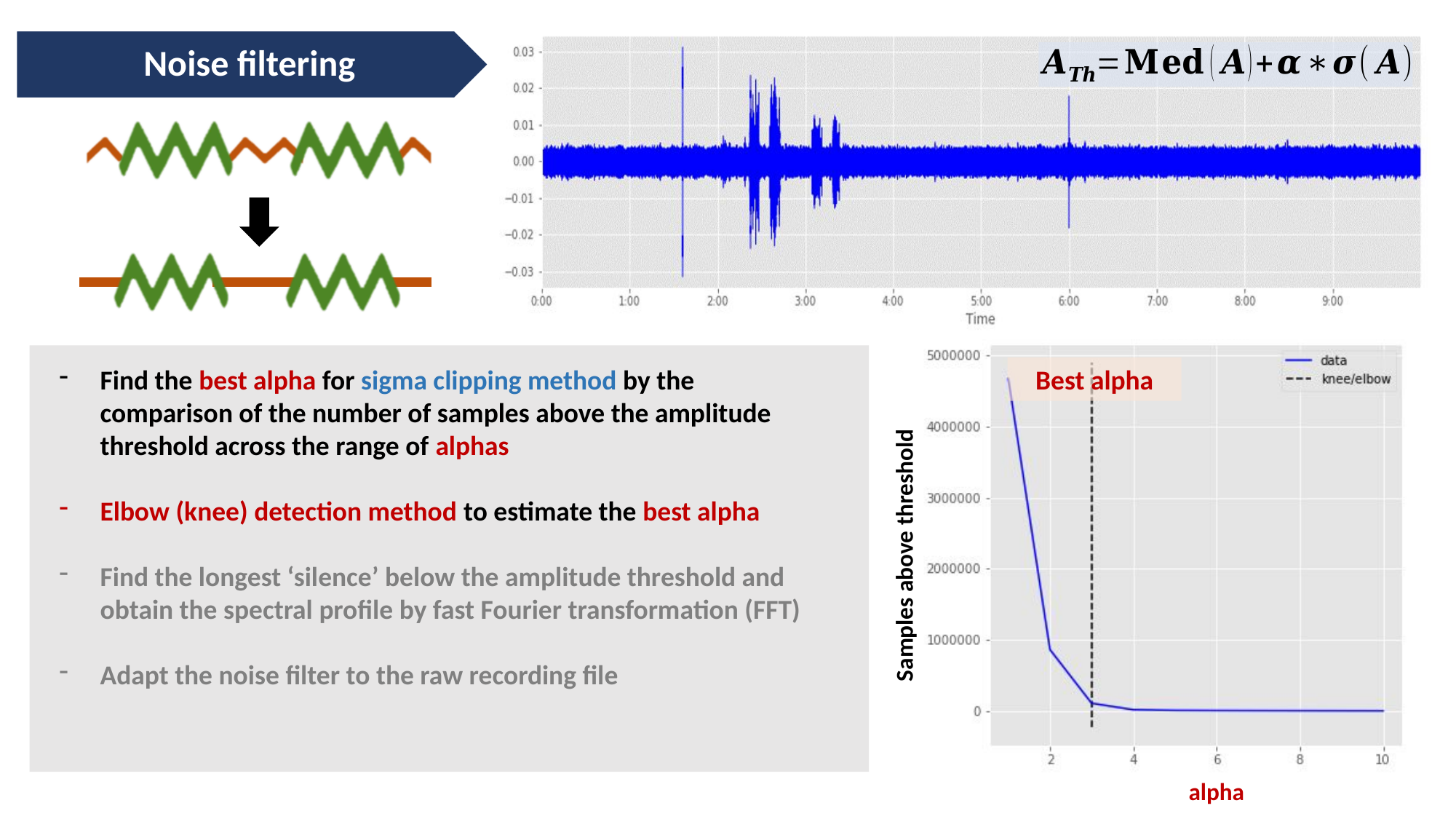

Samples above threshold
alpha
Best alpha
Find the best alpha for sigma clipping method by the comparison of the number of samples above the amplitude threshold across the range of alphas
Elbow (knee) detection method to estimate the best alpha
Find the longest ‘silence’ below the amplitude threshold and obtain the spectral profile by fast Fourier transformation (FFT)
Adapt the noise filter to the raw recording file

## Slide 3
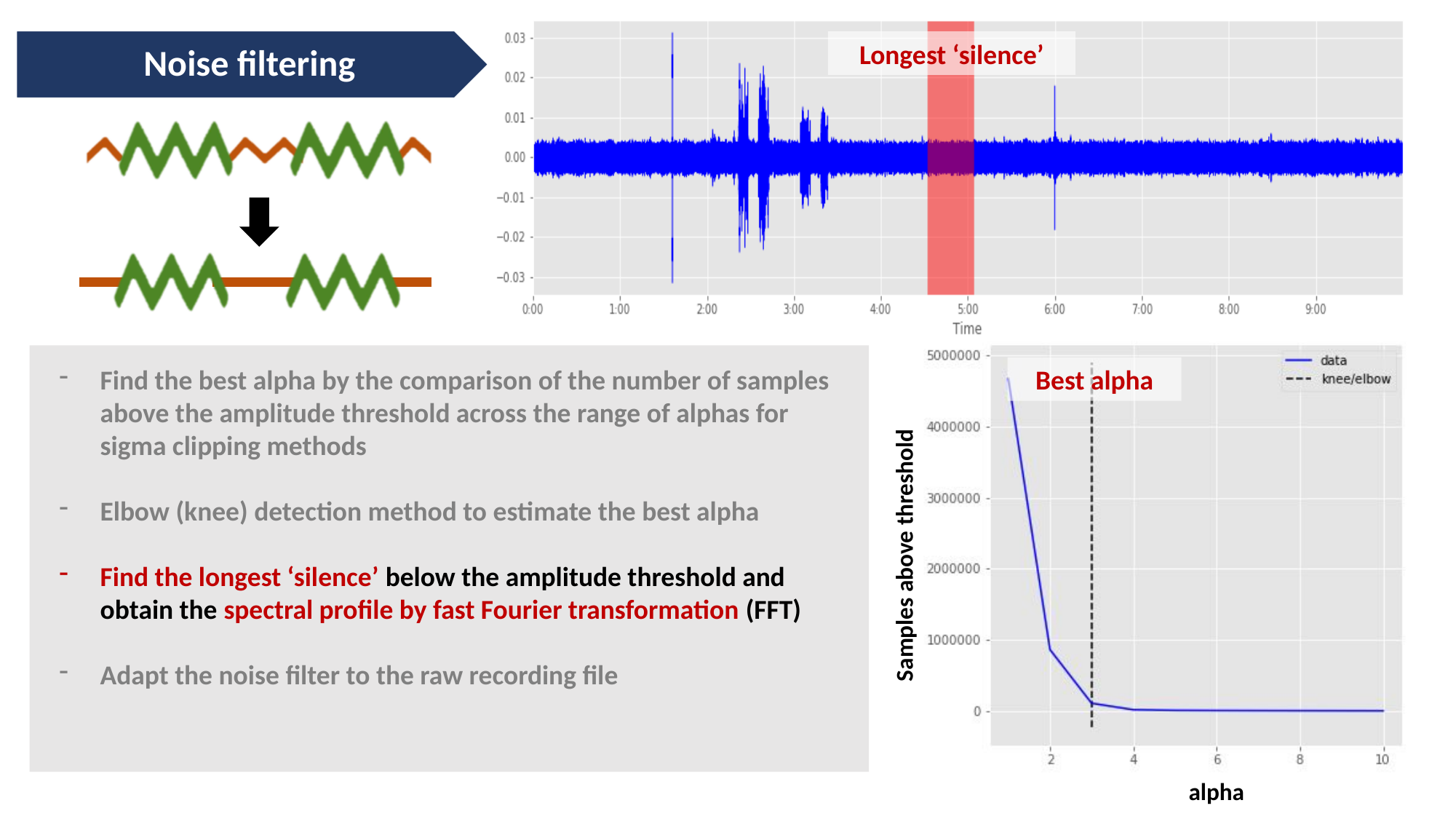

Longest ‘silence’
Samples above threshold
alpha
Best alpha
Find the best alpha by the comparison of the number of samples above the amplitude threshold across the range of alphas for sigma clipping methods
Elbow (knee) detection method to estimate the best alpha
Find the longest ‘silence’ below the amplitude threshold and obtain the spectral profile by fast Fourier transformation (FFT)
Adapt the noise filter to the raw recording file

## Slide 4
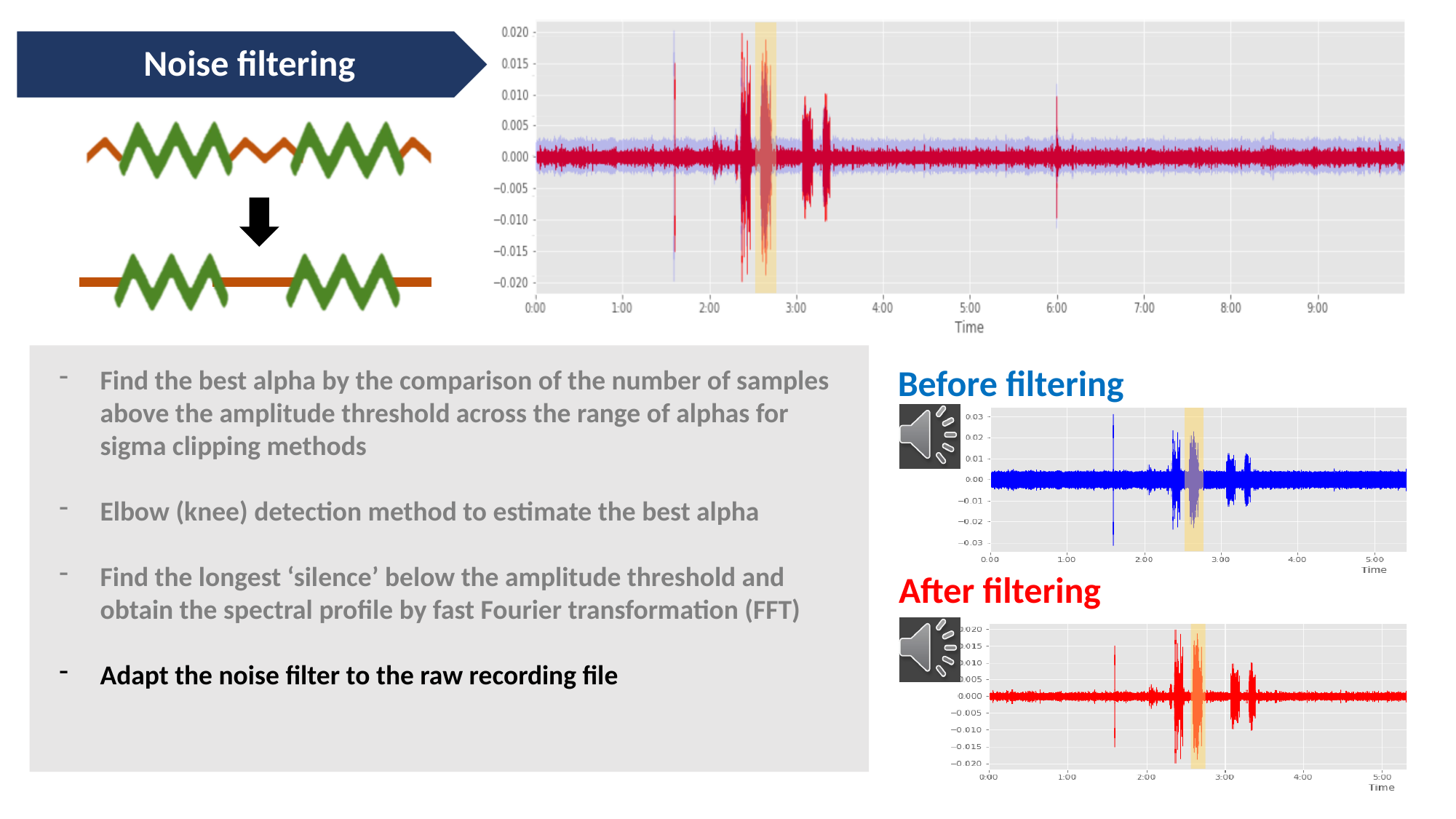

Before filtering
Find the best alpha by the comparison of the number of samples above the amplitude threshold across the range of alphas for sigma clipping methods
Elbow (knee) detection method to estimate the best alpha
Find the longest ‘silence’ below the amplitude threshold and obtain the spectral profile by fast Fourier transformation (FFT)
Adapt the noise filter to the raw recording file
After filtering

## Slide 5
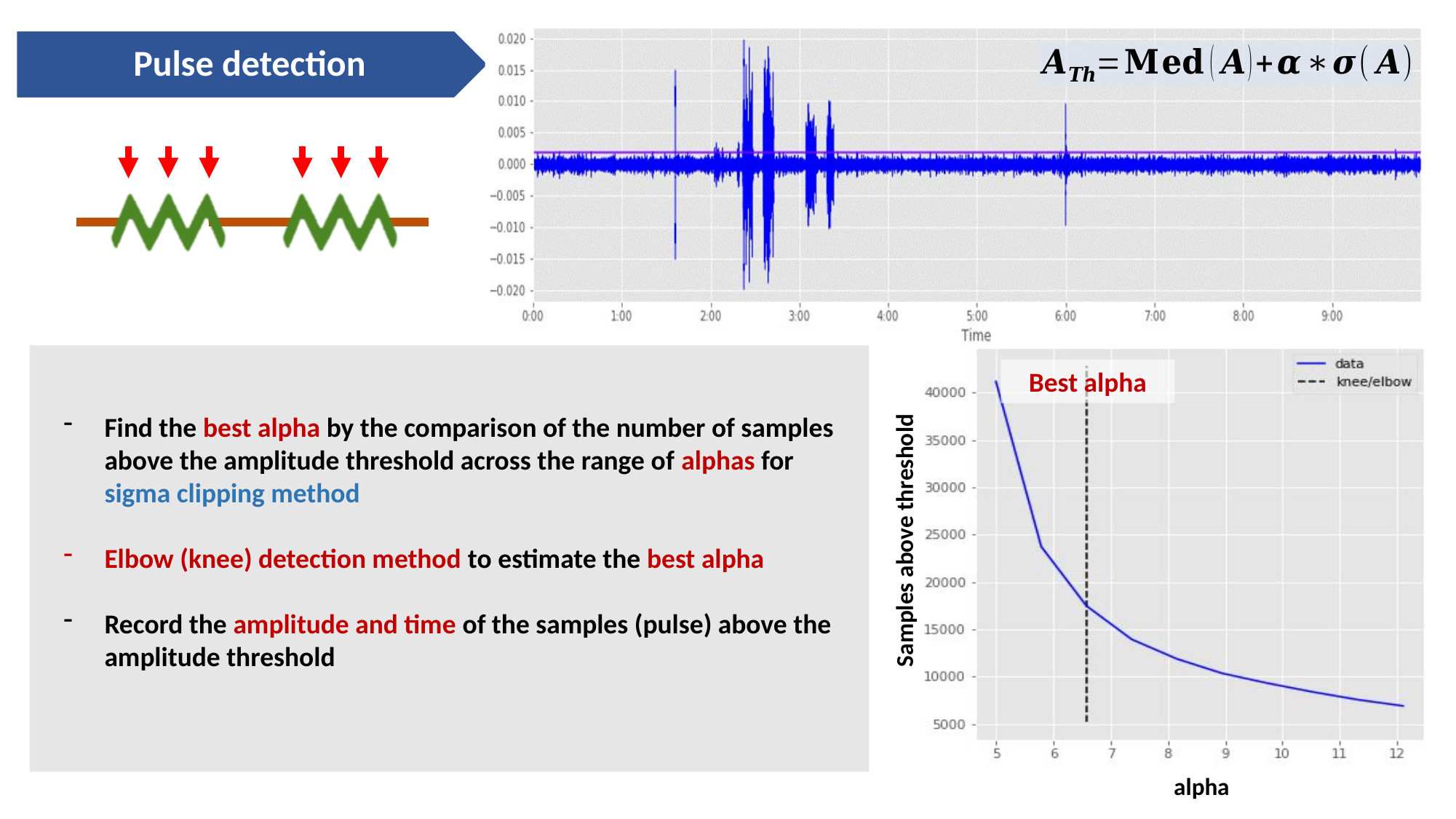

Best alpha
Samples above threshold
alpha
Find the best alpha by the comparison of the number of samples above the amplitude threshold across the range of alphas for sigma clipping method
Elbow (knee) detection method to estimate the best alpha
Record the amplitude and time of the samples (pulse) above the amplitude threshold

## Slide 6
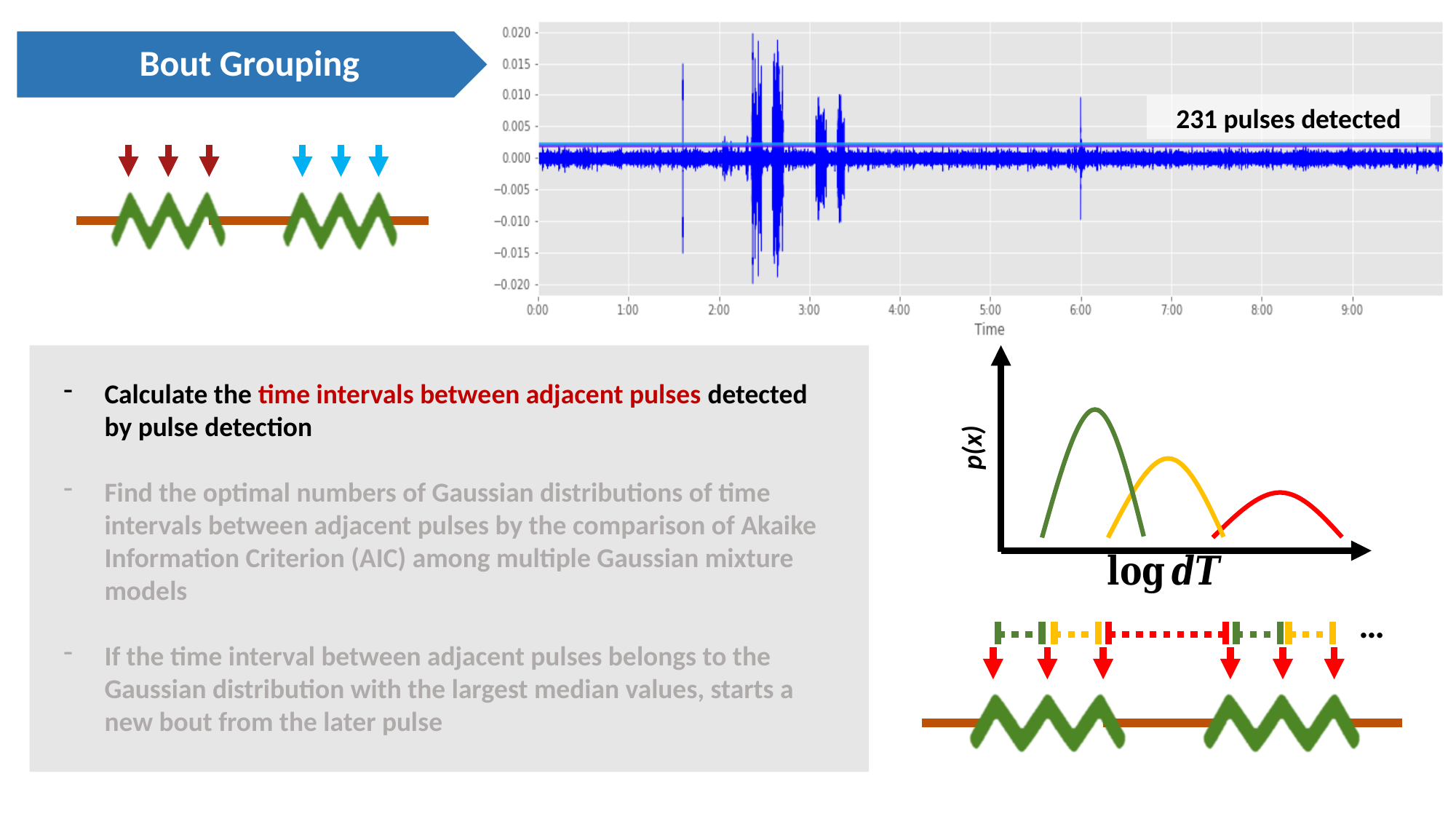

231 pulses detected
p(x)
Calculate the time intervals between adjacent pulses detected by pulse detection
Find the optimal numbers of Gaussian distributions of time intervals between adjacent pulses by the comparison of Akaike Information Criterion (AIC) among multiple Gaussian mixture models
If the time interval between adjacent pulses belongs to the Gaussian distribution with the largest median values, starts a new bout from the later pulse
…

## Slide 7
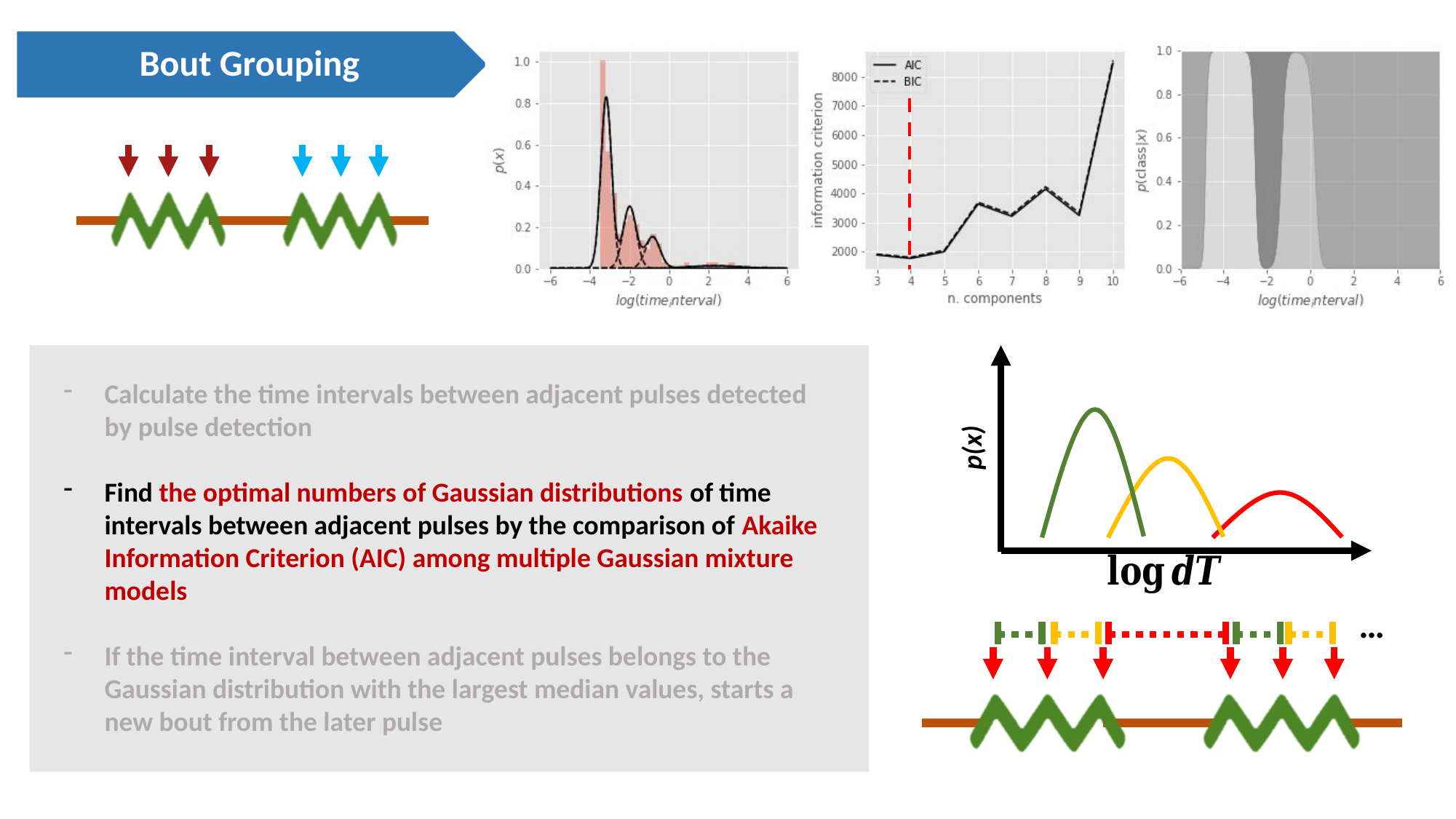

p(x)
Calculate the time intervals between adjacent pulses detected by pulse detection
Find the optimal numbers of Gaussian distributions of time intervals between adjacent pulses by the comparison of Akaike Information Criterion (AIC) among multiple Gaussian mixture models
If the time interval between adjacent pulses belongs to the Gaussian distribution with the largest median values, starts a new bout from the later pulse
…

## Slide 8
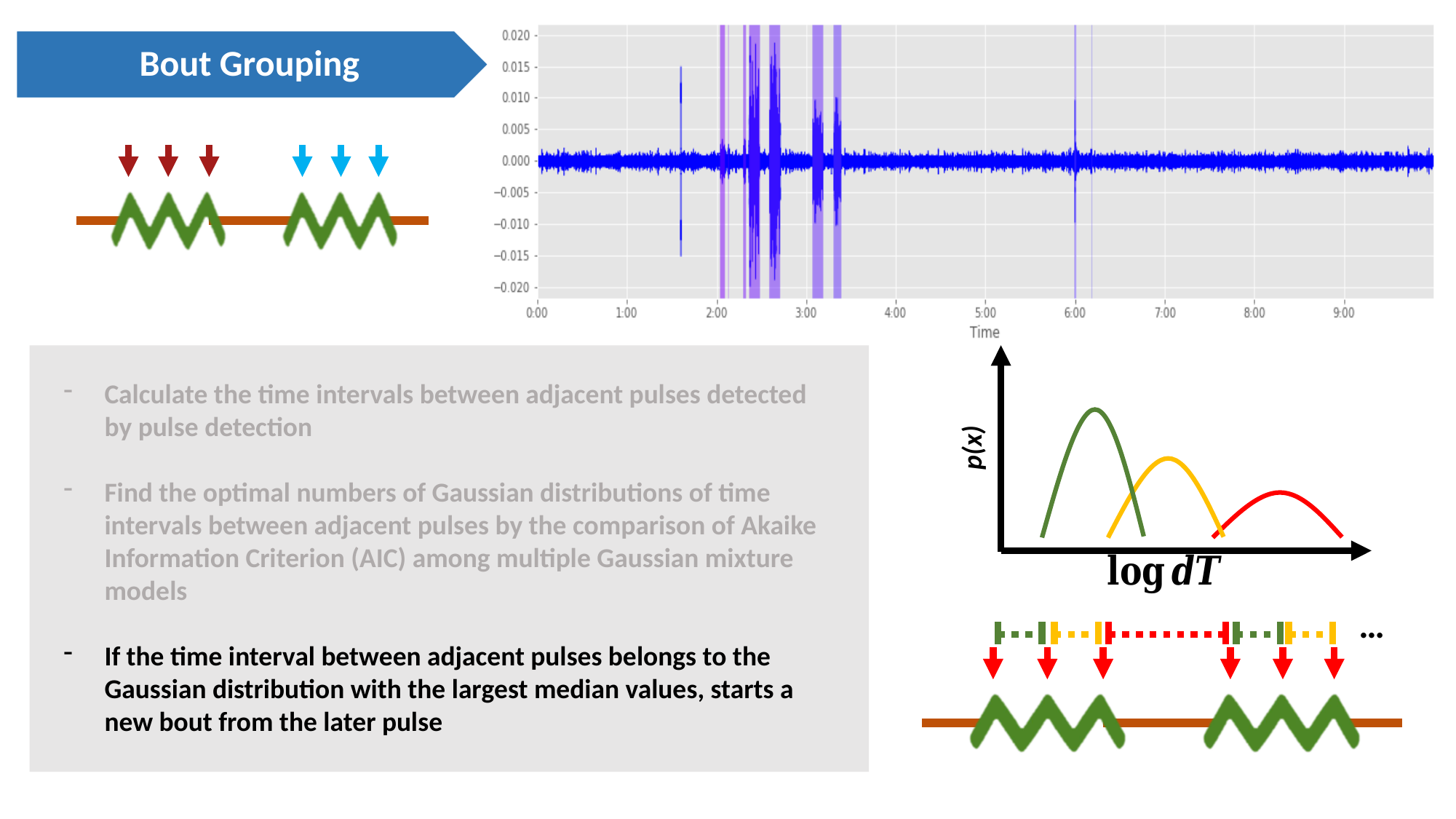

p(x)
Calculate the time intervals between adjacent pulses detected by pulse detection
Find the optimal numbers of Gaussian distributions of time intervals between adjacent pulses by the comparison of Akaike Information Criterion (AIC) among multiple Gaussian mixture models
If the time interval between adjacent pulses belongs to the Gaussian distribution with the largest median values, starts a new bout from the later pulse
…

## Slide 9
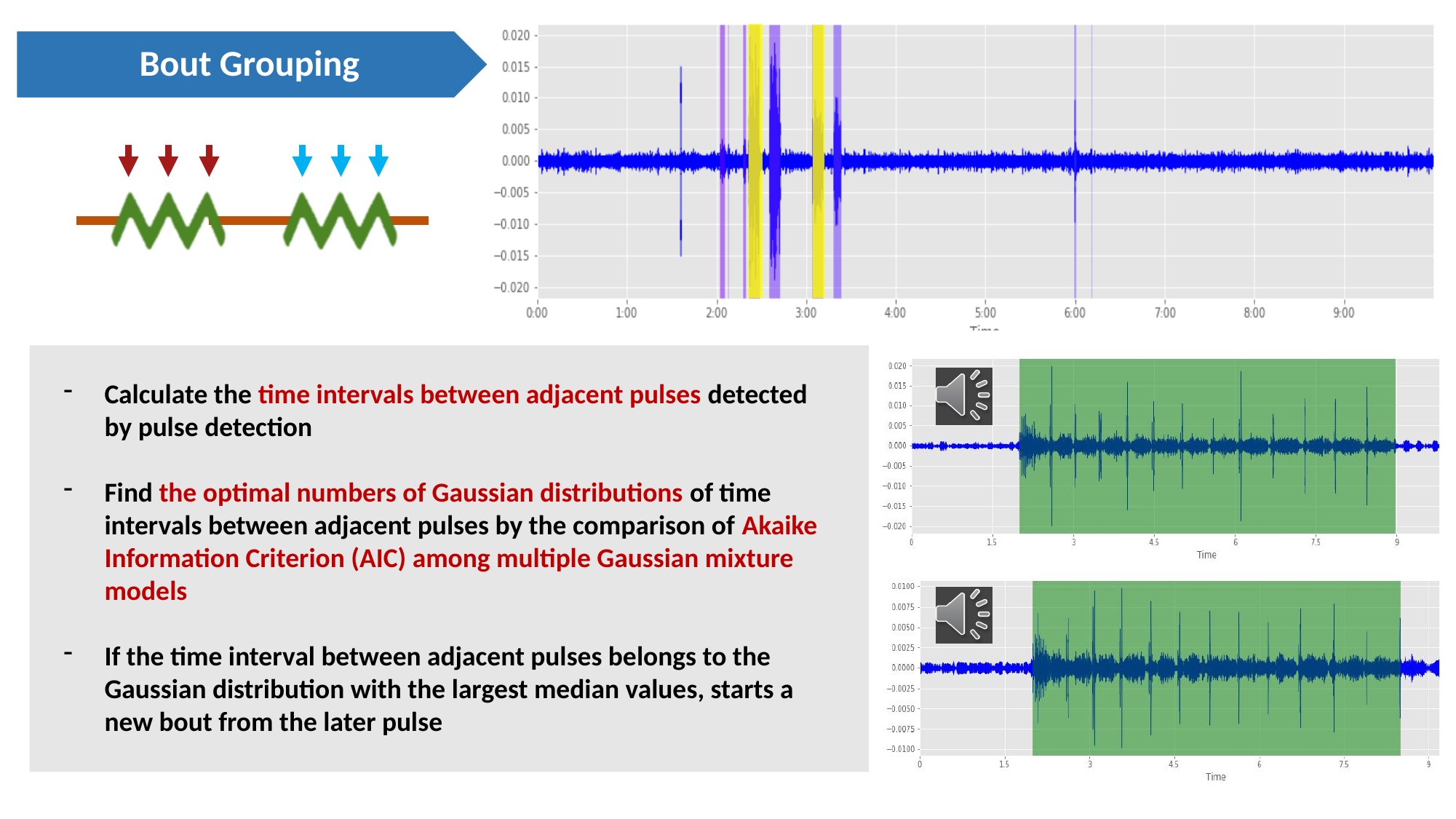

Calculate the time intervals between adjacent pulses detected by pulse detection
Find the optimal numbers of Gaussian distributions of time intervals between adjacent pulses by the comparison of Akaike Information Criterion (AIC) among multiple Gaussian mixture models
If the time interval between adjacent pulses belongs to the Gaussian distribution with the largest median values, starts a new bout from the later pulse
