# Supplementary figures and images for: Vibroscape analysis reveals acoustic niche overlap and plastic alteration of vibratory courtship signals in ground-dwelling wolf spiders (part 2 of 2)
Source: Commun Biol. 2024 Jan 5;7:23. doi: 10.1038/s42003-023-05700-6 (PMC10770364; doi:10.1038/s42003-023-05700-6)

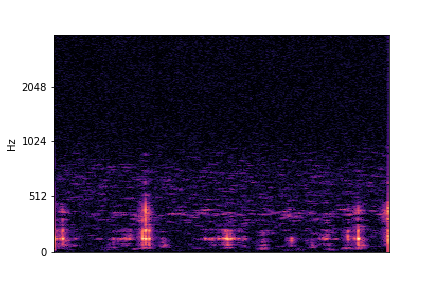

Supplement: Supplementary file 5 — Supplementary Audio [file 42003_2023_5700_MOESM5_ESM.zip › Supplementary_S3/unknown2/spectrogram/180520_D05_08_2_wavchunk31_f_GMM_10.png]

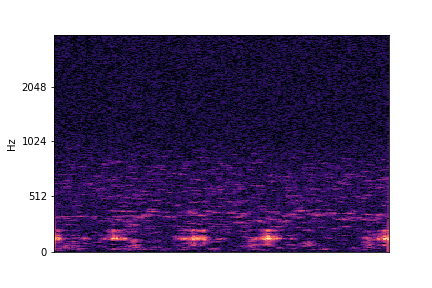

Supplement: Supplementary file 5 — Supplementary Audio [file 42003_2023_5700_MOESM5_ESM.zip › Supplementary_S3/unknown2/spectrogram/180520_D05_08_2_wavchunk31_f_GMM_12.png]

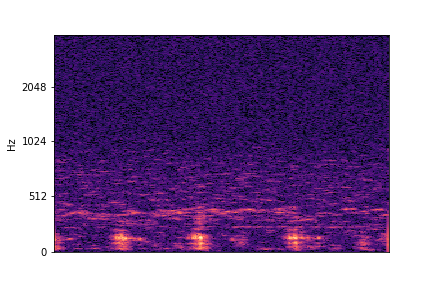

Supplement: Supplementary file 5 — Supplementary Audio [file 42003_2023_5700_MOESM5_ESM.zip › Supplementary_S3/unknown2/spectrogram/180520_D05_08_2_wavchunk31_f_GMM_41.png]

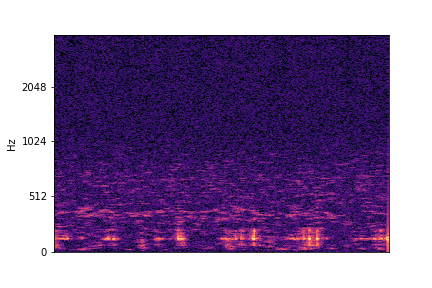

Supplement: Supplementary file 5 — Supplementary Audio [file 42003_2023_5700_MOESM5_ESM.zip › Supplementary_S3/unknown2/spectrogram/180520_D05_08_2_wavchunk31_f_GMM_9.png]

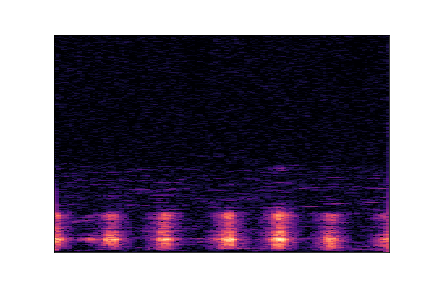

Supplement: Supplementary file 5 — Supplementary Audio [file 42003_2023_5700_MOESM5_ESM.zip › Supplementary_S3/unknown2/spectrogram/180522_B06_160_wavchunk31_f_GMM_14.png]

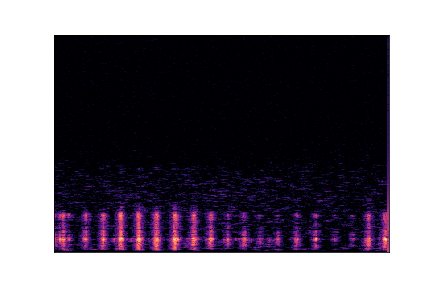

Supplement: Supplementary file 5 — Supplementary Audio [file 42003_2023_5700_MOESM5_ESM.zip › Supplementary_S3/unknown2/spectrogram/180522_B06_160_wavchunk31_f_GMM_15.png]

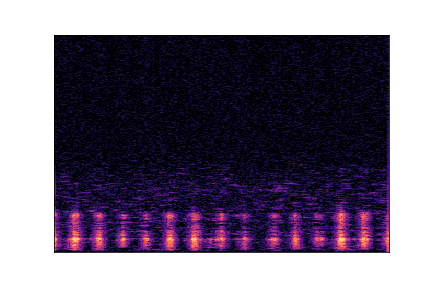

Supplement: Supplementary file 5 — Supplementary Audio [file 42003_2023_5700_MOESM5_ESM.zip › Supplementary_S3/unknown2/spectrogram/180522_B06_160_wavchunk31_f_GMM_16.png]

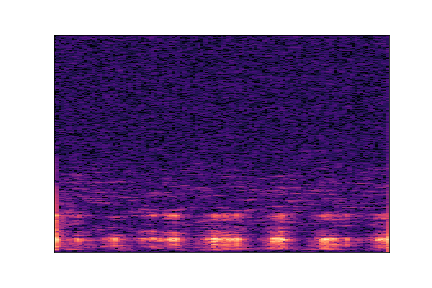

Supplement: Supplementary file 5 — Supplementary Audio [file 42003_2023_5700_MOESM5_ESM.zip › Supplementary_S3/unknown2/spectrogram/180522_B06_160_wavchunk31_f_GMM_17.png]

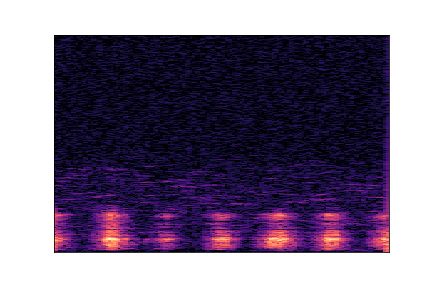

Supplement: Supplementary file 5 — Supplementary Audio [file 42003_2023_5700_MOESM5_ESM.zip › Supplementary_S3/unknown2/spectrogram/180522_B06_160_wavchunk31_f_GMM_18.png]

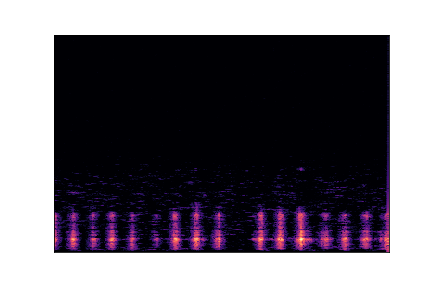

Supplement: Supplementary file 5 — Supplementary Audio [file 42003_2023_5700_MOESM5_ESM.zip › Supplementary_S3/unknown2/spectrogram/180522_B06_160_wavchunk31_f_GMM_19.png]

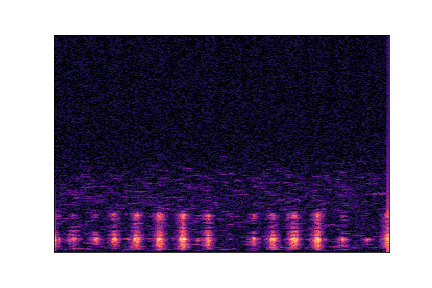

Supplement: Supplementary file 5 — Supplementary Audio [file 42003_2023_5700_MOESM5_ESM.zip › Supplementary_S3/unknown2/spectrogram/180522_B06_160_wavchunk31_f_GMM_20.png]

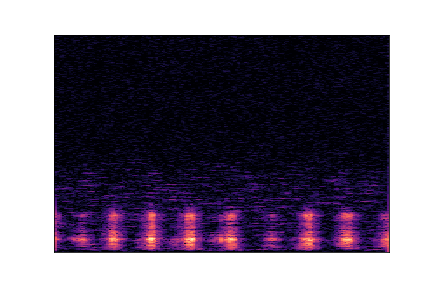

Supplement: Supplementary file 5 — Supplementary Audio [file 42003_2023_5700_MOESM5_ESM.zip › Supplementary_S3/unknown2/spectrogram/180522_B06_160_wavchunk31_f_GMM_22.png]

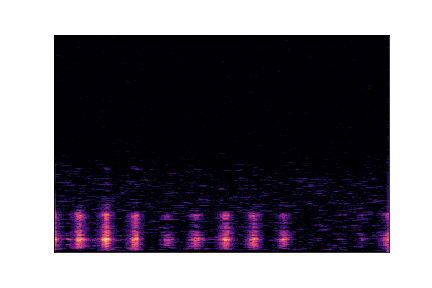

Supplement: Supplementary file 5 — Supplementary Audio [file 42003_2023_5700_MOESM5_ESM.zip › Supplementary_S3/unknown2/spectrogram/180522_B06_160_wavchunk31_f_GMM_23.png]

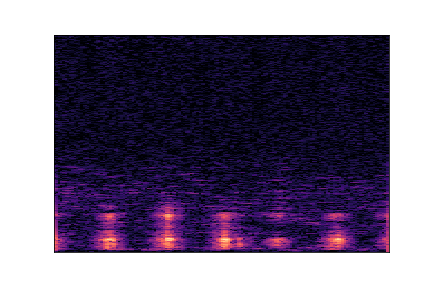

Supplement: Supplementary file 5 — Supplementary Audio [file 42003_2023_5700_MOESM5_ESM.zip › Supplementary_S3/unknown2/spectrogram/180522_B06_160_wavchunk31_f_GMM_31.png]

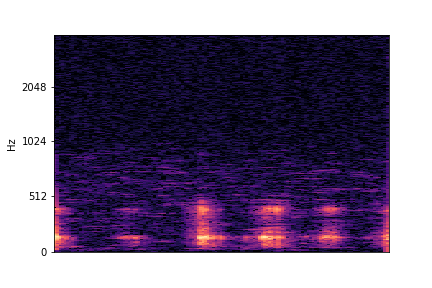

Supplement: Supplementary file 5 — Supplementary Audio [file 42003_2023_5700_MOESM5_ESM.zip › Supplementary_S3/unknown2/spectrogram/180522_B06_160_wavchunk31_f_GMM_34.png]

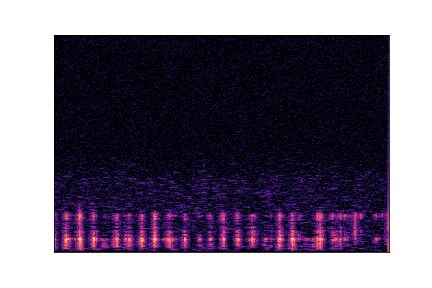

Supplement: Supplementary file 5 — Supplementary Audio [file 42003_2023_5700_MOESM5_ESM.zip › Supplementary_S3/unknown2/spectrogram/180522_B06_160_wavchunk31_f_GMM_7.png]

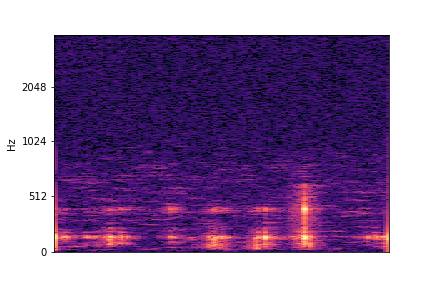

Supplement: Supplementary file 5 — Supplementary Audio [file 42003_2023_5700_MOESM5_ESM.zip › Supplementary_S3/unknown2/spectrogram/180522_B06_160_wavchunk32_f_GMM_5.png]

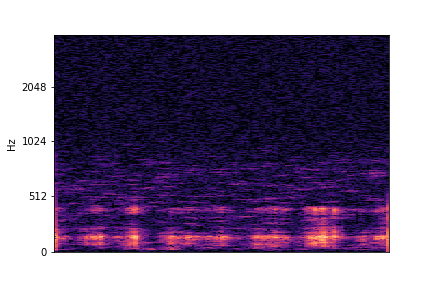

Supplement: Supplementary file 5 — Supplementary Audio [file 42003_2023_5700_MOESM5_ESM.zip › Supplementary_S3/unknown2/spectrogram/180522_B06_160_wavchunk32_f_GMM_6.png]

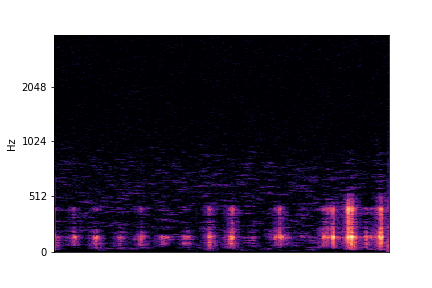

Supplement: Supplementary file 5 — Supplementary Audio [file 42003_2023_5700_MOESM5_ESM.zip › Supplementary_S3/unknown2/spectrogram/180522_B06_160_wavchunk32_f_GMM_8.png]

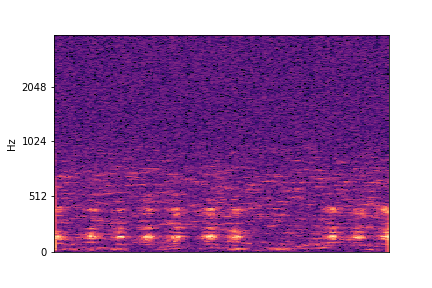

Supplement: Supplementary file 5 — Supplementary Audio [file 42003_2023_5700_MOESM5_ESM.zip › Supplementary_S3/unknown2/spectrogram/180522_B06_161_wavchunk8_f_GMM_16.png]

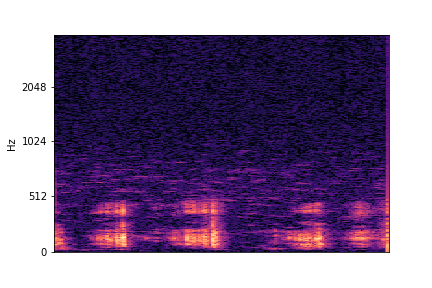

Supplement: Supplementary file 5 — Supplementary Audio [file 42003_2023_5700_MOESM5_ESM.zip › Supplementary_S3/unknown2/spectrogram/180522_B07_08_wavchunk29_f_GMM_8.png]

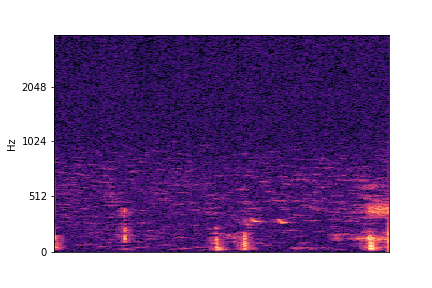

Supplement: Supplementary file 5 — Supplementary Audio [file 42003_2023_5700_MOESM5_ESM.zip › Supplementary_S3/unknown2/spectrogram/180522_B07_08_wavchunk30_f_GMM_5.png]

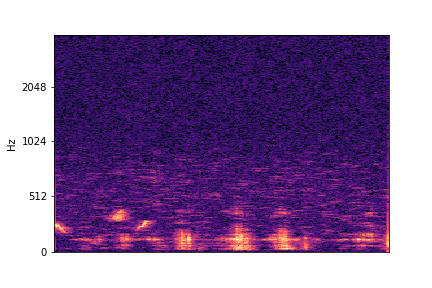

Supplement: Supplementary file 5 — Supplementary Audio [file 42003_2023_5700_MOESM5_ESM.zip › Supplementary_S3/unknown2/spectrogram/180522_B07_08_wavchunk30_f_GMM_7.png]

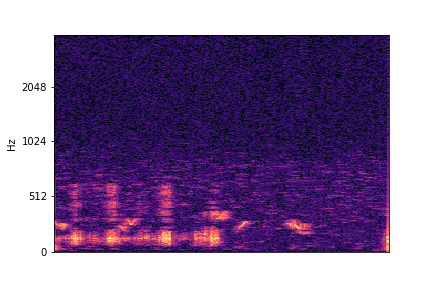

Supplement: Supplementary file 5 — Supplementary Audio [file 42003_2023_5700_MOESM5_ESM.zip › Supplementary_S3/unknown2/spectrogram/180522_B07_08_wavchunk30_f_GMM_8.png]

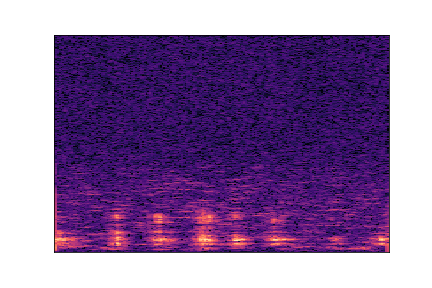

Supplement: Supplementary file 5 — Supplementary Audio [file 42003_2023_5700_MOESM5_ESM.zip › Supplementary_S3/unknown2/spectrogram/180522_B11_160_wavchunk3_f_GMM_35.png]

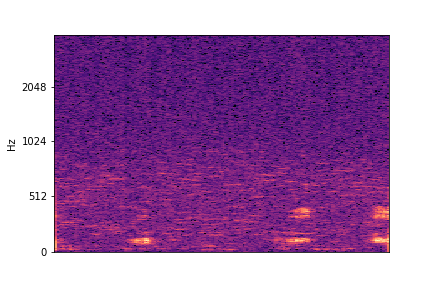

Supplement: Supplementary file 5 — Supplementary Audio [file 42003_2023_5700_MOESM5_ESM.zip › Supplementary_S3/unknown2/spectrogram/180522_B12_08_wavchunk0_f_GMM_18.png]

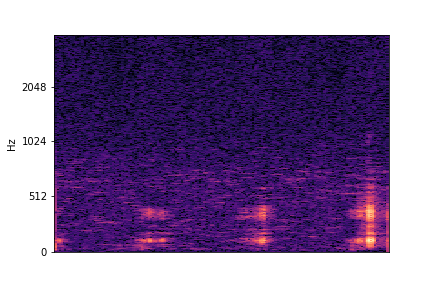

Supplement: Supplementary file 5 — Supplementary Audio [file 42003_2023_5700_MOESM5_ESM.zip › Supplementary_S3/unknown2/spectrogram/180522_B12_08_wavchunk0_f_GMM_19.png]

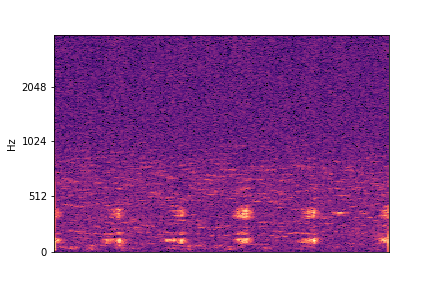

Supplement: Supplementary file 5 — Supplementary Audio [file 42003_2023_5700_MOESM5_ESM.zip › Supplementary_S3/unknown2/spectrogram/180522_B12_08_wavchunk0_f_GMM_20.png]

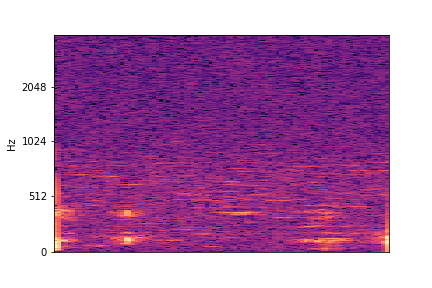

Supplement: Supplementary file 5 — Supplementary Audio [file 42003_2023_5700_MOESM5_ESM.zip › Supplementary_S3/unknown2/spectrogram/180522_B12_08_wavchunk0_f_GMM_24.png]

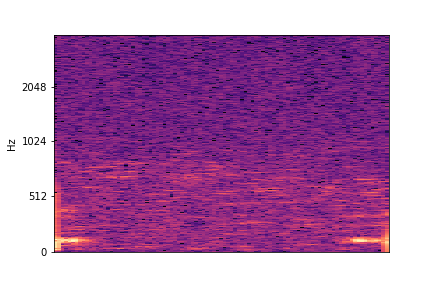

Supplement: Supplementary file 5 — Supplementary Audio [file 42003_2023_5700_MOESM5_ESM.zip › Supplementary_S3/unknown2/spectrogram/180522_B12_08_wavchunk0_f_GMM_25.png]

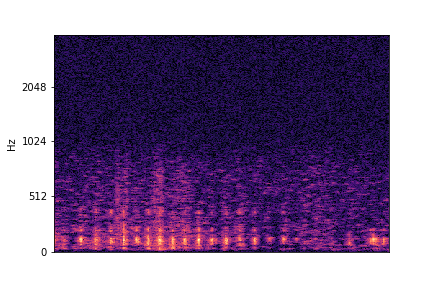

Supplement: Supplementary file 5 — Supplementary Audio [file 42003_2023_5700_MOESM5_ESM.zip › Supplementary_S3/unknown2/spectrogram/180525_D01_08_wavchunk18_f_GMM_23.png]

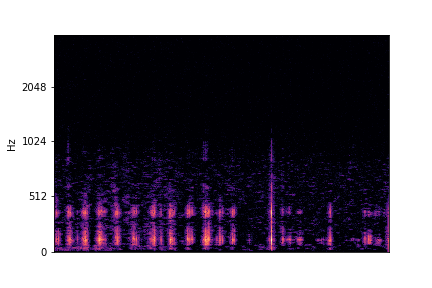

Supplement: Supplementary file 5 — Supplementary Audio [file 42003_2023_5700_MOESM5_ESM.zip › Supplementary_S3/unknown2/spectrogram/180525_D01_08_wavchunk31_f_GMM_10.png]

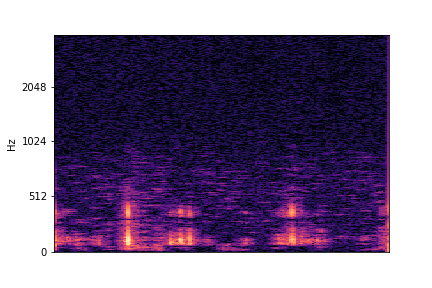

Supplement: Supplementary file 5 — Supplementary Audio [file 42003_2023_5700_MOESM5_ESM.zip › Supplementary_S3/unknown2/spectrogram/180525_D01_08_wavchunk31_f_GMM_19.png]

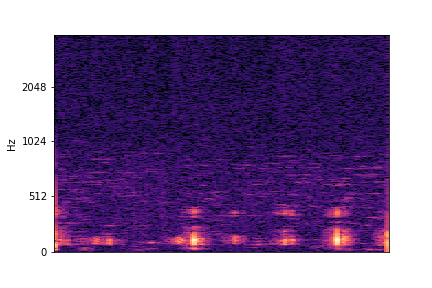

Supplement: Supplementary file 5 — Supplementary Audio [file 42003_2023_5700_MOESM5_ESM.zip › Supplementary_S3/unknown2/spectrogram/180525_D01_08_wavchunk31_f_GMM_20.png]

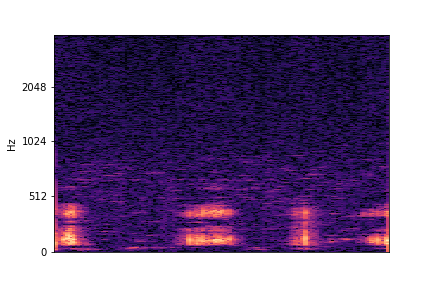

Supplement: Supplementary file 5 — Supplementary Audio [file 42003_2023_5700_MOESM5_ESM.zip › Supplementary_S3/unknown2/spectrogram/180525_D01_08_wavchunk31_f_GMM_31.png]

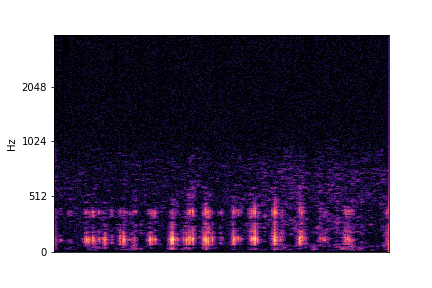

Supplement: Supplementary file 5 — Supplementary Audio [file 42003_2023_5700_MOESM5_ESM.zip › Supplementary_S3/unknown2/spectrogram/180525_D01_08_wavchunk31_f_GMM_35.png]

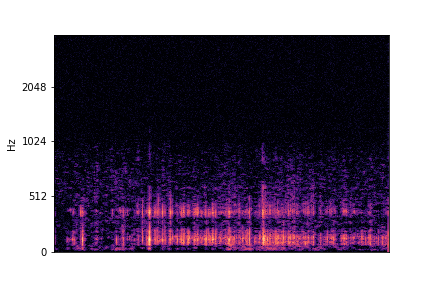

Supplement: Supplementary file 5 — Supplementary Audio [file 42003_2023_5700_MOESM5_ESM.zip › Supplementary_S3/unknown2/spectrogram/180525_D01_08_wavchunk31_f_GMM_47.png]

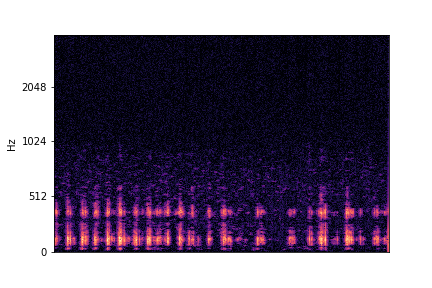

Supplement: Supplementary file 5 — Supplementary Audio [file 42003_2023_5700_MOESM5_ESM.zip › Supplementary_S3/unknown2/spectrogram/180525_D01_08_wavchunk31_f_GMM_6.png]

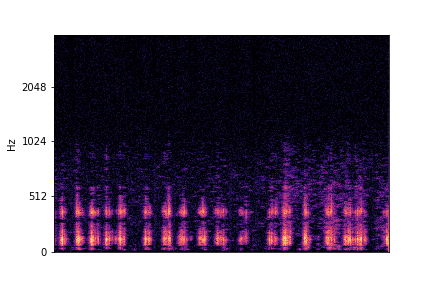

Supplement: Supplementary file 5 — Supplementary Audio [file 42003_2023_5700_MOESM5_ESM.zip › Supplementary_S3/unknown2/spectrogram/180525_D01_08_wavchunk31_f_GMM_7.png]

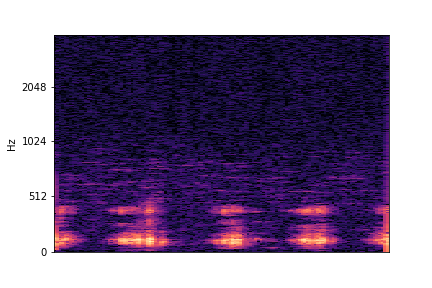

Supplement: Supplementary file 5 — Supplementary Audio [file 42003_2023_5700_MOESM5_ESM.zip › Supplementary_S3/unknown2/spectrogram/180525_D21_08_wavchunk18_f_GMM_67.png]

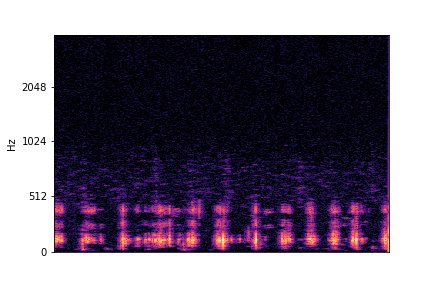

Supplement: Supplementary file 5 — Supplementary Audio [file 42003_2023_5700_MOESM5_ESM.zip › Supplementary_S3/unknown2/spectrogram/180525_D21_08_wavchunk18_f_GMM_69.png]

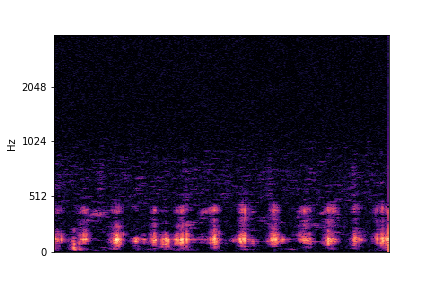

Supplement: Supplementary file 5 — Supplementary Audio [file 42003_2023_5700_MOESM5_ESM.zip › Supplementary_S3/unknown2/spectrogram/180525_D21_08_wavchunk18_f_GMM_70.png]

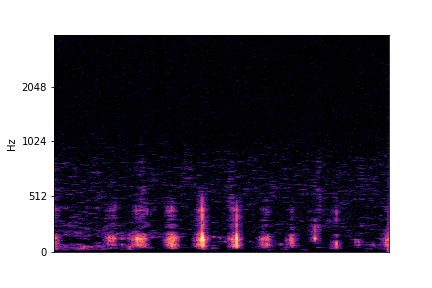

Supplement: Supplementary file 5 — Supplementary Audio [file 42003_2023_5700_MOESM5_ESM.zip › Supplementary_S3/unknown2/spectrogram/180525_D21_08_wavchunk19_f_GMM_59.png]

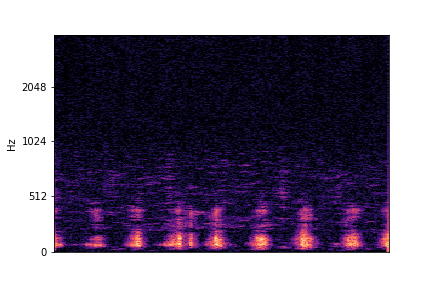

Supplement: Supplementary file 5 — Supplementary Audio [file 42003_2023_5700_MOESM5_ESM.zip › Supplementary_S3/unknown2/spectrogram/180525_D21_08_wavchunk19_f_GMM_63.png]

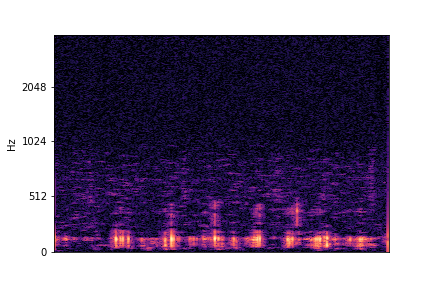

Supplement: Supplementary file 5 — Supplementary Audio [file 42003_2023_5700_MOESM5_ESM.zip › Supplementary_S3/unknown2/spectrogram/180525_D21_08_wavchunk19_f_GMM_65.png]

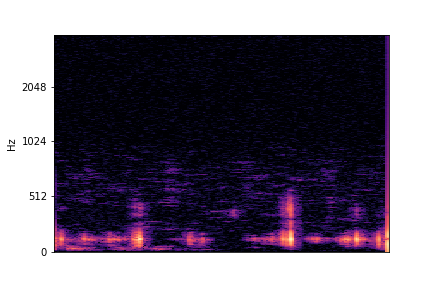

Supplement: Supplementary file 5 — Supplementary Audio [file 42003_2023_5700_MOESM5_ESM.zip › Supplementary_S3/unknown2/spectrogram/180525_D21_08_wavchunk19_f_GMM_69.png]

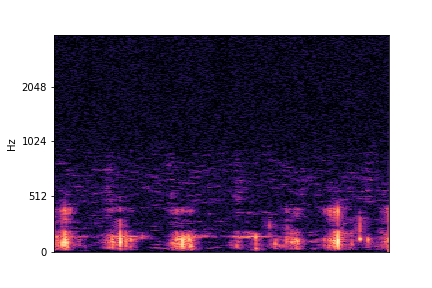

Supplement: Supplementary file 5 — Supplementary Audio [file 42003_2023_5700_MOESM5_ESM.zip › Supplementary_S3/unknown2/spectrogram/180525_D21_08_wavchunk19_f_GMM_75.png]

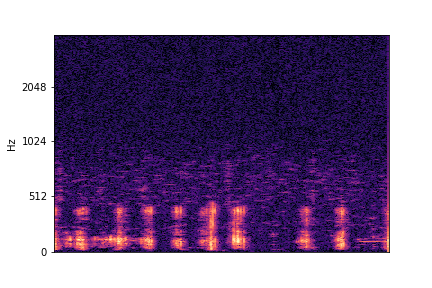

Supplement: Supplementary file 5 — Supplementary Audio [file 42003_2023_5700_MOESM5_ESM.zip › Supplementary_S3/unknown2/spectrogram/180525_D21_08_wavchunk19_f_GMM_76.png]

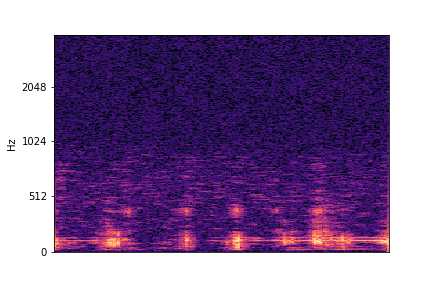

Supplement: Supplementary file 5 — Supplementary Audio [file 42003_2023_5700_MOESM5_ESM.zip › Supplementary_S3/unknown2/spectrogram/180525_D21_08_wavchunk19_f_GMM_77.png]

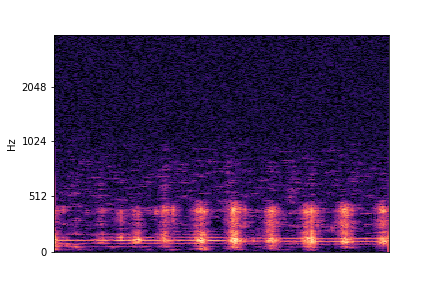

Supplement: Supplementary file 5 — Supplementary Audio [file 42003_2023_5700_MOESM5_ESM.zip › Supplementary_S3/unknown2/spectrogram/180525_D21_08_wavchunk19_f_GMM_79.png]

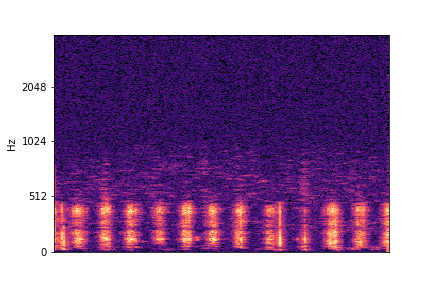

Supplement: Supplementary file 5 — Supplementary Audio [file 42003_2023_5700_MOESM5_ESM.zip › Supplementary_S3/unknown2/spectrogram/180525_D21_08_wavchunk19_f_GMM_82.png]

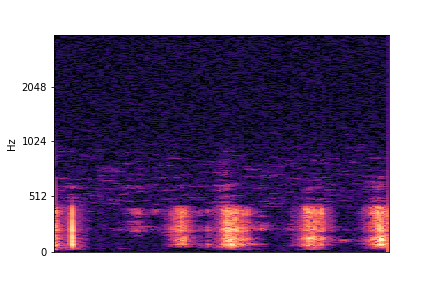

Supplement: Supplementary file 5 — Supplementary Audio [file 42003_2023_5700_MOESM5_ESM.zip › Supplementary_S3/unknown2/spectrogram/180525_D21_08_wavchunk19_f_GMM_92.png]

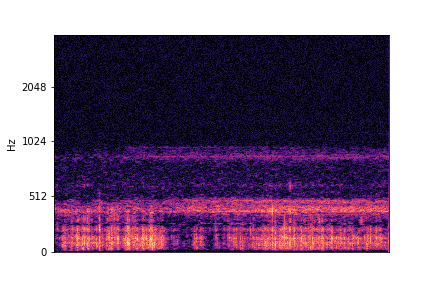

Supplement: Supplementary file 5 — Supplementary Audio [file 42003_2023_5700_MOESM5_ESM.zip › Supplementary_S3/unknown2/spectrogram/180618_C11_16_1_wavchunk32_f_GMM_10.png]

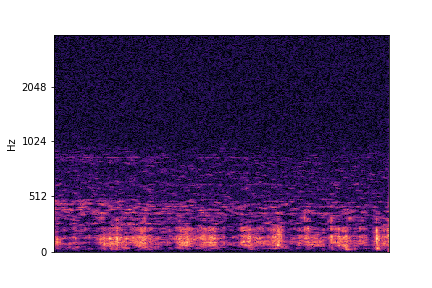

Supplement: Supplementary file 5 — Supplementary Audio [file 42003_2023_5700_MOESM5_ESM.zip › Supplementary_S3/unknown2/spectrogram/180618_C11_16_1_wavchunk32_f_GMM_12.png]

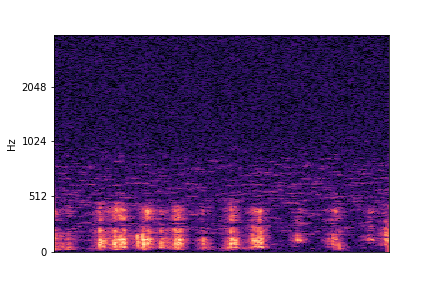

Supplement: Supplementary file 5 — Supplementary Audio [file 42003_2023_5700_MOESM5_ESM.zip › Supplementary_S3/unknown2/spectrogram/180618_C12_08_wavchunk36_f_GMM_10.png]

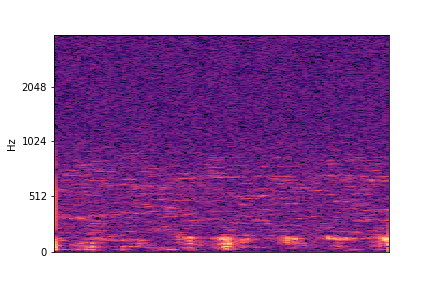

Supplement: Supplementary file 5 — Supplementary Audio [file 42003_2023_5700_MOESM5_ESM.zip › Supplementary_S3/unknown2/spectrogram/180618_C12_16_1_wavchunk13_f_GMM_40.png]

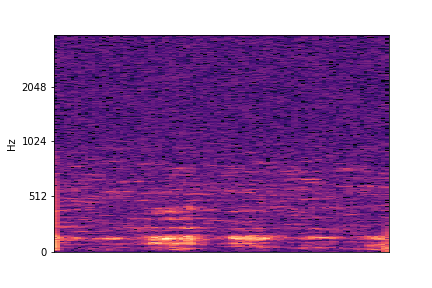

Supplement: Supplementary file 5 — Supplementary Audio [file 42003_2023_5700_MOESM5_ESM.zip › Supplementary_S3/unknown2/spectrogram/180618_C12_16_1_wavchunk13_f_GMM_60.png]

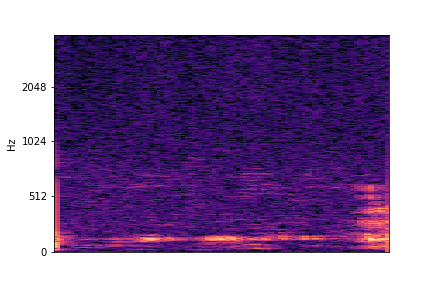

Supplement: Supplementary file 5 — Supplementary Audio [file 42003_2023_5700_MOESM5_ESM.zip › Supplementary_S3/unknown2/spectrogram/180618_C13_08_wavchunk19_f_GMM_54.png]

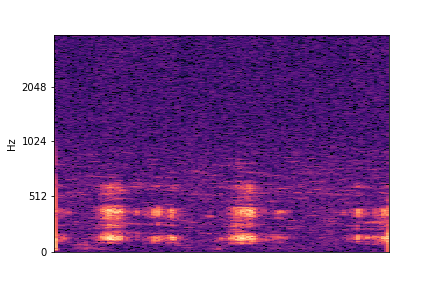

Supplement: Supplementary file 5 — Supplementary Audio [file 42003_2023_5700_MOESM5_ESM.zip › Supplementary_S3/unknown2/spectrogram/180618_C13_08_wavchunk24_f_GMM_11.png]

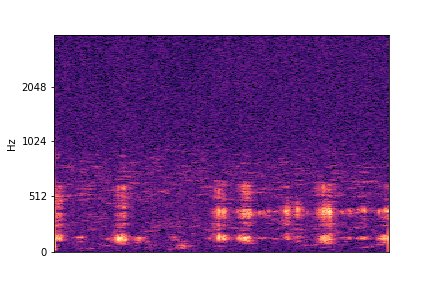

Supplement: Supplementary file 5 — Supplementary Audio [file 42003_2023_5700_MOESM5_ESM.zip › Supplementary_S3/unknown2/spectrogram/180618_C13_08_wavchunk24_f_GMM_12.png]

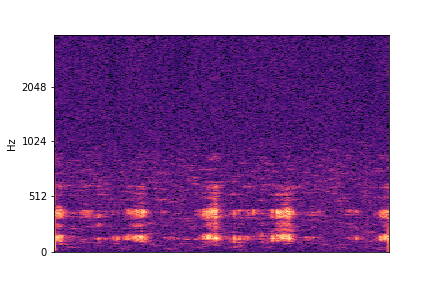

Supplement: Supplementary file 5 — Supplementary Audio [file 42003_2023_5700_MOESM5_ESM.zip › Supplementary_S3/unknown2/spectrogram/180618_C13_08_wavchunk24_f_GMM_13.png]

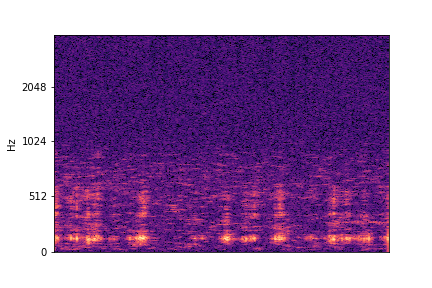

Supplement: Supplementary file 5 — Supplementary Audio [file 42003_2023_5700_MOESM5_ESM.zip › Supplementary_S3/unknown2/spectrogram/180618_C13_08_wavchunk24_f_GMM_14.png]

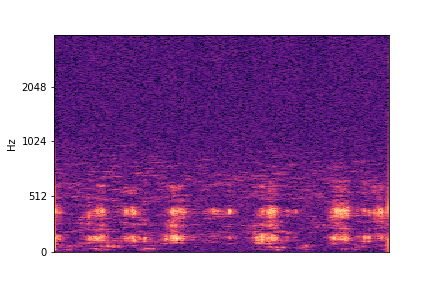

Supplement: Supplementary file 5 — Supplementary Audio [file 42003_2023_5700_MOESM5_ESM.zip › Supplementary_S3/unknown2/spectrogram/180618_C13_08_wavchunk24_f_GMM_15.png]

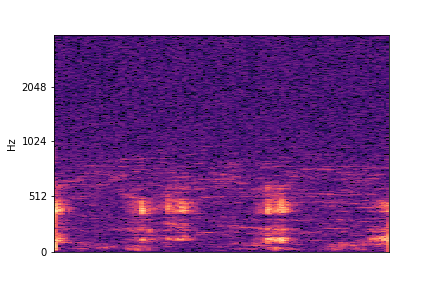

Supplement: Supplementary file 5 — Supplementary Audio [file 42003_2023_5700_MOESM5_ESM.zip › Supplementary_S3/unknown2/spectrogram/180618_C13_08_wavchunk24_f_GMM_17.png]

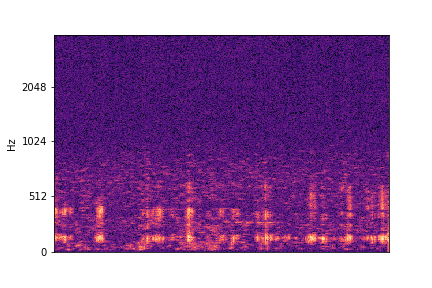

Supplement: Supplementary file 5 — Supplementary Audio [file 42003_2023_5700_MOESM5_ESM.zip › Supplementary_S3/unknown2/spectrogram/180618_C13_08_wavchunk24_f_GMM_18.png]

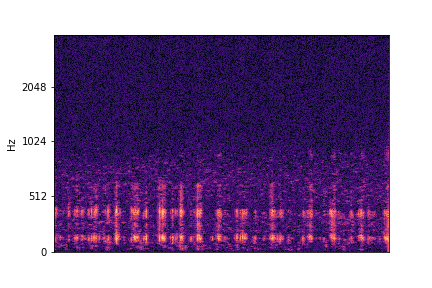

Supplement: Supplementary file 5 — Supplementary Audio [file 42003_2023_5700_MOESM5_ESM.zip › Supplementary_S3/unknown2/spectrogram/180618_C13_08_wavchunk24_f_GMM_19.png]

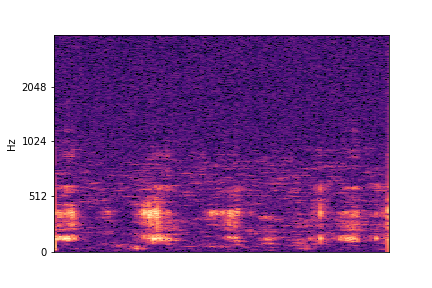

Supplement: Supplementary file 5 — Supplementary Audio [file 42003_2023_5700_MOESM5_ESM.zip › Supplementary_S3/unknown2/spectrogram/180618_C13_08_wavchunk24_f_GMM_20.png]

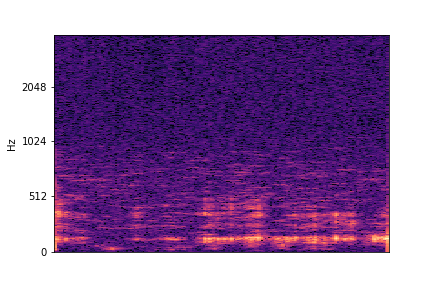

Supplement: Supplementary file 5 — Supplementary Audio [file 42003_2023_5700_MOESM5_ESM.zip › Supplementary_S3/unknown2/spectrogram/180618_C13_08_wavchunk24_f_GMM_21.png]

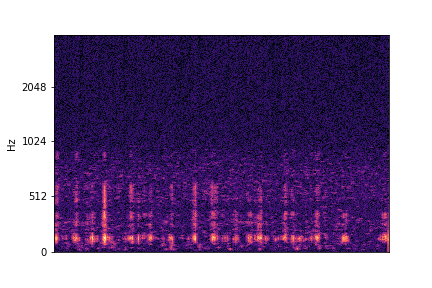

Supplement: Supplementary file 5 — Supplementary Audio [file 42003_2023_5700_MOESM5_ESM.zip › Supplementary_S3/unknown2/spectrogram/180618_C13_08_wavchunk24_f_GMM_22.png]

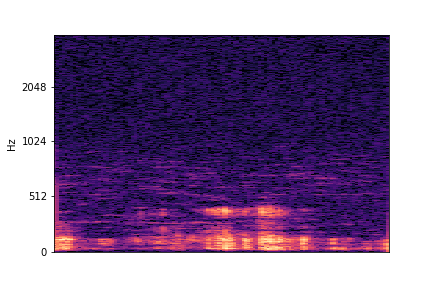

Supplement: Supplementary file 5 — Supplementary Audio [file 42003_2023_5700_MOESM5_ESM.zip › Supplementary_S3/unknown2/spectrogram/180618_C13_08_wavchunk24_f_GMM_23.png]

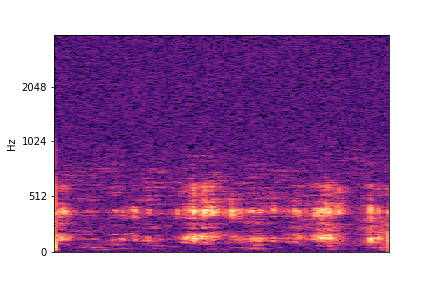

Supplement: Supplementary file 5 — Supplementary Audio [file 42003_2023_5700_MOESM5_ESM.zip › Supplementary_S3/unknown2/spectrogram/180618_C13_08_wavchunk24_f_GMM_31.png]

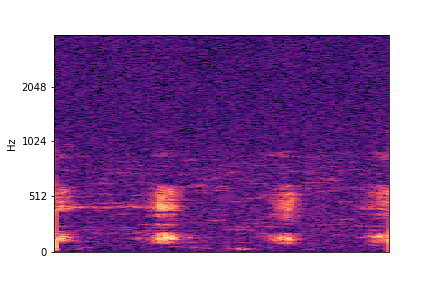

Supplement: Supplementary file 5 — Supplementary Audio [file 42003_2023_5700_MOESM5_ESM.zip › Supplementary_S3/unknown2/spectrogram/180618_C13_08_wavchunk24_f_GMM_34.png]

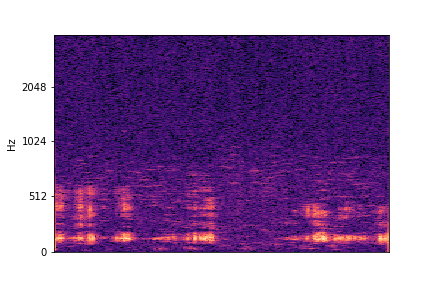

Supplement: Supplementary file 5 — Supplementary Audio [file 42003_2023_5700_MOESM5_ESM.zip › Supplementary_S3/unknown2/spectrogram/180618_C13_08_wavchunk24_f_GMM_35.png]

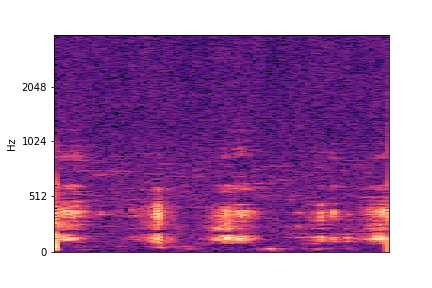

Supplement: Supplementary file 5 — Supplementary Audio [file 42003_2023_5700_MOESM5_ESM.zip › Supplementary_S3/unknown2/spectrogram/180618_C13_08_wavchunk24_f_GMM_37.png]

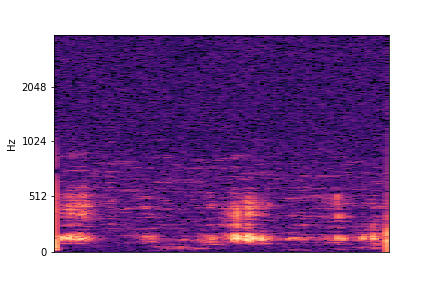

Supplement: Supplementary file 5 — Supplementary Audio [file 42003_2023_5700_MOESM5_ESM.zip › Supplementary_S3/unknown2/spectrogram/180618_C13_08_wavchunk24_f_GMM_38.png]

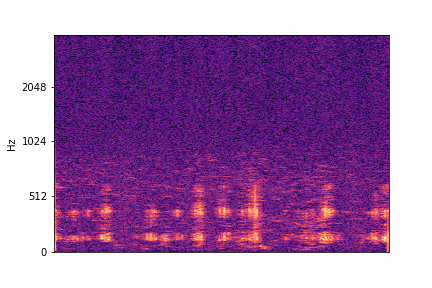

Supplement: Supplementary file 5 — Supplementary Audio [file 42003_2023_5700_MOESM5_ESM.zip › Supplementary_S3/unknown2/spectrogram/180618_C13_08_wavchunk24_f_GMM_44.png]

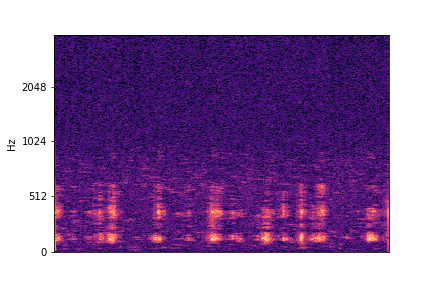

Supplement: Supplementary file 5 — Supplementary Audio [file 42003_2023_5700_MOESM5_ESM.zip › Supplementary_S3/unknown2/spectrogram/180618_C13_08_wavchunk24_f_GMM_48.png]

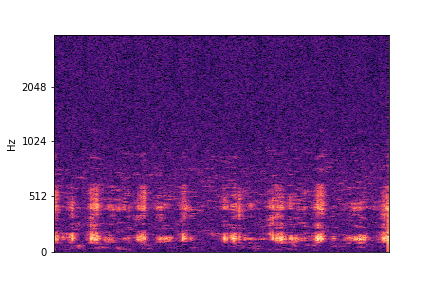

Supplement: Supplementary file 5 — Supplementary Audio [file 42003_2023_5700_MOESM5_ESM.zip › Supplementary_S3/unknown2/spectrogram/180618_C13_08_wavchunk24_f_GMM_52.png]

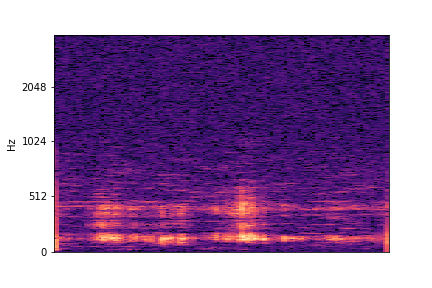

Supplement: Supplementary file 5 — Supplementary Audio [file 42003_2023_5700_MOESM5_ESM.zip › Supplementary_S3/unknown2/spectrogram/180618_C13_08_wavchunk24_f_GMM_54.png]

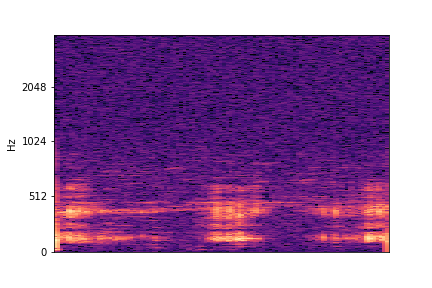

Supplement: Supplementary file 5 — Supplementary Audio [file 42003_2023_5700_MOESM5_ESM.zip › Supplementary_S3/unknown2/spectrogram/180618_C13_08_wavchunk24_f_GMM_58.png]

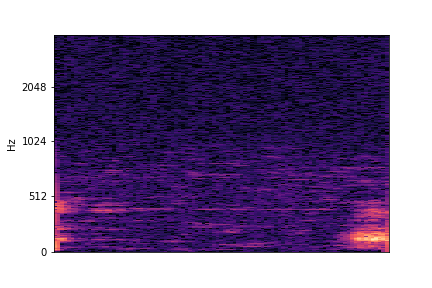

Supplement: Supplementary file 5 — Supplementary Audio [file 42003_2023_5700_MOESM5_ESM.zip › Supplementary_S3/unknown2/spectrogram/180618_C13_08_wavchunk24_f_GMM_60.png]

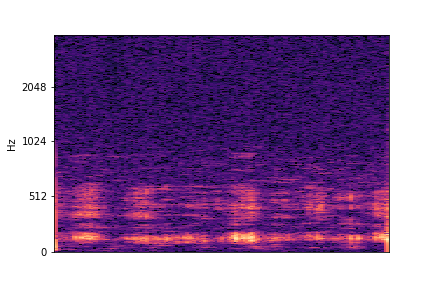

Supplement: Supplementary file 5 — Supplementary Audio [file 42003_2023_5700_MOESM5_ESM.zip › Supplementary_S3/unknown2/spectrogram/180618_C13_08_wavchunk24_f_GMM_64.png]

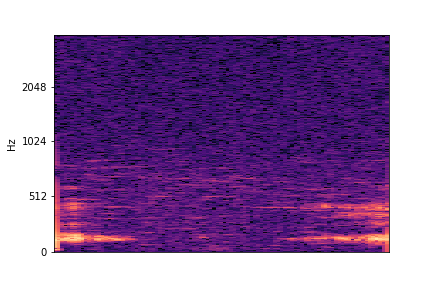

Supplement: Supplementary file 5 — Supplementary Audio [file 42003_2023_5700_MOESM5_ESM.zip › Supplementary_S3/unknown2/spectrogram/180618_C13_08_wavchunk24_f_GMM_65.png]

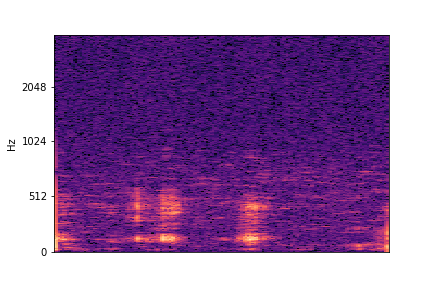

Supplement: Supplementary file 5 — Supplementary Audio [file 42003_2023_5700_MOESM5_ESM.zip › Supplementary_S3/unknown2/spectrogram/180618_C13_08_wavchunk24_f_GMM_67.png]

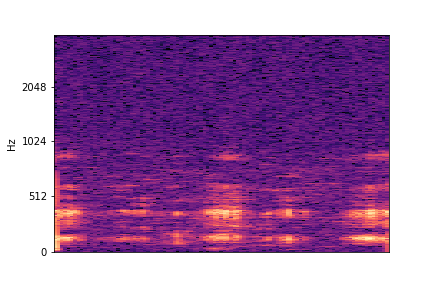

Supplement: Supplementary file 5 — Supplementary Audio [file 42003_2023_5700_MOESM5_ESM.zip › Supplementary_S3/unknown2/spectrogram/180618_C13_08_wavchunk24_f_GMM_9.png]

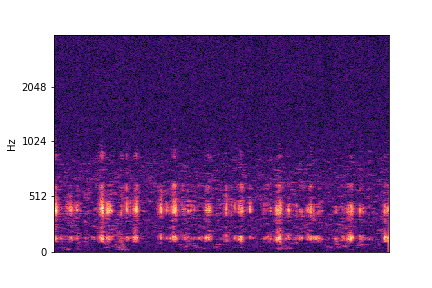

Supplement: Supplementary file 5 — Supplementary Audio [file 42003_2023_5700_MOESM5_ESM.zip › Supplementary_S3/unknown2/spectrogram/180618_C13_16_1_wavchunk13_f_GMM_14.png]

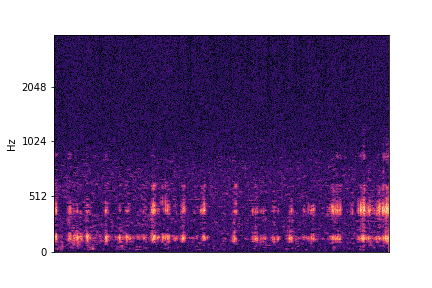

Supplement: Supplementary file 5 — Supplementary Audio [file 42003_2023_5700_MOESM5_ESM.zip › Supplementary_S3/unknown2/spectrogram/180618_C13_16_1_wavchunk13_f_GMM_16.png]

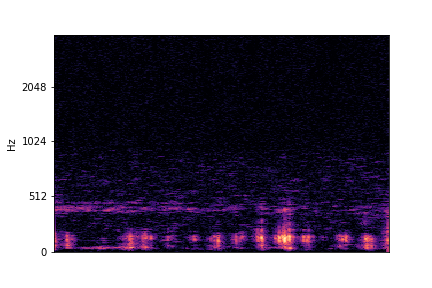

Supplement: Supplementary file 5 — Supplementary Audio [file 42003_2023_5700_MOESM5_ESM.zip › Supplementary_S3/unknown2/spectrogram/180618_C13_16_1_wavchunk13_f_GMM_20.png]

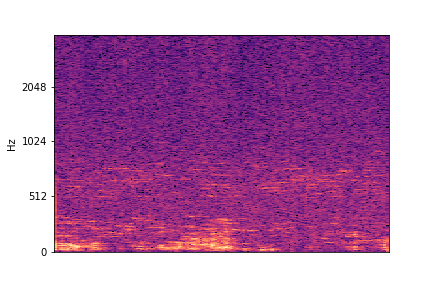

Supplement: Supplementary file 5 — Supplementary Audio [file 42003_2023_5700_MOESM5_ESM.zip › Supplementary_S3/unknown2/spectrogram/180618_C13_16_1_wavchunk14_f_GMM_1.png]

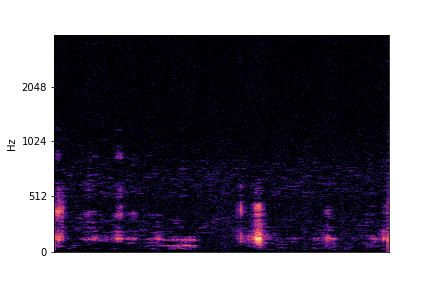

Supplement: Supplementary file 5 — Supplementary Audio [file 42003_2023_5700_MOESM5_ESM.zip › Supplementary_S3/unknown2/spectrogram/180618_C13_16_1_wavchunk20_f_GMM_36.png]

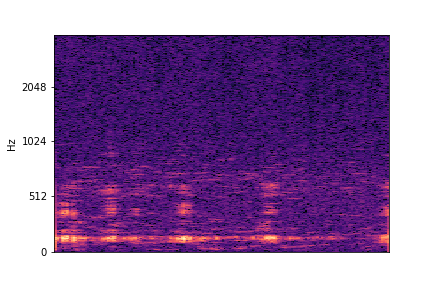

Supplement: Supplementary file 5 — Supplementary Audio [file 42003_2023_5700_MOESM5_ESM.zip › Supplementary_S3/unknown2/spectrogram/180618_C13_16_1_wavchunk20_f_GMM_39.png]

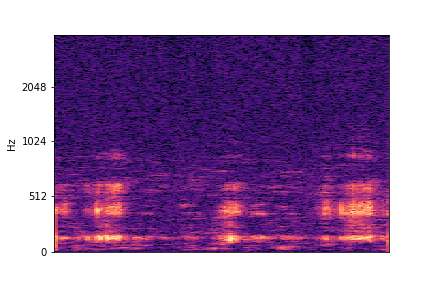

Supplement: Supplementary file 5 — Supplementary Audio [file 42003_2023_5700_MOESM5_ESM.zip › Supplementary_S3/unknown2/spectrogram/180618_C13_16_1_wavchunk20_f_GMM_40.png]

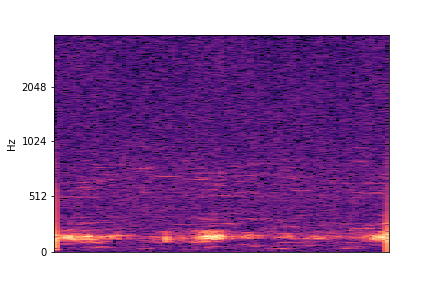

Supplement: Supplementary file 5 — Supplementary Audio [file 42003_2023_5700_MOESM5_ESM.zip › Supplementary_S3/unknown2/spectrogram/180618_C13_16_1_wavchunk20_f_GMM_47.png]

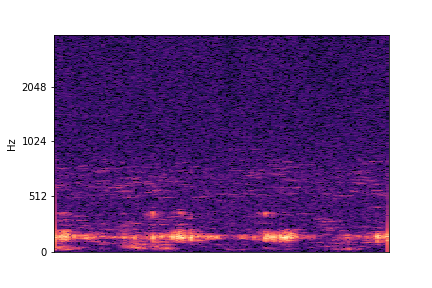

Supplement: Supplementary file 5 — Supplementary Audio [file 42003_2023_5700_MOESM5_ESM.zip › Supplementary_S3/unknown2/spectrogram/180618_C13_16_1_wavchunk20_f_GMM_48.png]

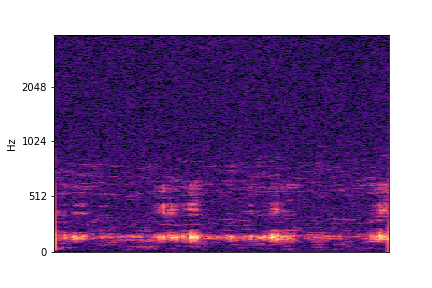

Supplement: Supplementary file 5 — Supplementary Audio [file 42003_2023_5700_MOESM5_ESM.zip › Supplementary_S3/unknown2/spectrogram/180618_C13_16_1_wavchunk20_f_GMM_49.png]

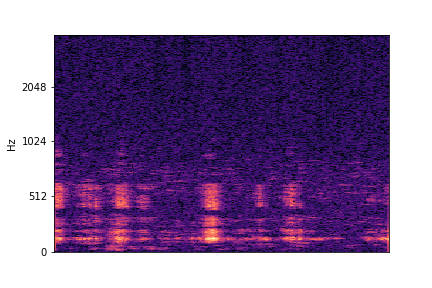

Supplement: Supplementary file 5 — Supplementary Audio [file 42003_2023_5700_MOESM5_ESM.zip › Supplementary_S3/unknown2/spectrogram/180618_C13_16_1_wavchunk20_f_GMM_51.png]

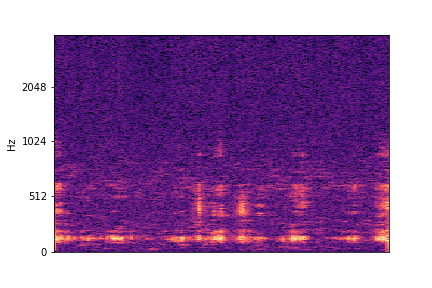

Supplement: Supplementary file 5 — Supplementary Audio [file 42003_2023_5700_MOESM5_ESM.zip › Supplementary_S3/unknown2/spectrogram/180618_C13_16_1_wavchunk20_f_GMM_53.png]

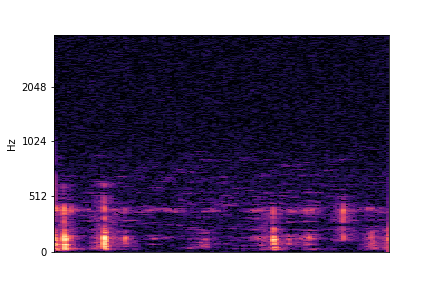

Supplement: Supplementary file 5 — Supplementary Audio [file 42003_2023_5700_MOESM5_ESM.zip › Supplementary_S3/unknown2/spectrogram/180618_C13_16_1_wavchunk45_f_GMM_3.png]

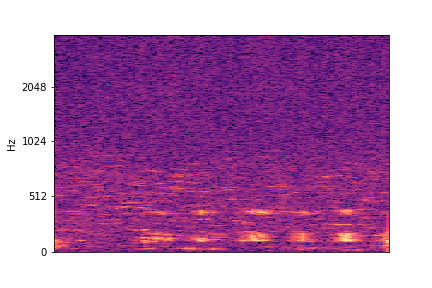

Supplement: Supplementary file 5 — Supplementary Audio [file 42003_2023_5700_MOESM5_ESM.zip › Supplementary_S3/unknown2/spectrogram/180618_E14_161_wavchunk11_f_GMM_3.png]

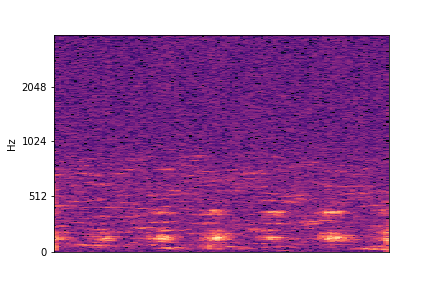

Supplement: Supplementary file 5 — Supplementary Audio [file 42003_2023_5700_MOESM5_ESM.zip › Supplementary_S3/unknown2/spectrogram/180618_E14_161_wavchunk12_f_GMM_105.png]
