# Supplementary figures and images for: Vibroscape analysis reveals acoustic niche overlap and plastic alteration of vibratory courtship signals in ground-dwelling wolf spiders (part 1 of 2)
Source: Commun Biol. 2024 Jan 5;7:23. doi: 10.1038/s42003-023-05700-6 (PMC10770364; doi:10.1038/s42003-023-05700-6)

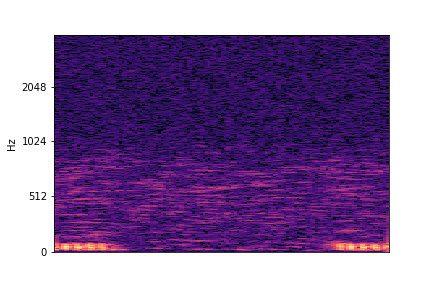

Supplement: Supplementary file 5 — Supplementary Audio [file 42003_2023_5700_MOESM5_ESM.zip › Supplementary_S3/unknown1/spectrogram/180522_A11_08_wavchunk27_f_GMM_22.png]

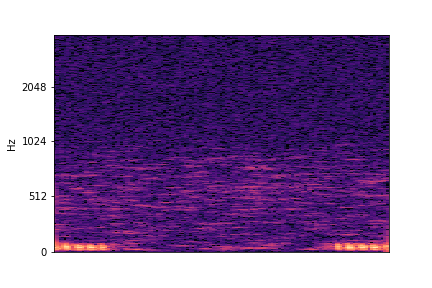

Supplement: Supplementary file 5 — Supplementary Audio [file 42003_2023_5700_MOESM5_ESM.zip › Supplementary_S3/unknown1/spectrogram/180522_A11_08_wavchunk27_f_GMM_23.png]

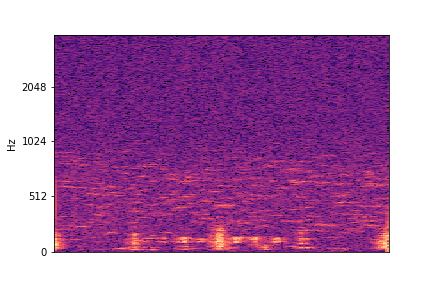

Supplement: Supplementary file 5 — Supplementary Audio [file 42003_2023_5700_MOESM5_ESM.zip › Supplementary_S3/unknown1/spectrogram/180522_A16_161_wavchunk29_f_GMM_115.png]

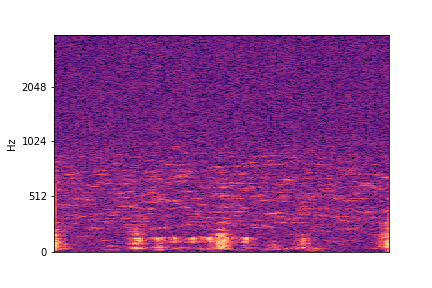

Supplement: Supplementary file 5 — Supplementary Audio [file 42003_2023_5700_MOESM5_ESM.zip › Supplementary_S3/unknown1/spectrogram/180522_A16_161_wavchunk29_f_GMM_126.png]

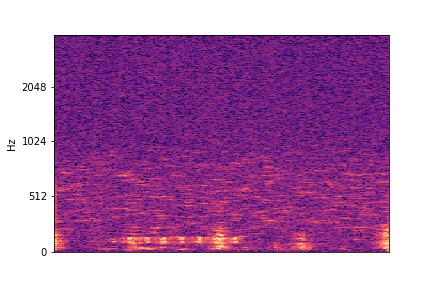

Supplement: Supplementary file 5 — Supplementary Audio [file 42003_2023_5700_MOESM5_ESM.zip › Supplementary_S3/unknown1/spectrogram/180522_A16_161_wavchunk29_f_GMM_141.png]

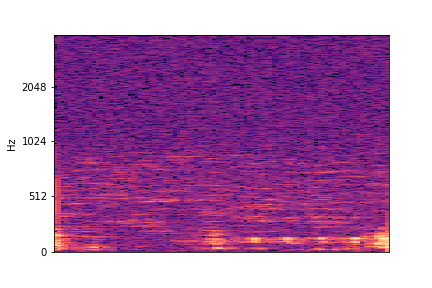

Supplement: Supplementary file 5 — Supplementary Audio [file 42003_2023_5700_MOESM5_ESM.zip › Supplementary_S3/unknown1/spectrogram/180522_A16_161_wavchunk29_f_GMM_147.png]

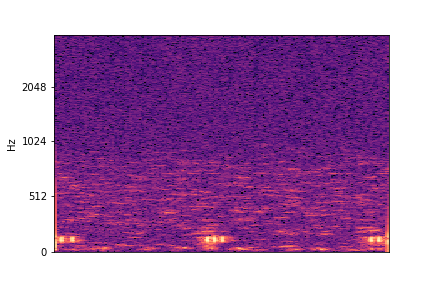

Supplement: Supplementary file 5 — Supplementary Audio [file 42003_2023_5700_MOESM5_ESM.zip › Supplementary_S3/unknown1/spectrogram/180522_A24_160_wavchunk29_f_GMM_43.png]

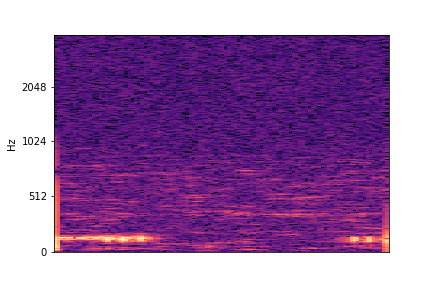

Supplement: Supplementary file 5 — Supplementary Audio [file 42003_2023_5700_MOESM5_ESM.zip › Supplementary_S3/unknown1/spectrogram/180522_A24_160_wavchunk29_f_GMM_49.png]

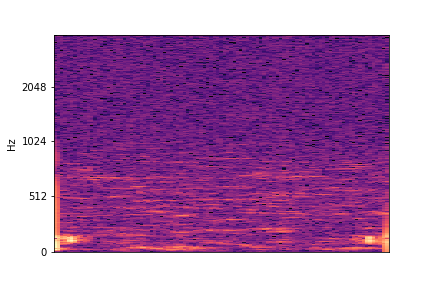

Supplement: Supplementary file 5 — Supplementary Audio [file 42003_2023_5700_MOESM5_ESM.zip › Supplementary_S3/unknown1/spectrogram/180522_A24_160_wavchunk33_f_GMM_2.png]

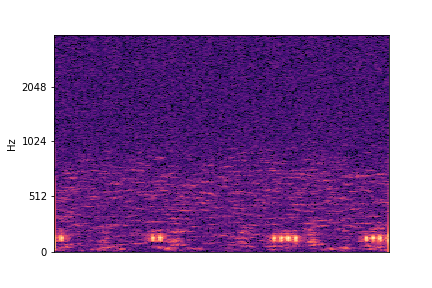

Supplement: Supplementary file 5 — Supplementary Audio [file 42003_2023_5700_MOESM5_ESM.zip › Supplementary_S3/unknown1/spectrogram/180522_A24_160_wavchunk39_f_GMM_28.png]

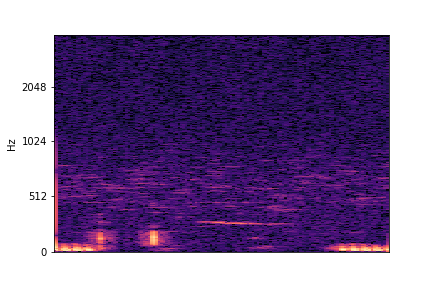

Supplement: Supplementary file 5 — Supplementary Audio [file 42003_2023_5700_MOESM5_ESM.zip › Supplementary_S3/unknown1/spectrogram/180522_A26_08_wavchunk23_f_GMM_1.png]

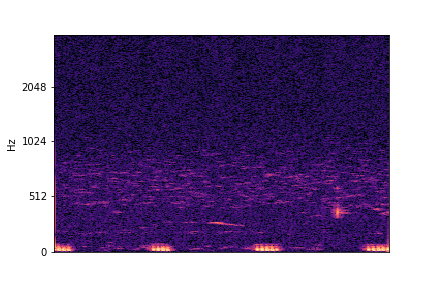

Supplement: Supplementary file 5 — Supplementary Audio [file 42003_2023_5700_MOESM5_ESM.zip › Supplementary_S3/unknown1/spectrogram/180522_A26_08_wavchunk23_f_GMM_3.png]

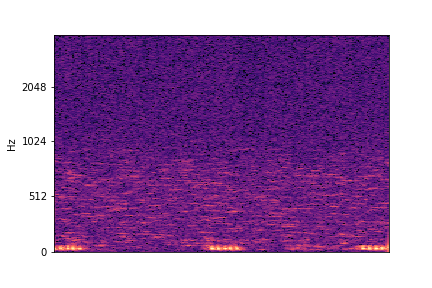

Supplement: Supplementary file 5 — Supplementary Audio [file 42003_2023_5700_MOESM5_ESM.zip › Supplementary_S3/unknown1/spectrogram/180522_A43_08_wavchunk48_f_GMM_25.png]

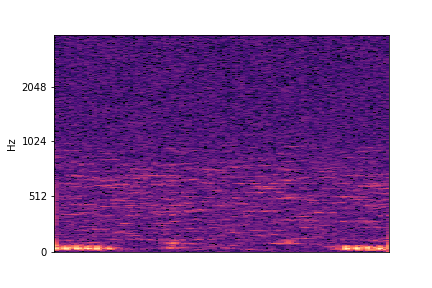

Supplement: Supplementary file 5 — Supplementary Audio [file 42003_2023_5700_MOESM5_ESM.zip › Supplementary_S3/unknown1/spectrogram/180522_A43_08_wavchunk48_f_GMM_26.png]

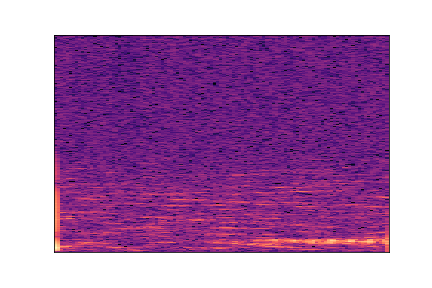

Supplement: Supplementary file 5 — Supplementary Audio [file 42003_2023_5700_MOESM5_ESM.zip › Supplementary_S3/unknown1/spectrogram/180522_B11_160_wavchunk37_f_GMM_16.png]

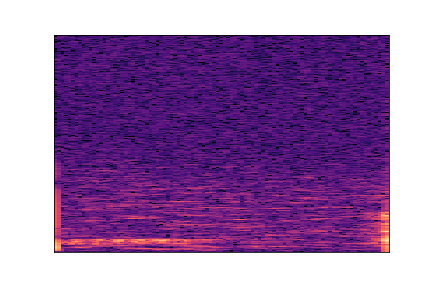

Supplement: Supplementary file 5 — Supplementary Audio [file 42003_2023_5700_MOESM5_ESM.zip › Supplementary_S3/unknown1/spectrogram/180522_B11_160_wavchunk37_f_GMM_17.png]

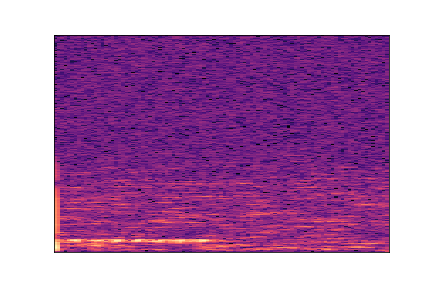

Supplement: Supplementary file 5 — Supplementary Audio [file 42003_2023_5700_MOESM5_ESM.zip › Supplementary_S3/unknown1/spectrogram/180522_B11_160_wavchunk37_f_GMM_20.png]

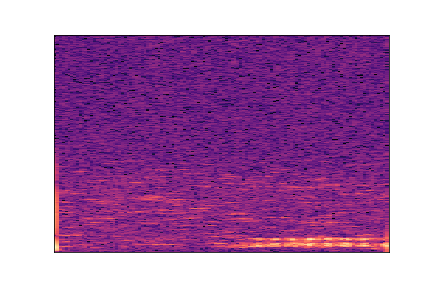

Supplement: Supplementary file 5 — Supplementary Audio [file 42003_2023_5700_MOESM5_ESM.zip › Supplementary_S3/unknown1/spectrogram/180522_B11_160_wavchunk38_f_GMM_12.png]

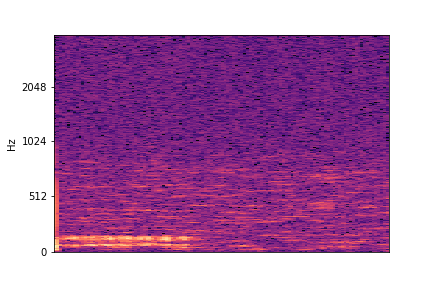

Supplement: Supplementary file 5 — Supplementary Audio [file 42003_2023_5700_MOESM5_ESM.zip › Supplementary_S3/unknown1/spectrogram/180522_B11_160_wavchunk44_f_GMM_29.png]

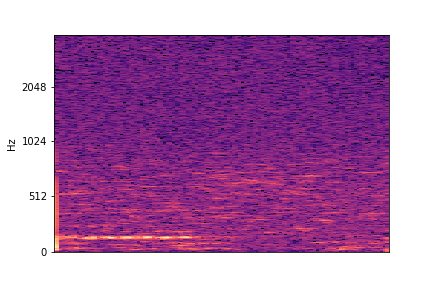

Supplement: Supplementary file 5 — Supplementary Audio [file 42003_2023_5700_MOESM5_ESM.zip › Supplementary_S3/unknown1/spectrogram/180522_B11_161_wavchunk12_f_GMM_33.png]

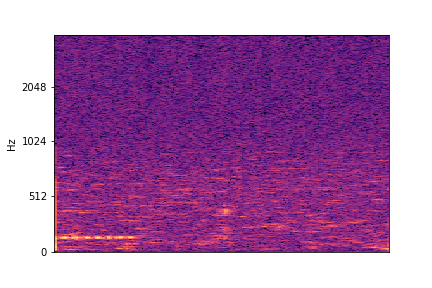

Supplement: Supplementary file 5 — Supplementary Audio [file 42003_2023_5700_MOESM5_ESM.zip › Supplementary_S3/unknown1/spectrogram/180522_B11_161_wavchunk12_f_GMM_35.png]

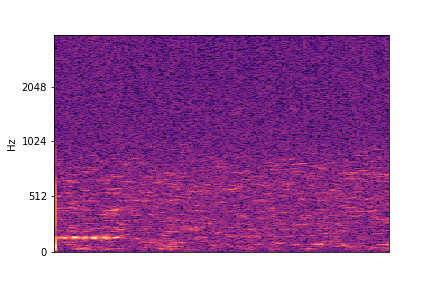

Supplement: Supplementary file 5 — Supplementary Audio [file 42003_2023_5700_MOESM5_ESM.zip › Supplementary_S3/unknown1/spectrogram/180522_B11_161_wavchunk12_f_GMM_36.png]

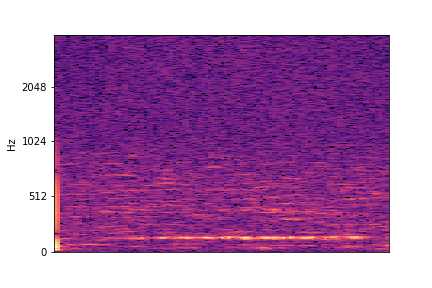

Supplement: Supplementary file 5 — Supplementary Audio [file 42003_2023_5700_MOESM5_ESM.zip › Supplementary_S3/unknown1/spectrogram/180522_B11_161_wavchunk12_f_GMM_45.png]

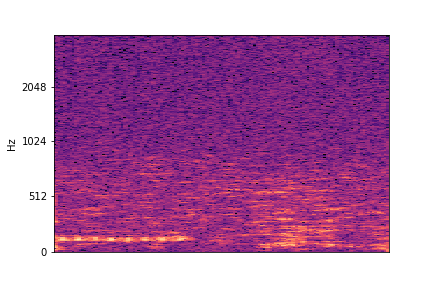

Supplement: Supplementary file 5 — Supplementary Audio [file 42003_2023_5700_MOESM5_ESM.zip › Supplementary_S3/unknown1/spectrogram/180522_B19_160_wavchunk22_f_GMM_2.png]

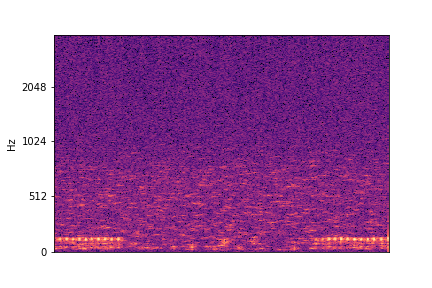

Supplement: Supplementary file 5 — Supplementary Audio [file 42003_2023_5700_MOESM5_ESM.zip › Supplementary_S3/unknown1/spectrogram/180522_B19_160_wavchunk24_f_GMM_107.png]

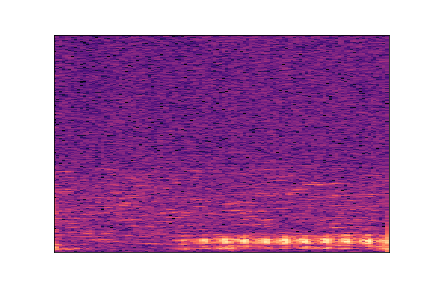

Supplement: Supplementary file 5 — Supplementary Audio [file 42003_2023_5700_MOESM5_ESM.zip › Supplementary_S3/unknown1/spectrogram/180522_B19_161_wavchunk4_f_GMM_46.png]

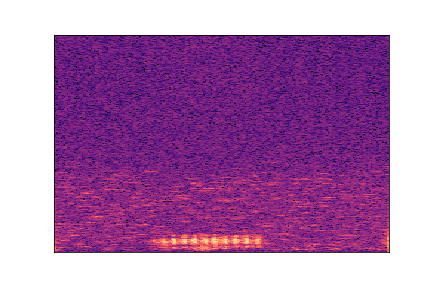

Supplement: Supplementary file 5 — Supplementary Audio [file 42003_2023_5700_MOESM5_ESM.zip › Supplementary_S3/unknown1/spectrogram/180522_B19_161_wavchunk5_f_GMM_3.png]

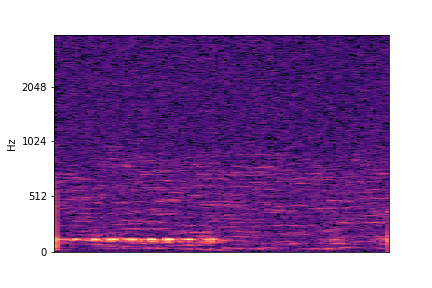

Supplement: Supplementary file 5 — Supplementary Audio [file 42003_2023_5700_MOESM5_ESM.zip › Supplementary_S3/unknown1/spectrogram/180522_B20_160_wavchunk21_f_GMM_27.png]

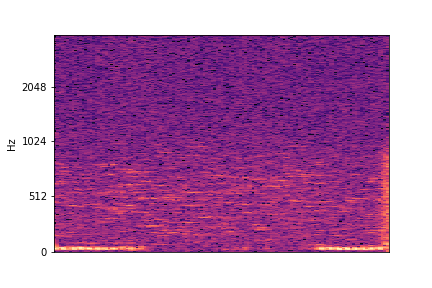

Supplement: Supplementary file 5 — Supplementary Audio [file 42003_2023_5700_MOESM5_ESM.zip › Supplementary_S3/unknown1/spectrogram/180527_C03_08_wavchunk23_f_GMM_158.png]

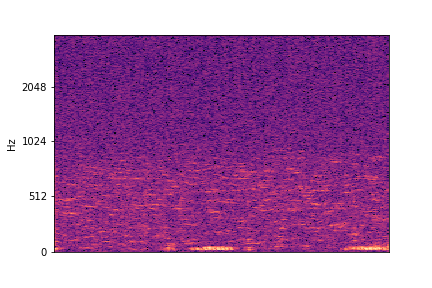

Supplement: Supplementary file 5 — Supplementary Audio [file 42003_2023_5700_MOESM5_ESM.zip › Supplementary_S3/unknown1/spectrogram/180527_C03_08_wavchunk24_f_GMM_1.png]

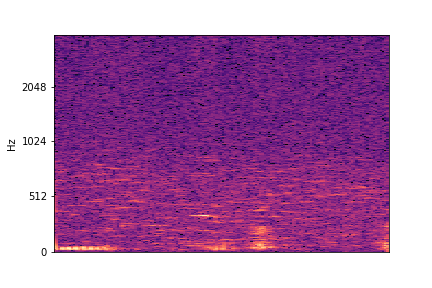

Supplement: Supplementary file 5 — Supplementary Audio [file 42003_2023_5700_MOESM5_ESM.zip › Supplementary_S3/unknown1/spectrogram/180527_C03_08_wavchunk24_f_GMM_2.png]

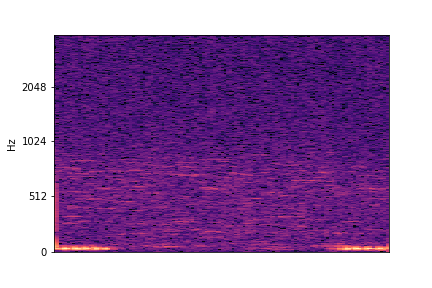

Supplement: Supplementary file 5 — Supplementary Audio [file 42003_2023_5700_MOESM5_ESM.zip › Supplementary_S3/unknown1/spectrogram/180527_C03_08_wavchunk49_f_GMM_20.png]

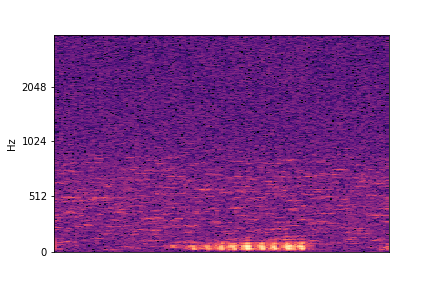

Supplement: Supplementary file 5 — Supplementary Audio [file 42003_2023_5700_MOESM5_ESM.zip › Supplementary_S3/unknown1/spectrogram/180527_C03_16_1_wavchunk55_f_GMM_40.png]

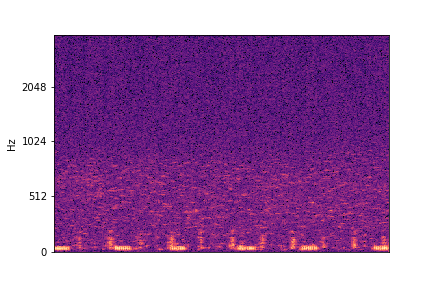

Supplement: Supplementary file 5 — Supplementary Audio [file 42003_2023_5700_MOESM5_ESM.zip › Supplementary_S3/unknown1/spectrogram/180527_C03_16_1_wavchunk8_f_GMM_82.png]

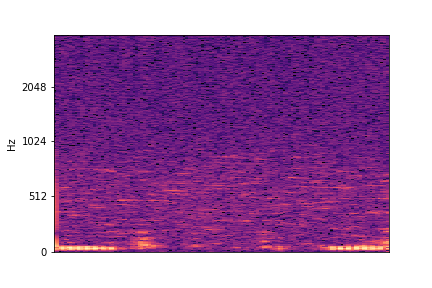

Supplement: Supplementary file 5 — Supplementary Audio [file 42003_2023_5700_MOESM5_ESM.zip › Supplementary_S3/unknown1/spectrogram/180527_C03_16_1_wavchunk8_f_GMM_83.png]

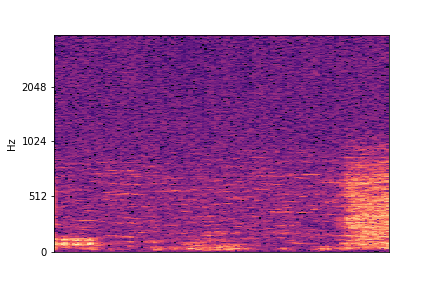

Supplement: Supplementary file 5 — Supplementary Audio [file 42003_2023_5700_MOESM5_ESM.zip › Supplementary_S3/unknown1/spectrogram/180527_C05_16_1_wavchunk13_f_GMM_3.png]

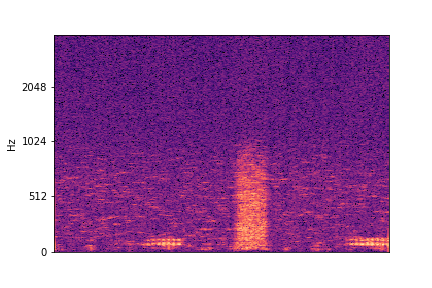

Supplement: Supplementary file 5 — Supplementary Audio [file 42003_2023_5700_MOESM5_ESM.zip › Supplementary_S3/unknown1/spectrogram/180527_C05_16_1_wavchunk13_f_GMM_6.png]

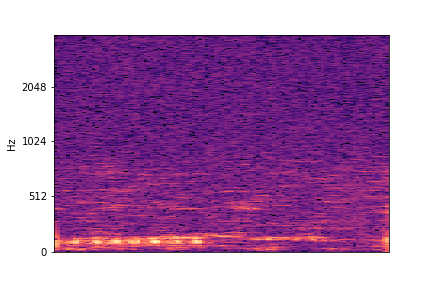

Supplement: Supplementary file 5 — Supplementary Audio [file 42003_2023_5700_MOESM5_ESM.zip › Supplementary_S3/unknown1/spectrogram/180527_C07_16_1_wavchunk48_f_GMM_62.png]

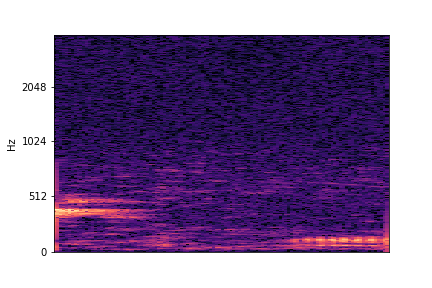

Supplement: Supplementary file 5 — Supplementary Audio [file 42003_2023_5700_MOESM5_ESM.zip › Supplementary_S3/unknown1/spectrogram/180527_C09_08_wavchunk48_f_GMM_55.png]

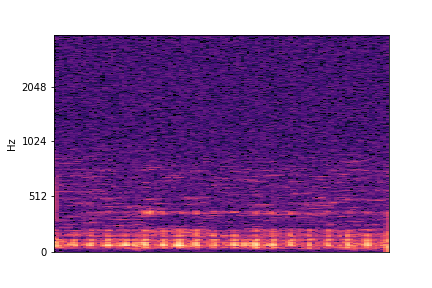

Supplement: Supplementary file 5 — Supplementary Audio [file 42003_2023_5700_MOESM5_ESM.zip › Supplementary_S3/unknown1/spectrogram/180527_C11_16_1_wavchunk28_f_GMM_41.png]

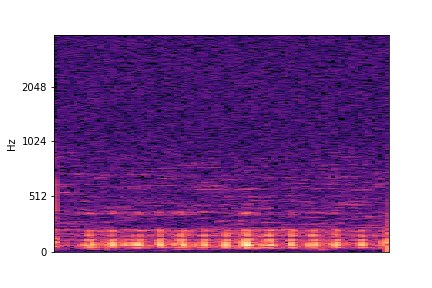

Supplement: Supplementary file 5 — Supplementary Audio [file 42003_2023_5700_MOESM5_ESM.zip › Supplementary_S3/unknown1/spectrogram/180527_C11_16_1_wavchunk28_f_GMM_42.png]

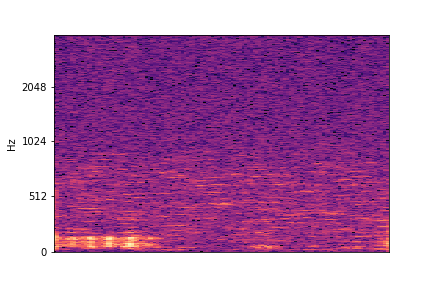

Supplement: Supplementary file 5 — Supplementary Audio [file 42003_2023_5700_MOESM5_ESM.zip › Supplementary_S3/unknown1/spectrogram/180527_C11_16_1_wavchunk42_f_GMM_32.png]

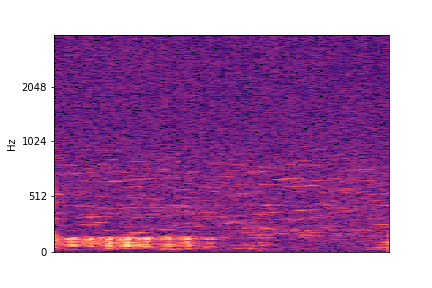

Supplement: Supplementary file 5 — Supplementary Audio [file 42003_2023_5700_MOESM5_ESM.zip › Supplementary_S3/unknown1/spectrogram/180527_C11_16_1_wavchunk42_f_GMM_50.png]

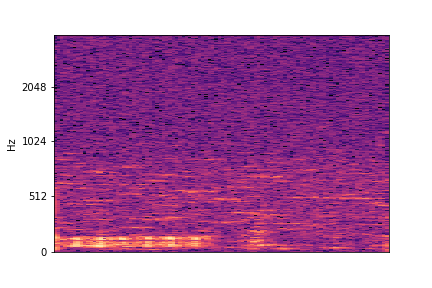

Supplement: Supplementary file 5 — Supplementary Audio [file 42003_2023_5700_MOESM5_ESM.zip › Supplementary_S3/unknown1/spectrogram/180527_C11_16_1_wavchunk43_f_GMM_26.png]

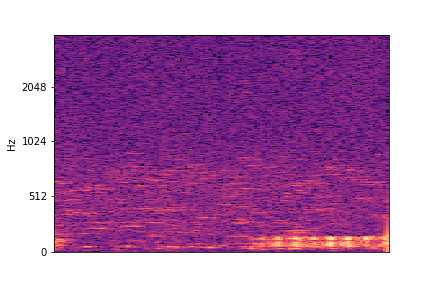

Supplement: Supplementary file 5 — Supplementary Audio [file 42003_2023_5700_MOESM5_ESM.zip › Supplementary_S3/unknown1/spectrogram/180527_C11_16_1_wavchunk43_f_GMM_36.png]

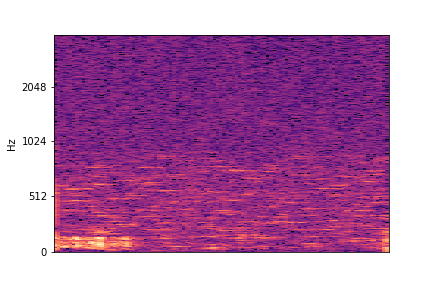

Supplement: Supplementary file 5 — Supplementary Audio [file 42003_2023_5700_MOESM5_ESM.zip › Supplementary_S3/unknown1/spectrogram/180527_C11_16_1_wavchunk43_f_GMM_4.png]

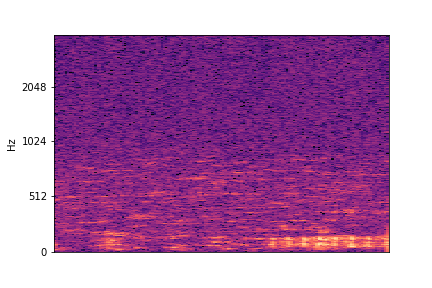

Supplement: Supplementary file 5 — Supplementary Audio [file 42003_2023_5700_MOESM5_ESM.zip › Supplementary_S3/unknown1/spectrogram/180527_C11_16_1_wavchunk43_f_GMM_5.png]

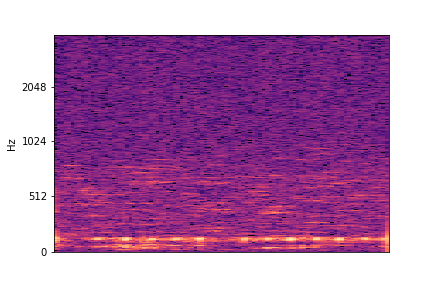

Supplement: Supplementary file 5 — Supplementary Audio [file 42003_2023_5700_MOESM5_ESM.zip › Supplementary_S3/unknown1/spectrogram/180527_C11_16_2_wavchunk13_f_GMM_4.png]

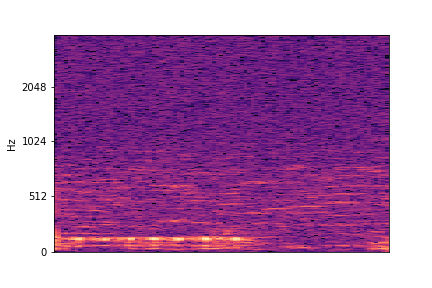

Supplement: Supplementary file 5 — Supplementary Audio [file 42003_2023_5700_MOESM5_ESM.zip › Supplementary_S3/unknown1/spectrogram/180527_C11_16_2_wavchunk13_f_GMM_83.png]

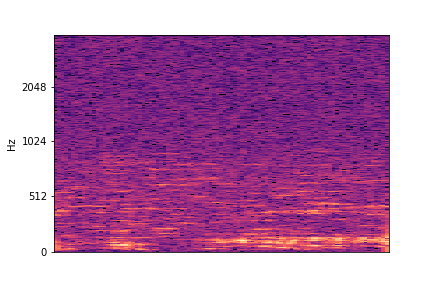

Supplement: Supplementary file 5 — Supplementary Audio [file 42003_2023_5700_MOESM5_ESM.zip › Supplementary_S3/unknown1/spectrogram/180527_C11_16_2_wavchunk2_f_GMM_43.png]

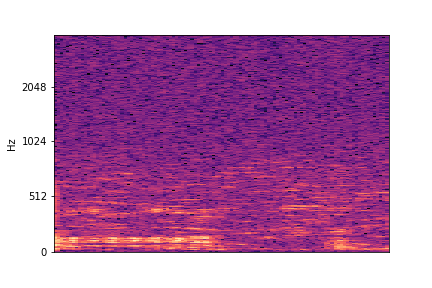

Supplement: Supplementary file 5 — Supplementary Audio [file 42003_2023_5700_MOESM5_ESM.zip › Supplementary_S3/unknown1/spectrogram/180527_C11_16_2_wavchunk2_f_GMM_44.png]

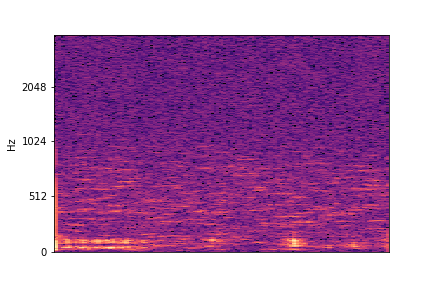

Supplement: Supplementary file 5 — Supplementary Audio [file 42003_2023_5700_MOESM5_ESM.zip › Supplementary_S3/unknown1/spectrogram/180527_C14_16_1_wavchunk54_f_GMM_32.png]

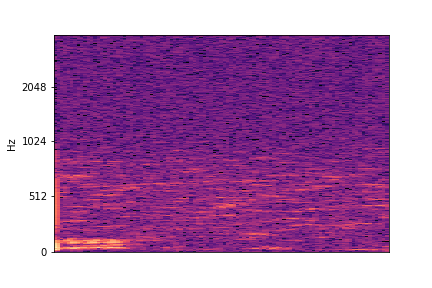

Supplement: Supplementary file 5 — Supplementary Audio [file 42003_2023_5700_MOESM5_ESM.zip › Supplementary_S3/unknown1/spectrogram/180527_C14_16_1_wavchunk54_f_GMM_68.png]

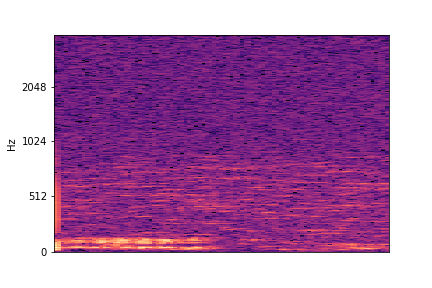

Supplement: Supplementary file 5 — Supplementary Audio [file 42003_2023_5700_MOESM5_ESM.zip › Supplementary_S3/unknown1/spectrogram/180527_C14_16_2_wavchunk0_f_GMM_21.png]

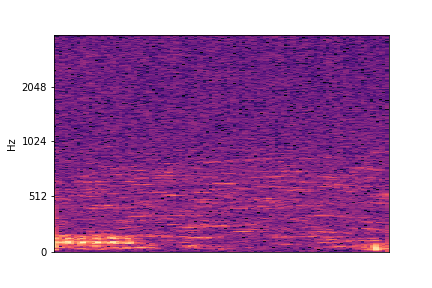

Supplement: Supplementary file 5 — Supplementary Audio [file 42003_2023_5700_MOESM5_ESM.zip › Supplementary_S3/unknown1/spectrogram/180527_C18_08_wavchunk39_f_GMM_7.png]

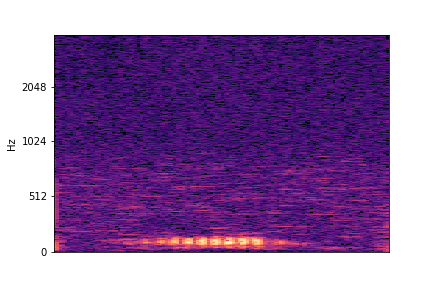

Supplement: Supplementary file 5 — Supplementary Audio [file 42003_2023_5700_MOESM5_ESM.zip › Supplementary_S3/unknown1/spectrogram/180527_C18_08_wavchunk44_f_GMM_46.png]

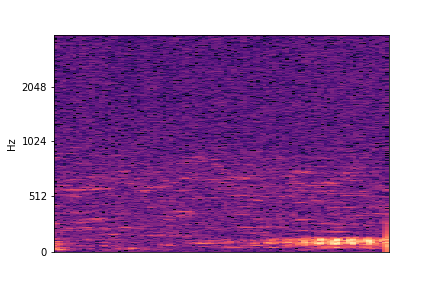

Supplement: Supplementary file 5 — Supplementary Audio [file 42003_2023_5700_MOESM5_ESM.zip › Supplementary_S3/unknown1/spectrogram/180527_C18_08_wavchunk44_f_GMM_48.png]

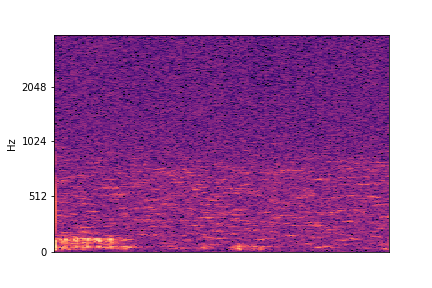

Supplement: Supplementary file 5 — Supplementary Audio [file 42003_2023_5700_MOESM5_ESM.zip › Supplementary_S3/unknown1/spectrogram/180527_C18_16_1_wavchunk45_f_GMM_14.png]

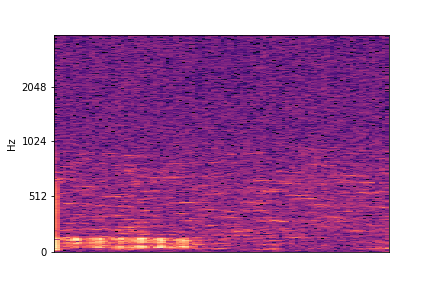

Supplement: Supplementary file 5 — Supplementary Audio [file 42003_2023_5700_MOESM5_ESM.zip › Supplementary_S3/unknown1/spectrogram/180527_C18_16_1_wavchunk45_f_GMM_7.png]

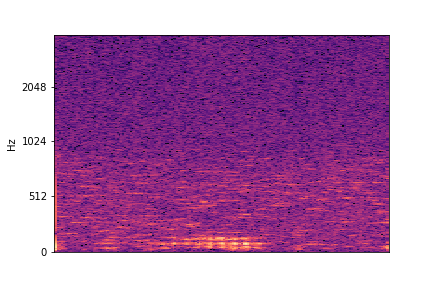

Supplement: Supplementary file 5 — Supplementary Audio [file 42003_2023_5700_MOESM5_ESM.zip › Supplementary_S3/unknown1/spectrogram/180527_C18_16_1_wavchunk46_f_GMM_58.png]

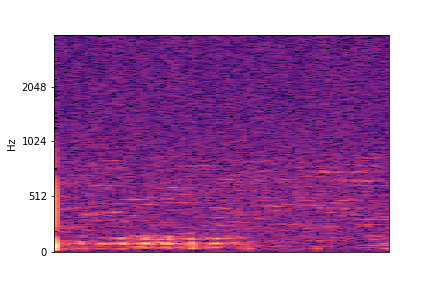

Supplement: Supplementary file 5 — Supplementary Audio [file 42003_2023_5700_MOESM5_ESM.zip › Supplementary_S3/unknown1/spectrogram/180527_C18_16_1_wavchunk46_f_GMM_66.png]

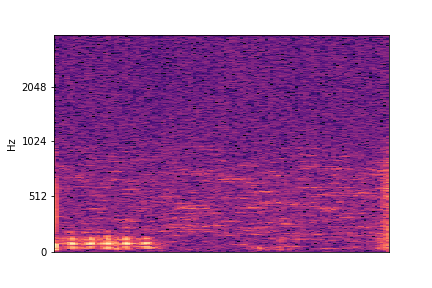

Supplement: Supplementary file 5 — Supplementary Audio [file 42003_2023_5700_MOESM5_ESM.zip › Supplementary_S3/unknown1/spectrogram/180527_C18_16_1_wavchunk46_f_GMM_81.png]

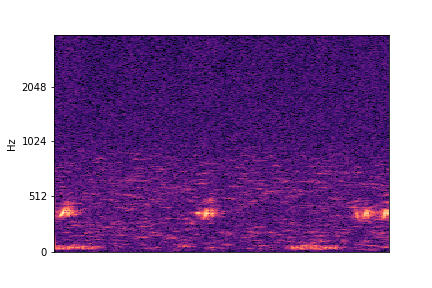

Supplement: Supplementary file 5 — Supplementary Audio [file 42003_2023_5700_MOESM5_ESM.zip › Supplementary_S3/unknown1/spectrogram/180527_C19_08_wavchunk16_f_GMM_55.png]

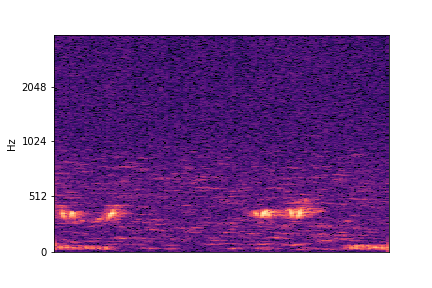

Supplement: Supplementary file 5 — Supplementary Audio [file 42003_2023_5700_MOESM5_ESM.zip › Supplementary_S3/unknown1/spectrogram/180527_C19_08_wavchunk16_f_GMM_57.png]

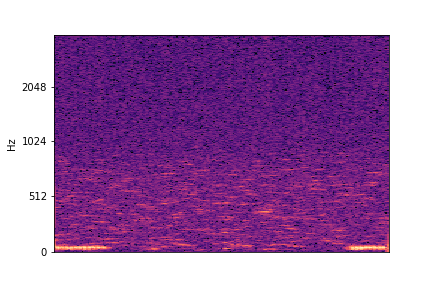

Supplement: Supplementary file 5 — Supplementary Audio [file 42003_2023_5700_MOESM5_ESM.zip › Supplementary_S3/unknown1/spectrogram/180527_C19_08_wavchunk46_f_GMM_15.png]

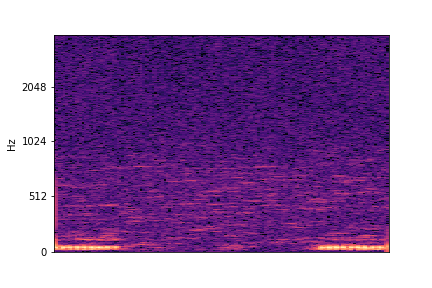

Supplement: Supplementary file 5 — Supplementary Audio [file 42003_2023_5700_MOESM5_ESM.zip › Supplementary_S3/unknown1/spectrogram/180527_C19_08_wavchunk46_f_GMM_21.png]

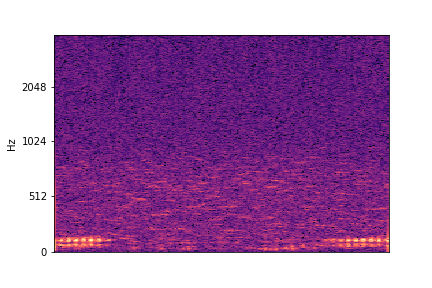

Supplement: Supplementary file 5 — Supplementary Audio [file 42003_2023_5700_MOESM5_ESM.zip › Supplementary_S3/unknown1/spectrogram/180527_C23_08_wavchunk39_f_GMM_62.png]

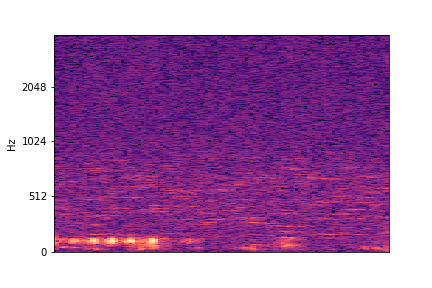

Supplement: Supplementary file 5 — Supplementary Audio [file 42003_2023_5700_MOESM5_ESM.zip › Supplementary_S3/unknown1/spectrogram/180527_C23_16_2_wavchunk0_f_GMM_62.png]

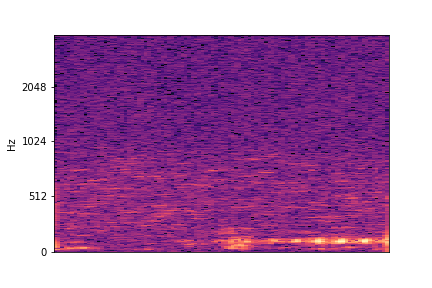

Supplement: Supplementary file 5 — Supplementary Audio [file 42003_2023_5700_MOESM5_ESM.zip › Supplementary_S3/unknown1/spectrogram/180527_C23_16_2_wavchunk0_f_GMM_65.png]

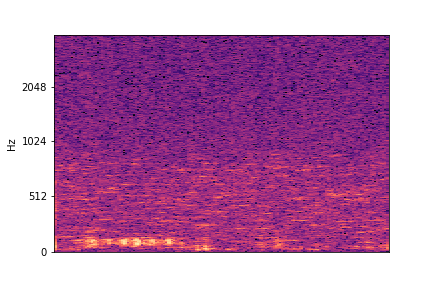

Supplement: Supplementary file 5 — Supplementary Audio [file 42003_2023_5700_MOESM5_ESM.zip › Supplementary_S3/unknown1/spectrogram/180527_C23_16_2_wavchunk14_f_GMM_150.png]

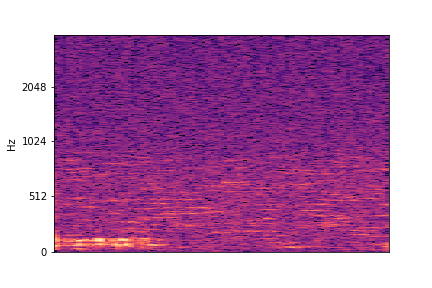

Supplement: Supplementary file 5 — Supplementary Audio [file 42003_2023_5700_MOESM5_ESM.zip › Supplementary_S3/unknown1/spectrogram/180527_C23_16_2_wavchunk23_f_GMM_106.png]

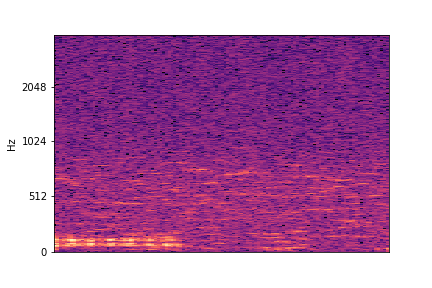

Supplement: Supplementary file 5 — Supplementary Audio [file 42003_2023_5700_MOESM5_ESM.zip › Supplementary_S3/unknown1/spectrogram/180527_C23_16_2_wavchunk23_f_GMM_113.png]

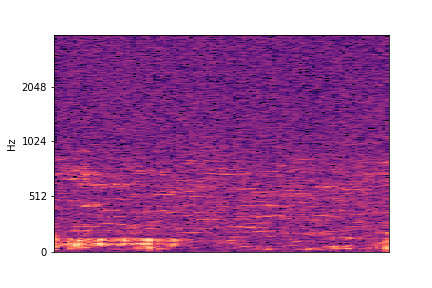

Supplement: Supplementary file 5 — Supplementary Audio [file 42003_2023_5700_MOESM5_ESM.zip › Supplementary_S3/unknown1/spectrogram/180527_C23_16_2_wavchunk23_f_GMM_89.png]

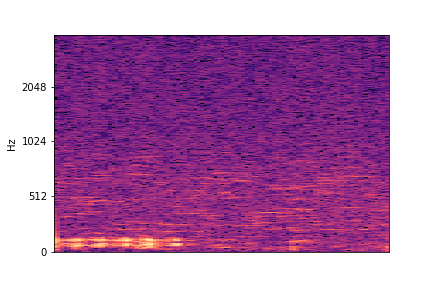

Supplement: Supplementary file 5 — Supplementary Audio [file 42003_2023_5700_MOESM5_ESM.zip › Supplementary_S3/unknown1/spectrogram/180527_C23_16_2_wavchunk23_f_GMM_90.png]

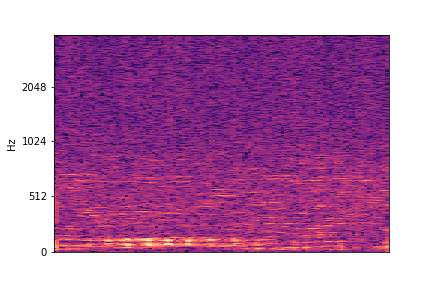

Supplement: Supplementary file 5 — Supplementary Audio [file 42003_2023_5700_MOESM5_ESM.zip › Supplementary_S3/unknown1/spectrogram/180527_C23_16_2_wavchunk23_f_GMM_97.png]

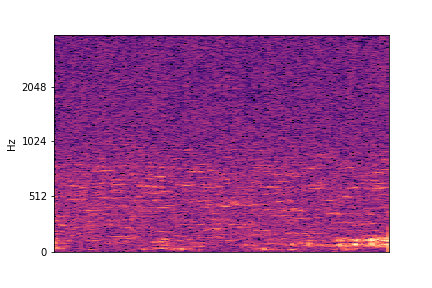

Supplement: Supplementary file 5 — Supplementary Audio [file 42003_2023_5700_MOESM5_ESM.zip › Supplementary_S3/unknown1/spectrogram/180527_C23_16_2_wavchunk23_f_GMM_98.png]

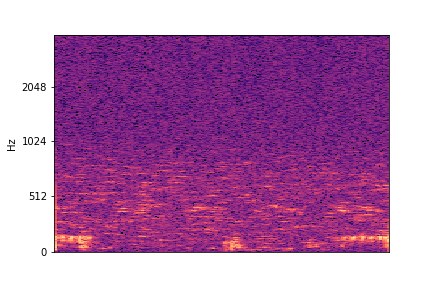

Supplement: Supplementary file 5 — Supplementary Audio [file 42003_2023_5700_MOESM5_ESM.zip › Supplementary_S3/unknown1/spectrogram/180703_B03_161_wavchunk5_f_GMM_101.png]

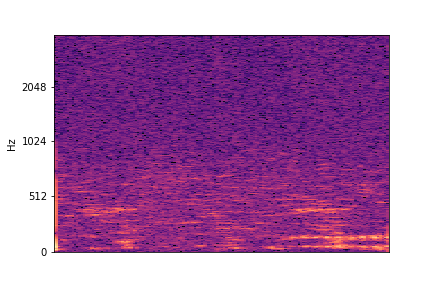

Supplement: Supplementary file 5 — Supplementary Audio [file 42003_2023_5700_MOESM5_ESM.zip › Supplementary_S3/unknown1/spectrogram/180703_B03_161_wavchunk5_f_GMM_82.png]

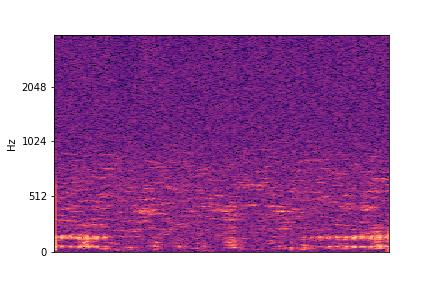

Supplement: Supplementary file 5 — Supplementary Audio [file 42003_2023_5700_MOESM5_ESM.zip › Supplementary_S3/unknown1/spectrogram/180703_B03_161_wavchunk5_f_GMM_88.png]

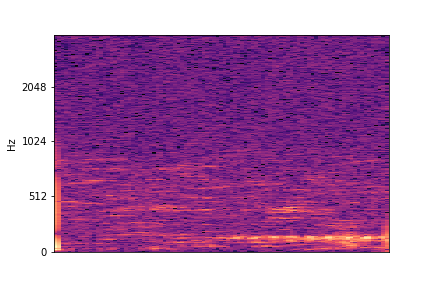

Supplement: Supplementary file 5 — Supplementary Audio [file 42003_2023_5700_MOESM5_ESM.zip › Supplementary_S3/unknown1/spectrogram/180703_B03_161_wavchunk5_f_GMM_99.png]

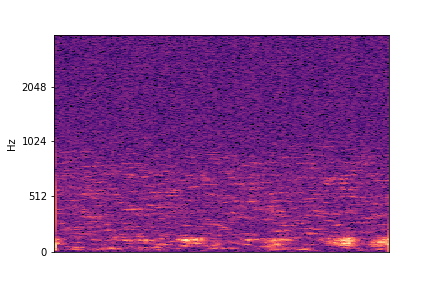

Supplement: Supplementary file 5 — Supplementary Audio [file 42003_2023_5700_MOESM5_ESM.zip › Supplementary_S3/unknown1/spectrogram/180703_B09_160_wavchunk3_f_GMM_4.png]

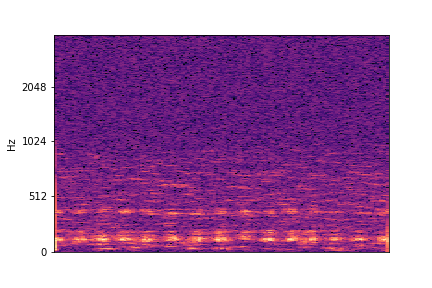

Supplement: Supplementary file 5 — Supplementary Audio [file 42003_2023_5700_MOESM5_ESM.zip › Supplementary_S3/unknown1/spectrogram/180703_B09_161_wavchunk11_f_GMM_18.png]

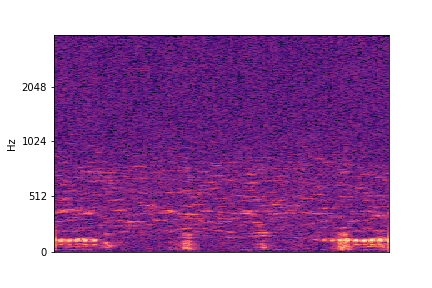

Supplement: Supplementary file 5 — Supplementary Audio [file 42003_2023_5700_MOESM5_ESM.zip › Supplementary_S3/unknown1/spectrogram/180703_B25_160_wavchunk31_f_GMM_95.png]

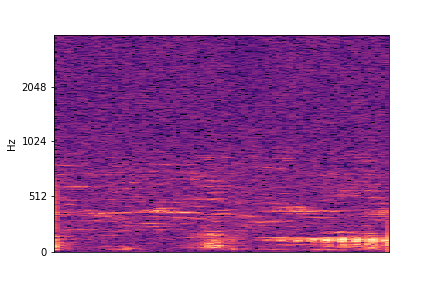

Supplement: Supplementary file 5 — Supplementary Audio [file 42003_2023_5700_MOESM5_ESM.zip › Supplementary_S3/unknown1/spectrogram/180703_B25_160_wavchunk32_f_GMM_20.png]

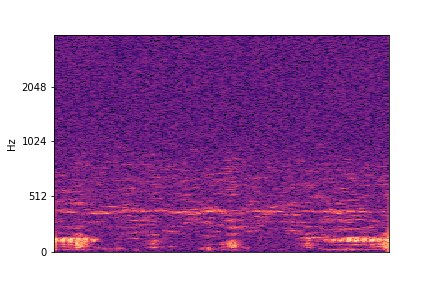

Supplement: Supplementary file 5 — Supplementary Audio [file 42003_2023_5700_MOESM5_ESM.zip › Supplementary_S3/unknown1/spectrogram/180703_B25_160_wavchunk32_f_GMM_36.png]

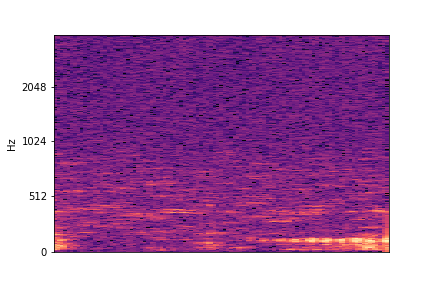

Supplement: Supplementary file 5 — Supplementary Audio [file 42003_2023_5700_MOESM5_ESM.zip › Supplementary_S3/unknown1/spectrogram/180703_B25_160_wavchunk32_f_GMM_63.png]

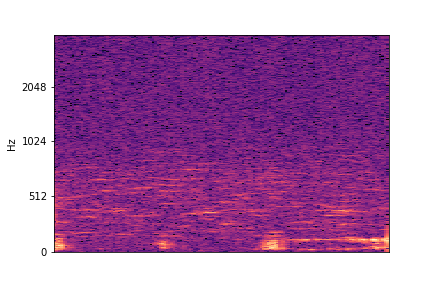

Supplement: Supplementary file 5 — Supplementary Audio [file 42003_2023_5700_MOESM5_ESM.zip › Supplementary_S3/unknown1/spectrogram/180703_B25_161_wavchunk11_f_GMM_122.png]

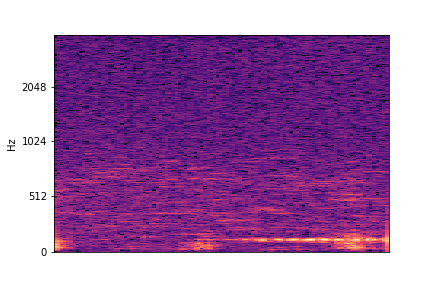

Supplement: Supplementary file 5 — Supplementary Audio [file 42003_2023_5700_MOESM5_ESM.zip › Supplementary_S3/unknown1/spectrogram/180703_B25_161_wavchunk11_f_GMM_123.png]

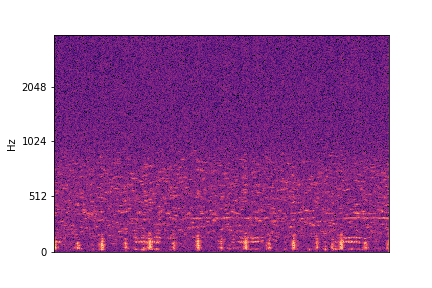

Supplement: Supplementary file 5 — Supplementary Audio [file 42003_2023_5700_MOESM5_ESM.zip › Supplementary_S3/unknown1/spectrogram/180703_B25_161_wavchunk12_f_GMM_16.png]

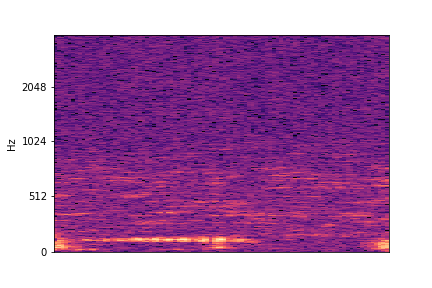

Supplement: Supplementary file 5 — Supplementary Audio [file 42003_2023_5700_MOESM5_ESM.zip › Supplementary_S3/unknown1/spectrogram/180703_B25_161_wavchunk12_f_GMM_21.png]

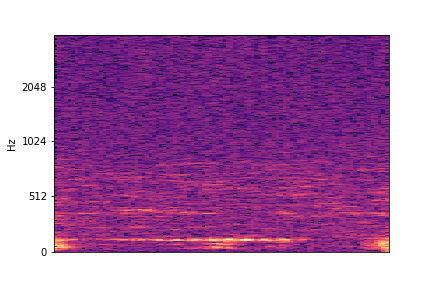

Supplement: Supplementary file 5 — Supplementary Audio [file 42003_2023_5700_MOESM5_ESM.zip › Supplementary_S3/unknown1/spectrogram/180703_B25_161_wavchunk12_f_GMM_31.png]

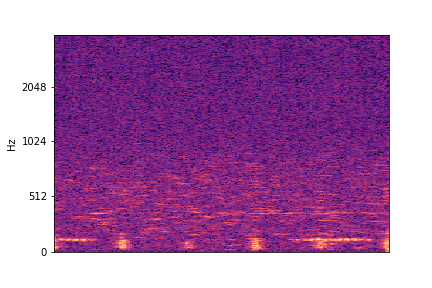

Supplement: Supplementary file 5 — Supplementary Audio [file 42003_2023_5700_MOESM5_ESM.zip › Supplementary_S3/unknown1/spectrogram/180703_B25_161_wavchunk12_f_GMM_33.png]

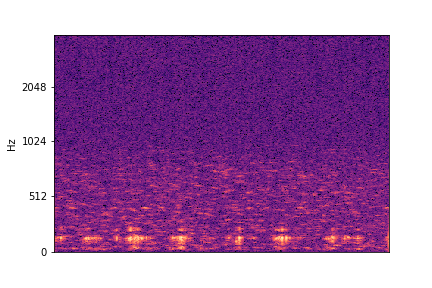

Supplement: Supplementary file 5 — Supplementary Audio [file 42003_2023_5700_MOESM5_ESM.zip › Supplementary_S3/unknown2/spectrogram/180520_D02_08_2_wavchunk36_f_GMM_10.png]

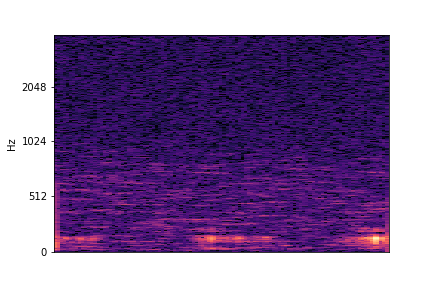

Supplement: Supplementary file 5 — Supplementary Audio [file 42003_2023_5700_MOESM5_ESM.zip › Supplementary_S3/unknown2/spectrogram/180520_D02_08_2_wavchunk36_f_GMM_11.png]

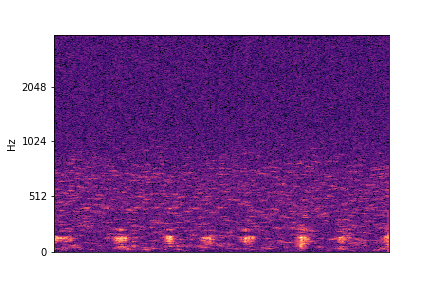

Supplement: Supplementary file 5 — Supplementary Audio [file 42003_2023_5700_MOESM5_ESM.zip › Supplementary_S3/unknown2/spectrogram/180520_D02_08_2_wavchunk36_f_GMM_13.png]

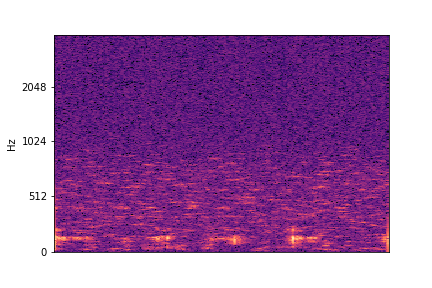

Supplement: Supplementary file 5 — Supplementary Audio [file 42003_2023_5700_MOESM5_ESM.zip › Supplementary_S3/unknown2/spectrogram/180520_D02_08_2_wavchunk36_f_GMM_5.png]

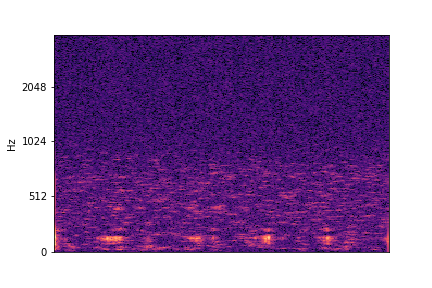

Supplement: Supplementary file 5 — Supplementary Audio [file 42003_2023_5700_MOESM5_ESM.zip › Supplementary_S3/unknown2/spectrogram/180520_D02_08_2_wavchunk36_f_GMM_6.png]

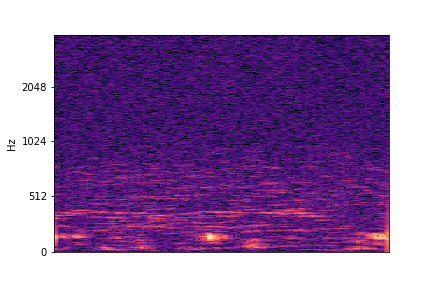

Supplement: Supplementary file 5 — Supplementary Audio [file 42003_2023_5700_MOESM5_ESM.zip › Supplementary_S3/unknown2/spectrogram/180520_D05_08_2_wavchunk30_f_GMM_66.png]

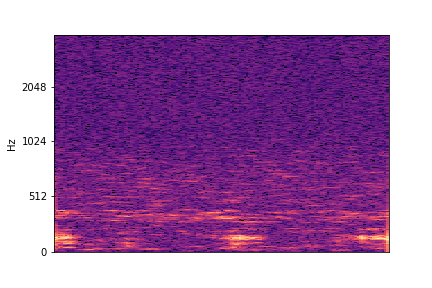

Supplement: Supplementary file 5 — Supplementary Audio [file 42003_2023_5700_MOESM5_ESM.zip › Supplementary_S3/unknown2/spectrogram/180520_D05_08_2_wavchunk30_f_GMM_74.png]

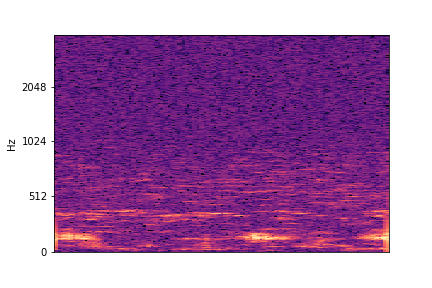

Supplement: Supplementary file 5 — Supplementary Audio [file 42003_2023_5700_MOESM5_ESM.zip › Supplementary_S3/unknown2/spectrogram/180520_D05_08_2_wavchunk30_f_GMM_75.png]
