# Supplementary material for: Vibroscape analysis reveals acoustic niche overlap and plastic alteration of vibratory courtship signals in ground-dwelling wolf spiders
Source: Commun Biol. 2024 Jan 5;7:23. doi: 10.1038/s42003-023-05700-6 (PMC10770364; doi:10.1038/s42003-023-05700-6)
Supplement: Supplementary file 7 — Reporting Summary [file 42003_2023_5700_MOESM7_ESM.pdf]

nature portfolio

Corresponding author(s): Noori Choi

Last updated by author(s): Dec 1, 2023

Reporting Summary

Nature Portfolio wishes to improve the reproducibility of the work that we publish. This form provides structure for consistency and transparency in reporting. For further information on Nature Portfolio policies, see our Editorial Policies and the Editorial Policy Checklist.

Statistics

For all statistical analyses, confirm that the following items are present in the figure legend, table legend, main text, or Methods section.

n/a	Confirmed	
☐	☒	The exact sample size (*n*) for each experimental group/condition, given as a discrete number and unit of measurement
☐	☒	A statement on whether measurements were taken from distinct samples or whether the same sample was measured repeatedly
☐	☒	The statistical test(s) used AND whether they are one- or two-sided<br>*Only common tests should be described solely by name; describe more complex techniques in the Methods section.*
☐	☒	A description of all covariates tested
☐	☒	A description of any assumptions or corrections, such as tests of normality and adjustment for multiple comparisons
☐	☒	A full description of the statistical parameters including central tendency (e.g. means) or other basic estimates (e.g. regression coefficient) AND variation (e.g. standard deviation) or associated estimates of uncertainty (e.g. confidence intervals)
☐	☒	For null hypothesis testing, the test statistic (e.g. *F*, *t*, *r*) with confidence intervals, effect sizes, degrees of freedom and *P* value noted<br>*Give P values as exact values whenever suitable.*
☒	☐	For Bayesian analysis, information on the choice of priors and Markov chain Monte Carlo settings
☒	☐	For hierarchical and complex designs, identification of the appropriate level for tests and full reporting of outcomes
☒	☐	Estimates of effect sizes (e.g. Cohen's *d*, Pearson's *r*), indicating how they were calculated

Our web collection on statistics for biologists contains articles on many of the points above.

Software and code

Policy information about availability of computer code

Data collection	No software were used for data collection.
Data analysis	We used a custom Python and R codes for audio filtering, sound detection, and statistical analyses. All the codes is available on Dryad repository (https://doi.org/10.5061/dryad.0gb5mkm5w) or in provided supplementary material (Supplementary_S4).

For manuscripts utilizing custom algorithms or software that are central to the research but not yet described in published literature, software must be made available to editors and reviewers. We strongly encourage code deposition in a community repository (e.g. GitHub). See the Nature Portfolio guidelines for submitting code & software for further information.

Data

Policy information about availability of data

All manuscripts must include a data availability statement. This statement should provide the following information, where applicable:

- Accession codes, unique identifiers, or web links for publicly available datasets
- A description of any restrictions on data availability
- For clinical datasets or third party data, please ensure that the statement adheres to our policy

All codes and files to replicate the results are available on Dryad repository (https://doi.org/10.5061/dryad.0gb5mkm5w) or in provided supplemental materials. Due to the large size of the audio dataset, raw audio files cannot be provided through general public repositories, but authors can provide the files upon request.

Human research participants

Policy information about studies involving human research participants and Sex and Gender in Research.

Reporting on sex and gender	No data collected from human research participants.
Population characteristics	See above
Recruitment	See above
Ethics oversight	See above

Note that full information on the approval of the study protocol must also be provided in the manuscript.

Field-specific reporting

Please select the one below that is the best fit for your research. If you are not sure, read the appropriate sections before making your selection.

☐ Life sciences ☐ Behavioural & social sciences ☒ Ecological, evolutionary & environmental sciences

For a reference copy of the document with all sections, see nature.com/documents/nr-reporting-summary-flat.pdf

Ecological, evolutionary & environmental sciences study design

All studies must disclose on these points even when the disclosure is negative.

Study description	We chose five study plots (10 m x 10 m) at the field station of the University of Mississippi at Abbeville, Mississippi, USA (34°43’ N 89°39’ W). We chose study plot locations based on a preliminary survey of species diversity by direct observation. To encompass the variation in species composition associated with substrate types, we chose to focus on two distinct microhabitats – (i) leaf litter (n=3) and (ii) pine litter (n=2).
Research sample	We conducted audio recording and pitfall trapping at the field station of the University of Mississippi. We collected sounds and specimens of ground-dwelling arthropods in our study plots (10 x 10 m).
Sampling strategy	Using the field recording experiment at the field station of the University of Mississippi, we collected 17,713 signal bouts from 73 different types of vibratory sounds. We did not calculate the sample size, but the size of dataset is large enough as compared to the previous studies.
Data collection	- Audio data was recorded by passive recording setup on forest floor. In each study plot, we deployed a TemLog20 temperature logger (Tamtop, Milpitas, California, USA), 25 recording units consisting of a contact microphone (35 mm diameter, Goedrum Co., Chanhua, Taiwan) and a Toobom R01 8GB acoustic recorder (Toobom, China), and four pitfall traps (Carolina biological supply company, Bunington, North Carolina, USA) (Figure 1b, 1c). The temperature loggers recorded the temperature at each recording plot every 15 minutes during the experimental periods. In total, we deployed 125 recording units, 5 temperature loggers, and 20 pitfall traps across our five study plots.<br>- We used propylene glycol for pitfall traps to minimize the potential environmental toxicity. We sorted collected specimens by the time of collection, collection date, and study plot and we preserved them in 95% ethanol for later species identification. We used the collected specimens to corroborate our species identity of sound recordings across locations.
Timing and spatial scale	We conducted a 24-hour recording every three days from May 15th to July 15th, 2018 resulting in a total of 1950 24-hour recordings across 13 days. The substrate-borne vibrations during 24 hours in study plots were continuously recorded from 0800 except 10 minutes to replace audio recorders at 1600 due to the limited battery capacity. We chose five study plots (10 m x 10 m) at the field station of the University of Mississippi at Abbeville, Mississippi, USA (34°43’ N 89°39’ W). We chose study plot locations based on a preliminary survey of species diversity by direct observation. On the same day, we collected specimens from pitfall traps at three different times (0800, 1600, and 0000) to observe the temporal variation in the activity of species in study plots.
Data exclusions	Among 17,713 signal bouts from 73 different types of vibratory sounds, we only used 17 sound types that occurred more than 100 times across recording periods for further analysis to focus on more commonly observed sound types.
Reproducibility	We used passive audio recording and pitfall trapping, so the observation may not be reproducible due to the random ecological and environmental factors. We provided all the details about our recording sites and recording setups. However, our data analysis is reproducible using the provided codes and dataset.
Randomization	We used passive audio recording, so there was no randomization needed.
Blinding	We used passive audio recording, so there was no human bias in data collection.

Did the study involve field work? ☒ Yes ☐ No

Field work, collection and transport

Field conditions	The temperature loggers recorded the temperature at each recording plot every 15 minutes during the experimental periods. We didn't conduct the field recording during rainy days because of the high noise produced by rain drops. Temperature data has been provided in the manuscript (Figure 3), Dryad repository (https://doi.org/10.5061/dryad.0gb5mkm5w), and supplementary materials.
Location	The field station of the University of Mississippi at Abbeville, Mississippi, USA (34°43’ N 89°39’ W).
Access & import/export	The field work was authorized by the field station of the University of Mississippi. Collecting permits (0706161) were provided by the Mississippi Department of Wildlife Fisheries and Parks.
Disturbance	We used propylene glycol for pitfall traps to minimize the potential environmental toxicity. All the recording equipments was removed after the field work.

Reporting for specific materials, systems and methods

We require information from authors about some types of materials, experimental systems and methods used in many studies. Here, indicate whether each material, system or method listed is relevant to your study. If you are not sure if a list item applies to your research, read the appropriate section before selecting a response.

Materials & experimental systems

n/a	Involved in the study	
☒	☐	Antibodies
☒	☐	Eukaryotic cell lines
☒	☐	Palaeontology and archaeology
☐	☒	Animals and other organisms
☒	☐	Clinical data
☒	☐	Dual use research of concern

Methods

n/a	Involved in the study	
☒	☐	ChIP-seq
☒	☐	Flow cytometry
☒	☐	MRI-based neuroimaging

Animals and other research organisms

Policy information about studies involving animals; ARRIVE guidelines recommended for reporting animal research, and Sex and Gender in Research

Laboratory animals	This study did not involve laboratory animals.
Wild animals	We used propylene glycol for pitfall traps to minimize the potential environmental toxicity. The ground-dwelling arthropods collected by pitfall trapping were dead at the time of collection. We sorted collected specimens by the time of collection, collection date, and study plot and we preserved them in 95% ethanol for later species identification. The samples were located in the University of Nebraska-Lincoln.
Reporting on sex	Only male spiders produced vibratory courtship signals, so our results is primarily focused on male reproductive behaviors.
Field-collected samples	We preserved collected specimens from pitfall traps in 95% ethanol for later species identification. The samples were located in the University of Nebraska-Lincoln.
Ethics oversight	No special protocols are required for the handling of use of ground-dwelling arthropods in research. We nevertheless followed the suggestions of ASAB/ABS Guidelines for the use of animals in research. Collecting permits (0706161) were provided by the Mississippi Department of Wildlife Fisheries and Parks.

Note that full information on the approval of the study protocol must also be provided in the manuscript.
